# R-package agentBayes: Likelihood-based statistical methods for agent-based models

**DOI:** 10.1371/journal.pcbi.1014786

**Published:** 2026-09-15

**Authors:** Niklas Moser, Dmitri Finkelshtein, Georgy Chargaziya, Stephen J. Cornell, Sara Hamis, Jacob G. Scott, Dagim Shiferaw Tadele, Otso Ovaskainen

**Affiliations:** 1 Department of Biological and Environmental Science, University of Jyväskylä, Jyväskylä, Finland; 2 Department of Mathematics, Swansea University, Swansea, United Kingdom; 3 Department of Evolution, Ecology, and Behaviour, Institute of Infection, Veterinary, and Ecological Sciences, University of Liverpool, Liverpool, United Kingdom; 4 Department of Information Technology, Uppsala University, Uppsala, Sweden; 5 Department of Translational Hematology and Oncology Research, Cleveland Clinic, Cleveland, Ohio, United States of America; 6 Department of Medical Genetics, Oslo University Hospital, Oslo, Norway; Universite de Montpellier, FRANCE

## Abstract

Statistically analysing interacting particle systems remains challenging because the governing equations are analytically intractable. Existing solutions include moment closure methods with pseudolikelihood-based frameworks, and likelihood-free frameworks based on extensive simulations, both relying on heuristic choices whose validity is difficult to predict. As a resolution, we rigorously derive an asymptotically exact expression for the likelihood of agent-based models (ABMs) operating in continuous space and time that can be formulated as reactant–catalyst–product (RCP) models. We derive an expression for the conditional density of agents given information about the current and earlier distributions of neighbouring agents. We utilize this expression to construct an asymptotically exact likelihood that applies to both spatial snapshot and time-series data. We implement the likelihood expression and a Bayesian parameter estimation framework in the R-package agentBayes and demonstrate its utility in biological research and beyond with simulated case studies and empirical data on the evolution of cancer cell populations.

## Author summary

Many study systems across biology, finance, physics and the social sciences consist of discrete interacting agents, yet fitting agent-based models to data remains challenging as most models are too complicated to be treated analytically. We resolve this fundamental challenge and derive tractable likelihoods for a broad class of agent-based models and data, such as spatial snapshot and time-series data. Accompanied by our statistical software, we show how to apply our framework to address biological research questions at the example of the coevolution of cancer cell populations.

## Introduction

In many disciplines, a rich array of phenomena can be described by interactions of discrete entities. These entities are often called individuals or agents in biology [1], sociology [2] and finance [3], or particles in physics [4] and chemistry [5]. The governing equations of such interacting particle systems can be described through stochastic agent-level processes of local interactions, from which patterns at larger spatial and temporal scales emerge [6].

Agent-level process descriptions are well poised for probing many study systems, which has led to a rich theoretical literature on agent-based models (ABMs) [7–11]. However, relating such models to data is highly challenging due to the analytical intractability of the governing equations. As a consequence, most methods targeted to data analysis are of correlative nature and operate at the level of aggregates rather than at the level of the interacting agents. As one example, in ecology generalized linear models [12–14] or ordinations [15–17] are used to link environment and spatial predictors to species abundances or occurrences. However, these models do not directly address the underlying agent-level processes which makes the interpretation of inferred parameters difficult [18–20]. Agent-level correlative models such as statistical point process models [21,22] use agent-level data, but still infer aggregate-level patterns, e.g., moments, rather than the underlying interactions. Mechanistic models address directly the underlying processes, but even simple aggregate-level mechanistic models [23–25] typically rely either on heuristic methods or make strong assumptions, e.g., pseudolikelihoods, in order to fit them to data.

Most attempts to parametrize ABMs use likelihood-free methods, e.g., approximate Bayesian computation, which depend on heuristic choices of summary characteristics, extensive simulations or simpler surrogate models [26–30]. Besides the usage of ABMs as simulators only, attempts have been made to build likelihoods from the governing microscopic equations, i.e., stochastic differential equations or master equations. Even if such equations can be formulated, solving them to determine the probability density of a given observation is generally analytically not possible. Therefore, microscopic treatments resort to numerical integration and variational inference approaches [31]. Mesoscopic treatments take advantage of the linear noise approximation [32–34] or moment closure methods [35,36]. Applying these likelihood-based approaches requires elaborate derivations for each specific model and their suitability greatly varies among study systems, data and computational resources. Thus, there is a need for a mathematically rigorous likelihood-based framework applicable across broad classes of ABMs.

In this paper, we overcome the above-described limitations by rigorously deriving an asymptotically (in the limit of long-ranged interactions) exact tractable likelihood for the broad class of ABMs that can be formulated as continuous-space, continuous-time reactant-catalyst-product (RCP) models. Our contributions are twofold: (i) we derive novel expressions for the local agent densities that enable deriving tractable likelihoods for RCP models and (ii) we develop the R-package agentBayes that automates the application of the likelihood framework. Regarding (i), using a perturbation expansion to compute spatiotemporal moments and cumulants of any order [37], we derive expressions for the expected local densities of agents unconditional and conditional on past and/or current observations of agents of the same or other types. Using the conditional expectation, we derive the joint probability of data observed over multiple locations and feed this likelihood into a Bayesian framework to infer model parameters such as the strength and length scale of interactions. Regarding (ii), we develop the accompanying R-package agentBayes that automates the derivation of the likelihood expression for any RCP model, facilitating the application of our framework with minimal technical effort (Fig 1). We illustrate the utility and general applicability of the approach with simulated and empirical snapshot and time-series data in both transient and stationary regimes.

**Fig 1:**
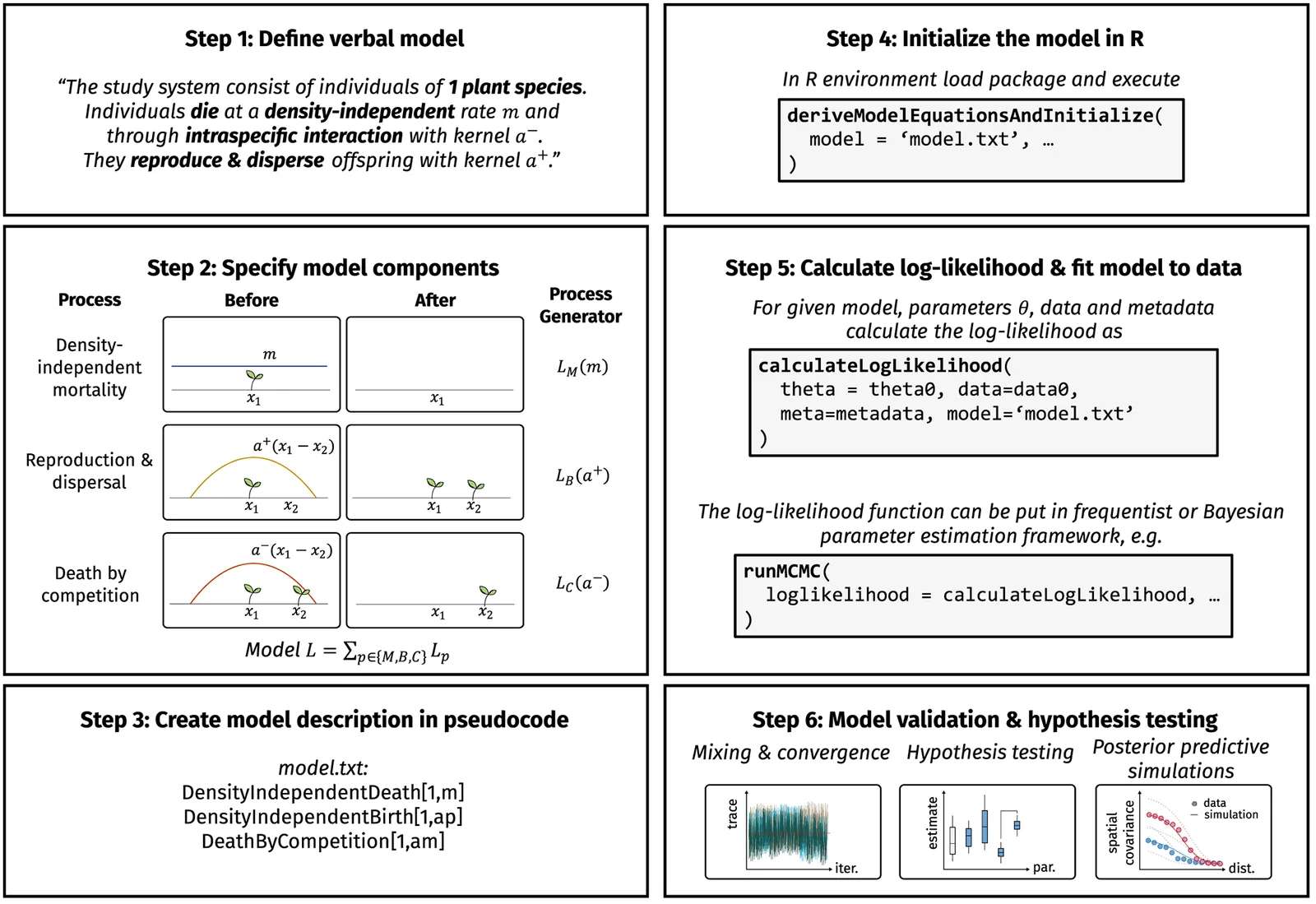
Workflow of likelihood-based analysis of agent-based models using the R-package agentBayes. **Step 1** verbally defines a model through information on the types of agents and their interactions. **Step 2** extracts from the verbal model definition the model components, which are described through the rates at which each process takes place and the state of the system before and after the process occurs [37]. **Step 3** converts the model components into computer-readable pseudocode saved in a text file. **Step 4** loads the R package, derives the governing equations, and initializes the numerical solvers. **Step 5** calculates the log-likelihood of the data, given the model and a parameter vector θ, and applies this likelihood in a Bayesian parameter estimation framework. **Step 6** validates the fitted model by, e.g., comparing it with independent data and uses the model for inference and prediction.

## Results

### A general expression of the likelihood of data under an RCP model

The RCP framework encompasses a large family of models of interactive particles in continuous space and time [37]. As one seldom has data on the exact spatiotemporal distribution of agents of all types at all times, we developed our methods for sample plot data. Thus, we assume that a researcher has recorded the number of agents (of all or some types) in discrete and finite-size plots i at locations $x_{i}$. In the examples of this paper, we model snapshot data by assuming that the numbers of agents in sample plots follow the Poisson distribution $y_{s,i}\sim \text{ Poisson}\left(\delta_{i}r_{s,i}\right)$, where $y_{s,i}$ is the number of agents of type s observed in the sample plot i,$\;r_{s,i}\;$ is the (conditional) expectation of the density of agents, generated by an RCP model, of type s at location $x_{i}$ as defined below, and $\delta_{i}$ is the area of the sample plot. We note that even if the assumption of the Poisson distribution may not hold for many models for large sample plots, it typically holds when the area of the sample plot $\delta_{i}$ is small compared to the density of agents so that the number of agents per plot is small. In the case of time-series data, we model directly the transition rates, i.e., the rates at which new agents appear and existing agents disappear, as explained in more detail below.

The cornerstone for deriving the likelihood of data for the general class of RCP models is the observation that it is possible to predict the density of agents not only in terms of its expectation, but also in terms of its covariance structure. This is non-trivial, as RCP models are stochastic agent-based models operating in continuous space and continuous time, and hence the spatiotemporal distribution of agents is an emergent property of the model rather than directly a state-variable. If simulating realizations of an RCP model, the number of agents of all types s that are present in all sample plots i at the time of sampling will vary and hence can be considered as a multivariate random variable denoted by $𝐘={\left\{Y_{s,i}\right\}}_{s,i}$. We denote by $𝐙={\left\{Z_{s,i}\right\}}_{s,i}$ the multivariate random variable of realized agent densities of all agent types s in all sample plots i, with $Z_{s,i}=Y_{s,i}/\delta_{i}$ defined as the number of agents of type s in sample plot i, divided by the area of the sample plot $\delta_{i}$. Using mathematical methodology developed for RCP models [37], the expected value of the density at location i can be predicted as


$$\begin{array}{c} \begin{array}{c} E\left[Z_{s,i}\right]=q_{s,i}+\varepsilon^{d}p_{s,i}+o\left(\varepsilon^{d}\right).\; \end{array} \end{array}$$ (1)

Equation (1) is a first-order perturbation around a non-spatial mean-field model, where $q_{s,i}$ is the mean-field prediction, $p_{s,i}$ is the first-order correction term, ε describes the assumed spatial scale of interactions (with ε→0 corresponding to global interactions), and d is the dimensions of the space (commonly d=2 in ecological applications). The notation $o\left(\varepsilon^{d}\right)$ is used for terms of strictly higher order in ε than d, and hence which vanish faster as ε→0 than $\varepsilon^{d}$. Closed-form expressions are available for both $q_{s,i}$ and $p_{s,i}$ for the general family of RCP models. Furthermore, the variance of the density can be predicted as


$$\begin{array}{c} \begin{array}{c} \text{Var}\left(Z_{s,i}\right)=\frac{q_{s,i}+\varepsilon^{d}p_{s,i}}{\delta_{i}}+o\left(\varepsilon^{d}\right), \end{array} \end{array}$$ (2)

and the covariance between the densities of two types of agents in two locations as


$$\begin{array}{c} \begin{array}{c} \text{Cov}\left(Z_{s_{\text{1}},i_{\text{1}}},Z_{s_{\text{2}},i_{\text{2}}}\right)=\varepsilon^{d}g_{s_{\text{1}},s_{\text{2}}}^{\left(\text{2}\right)}\left({\varepsilon x}_{i_{\text{1}}},\varepsilon x_{i_{\text{2}}}\right)+o\left(\varepsilon^{d}\right). \end{array} \end{array}$$ (3)

A closed-form expression for the first-order approximation of the two-point spatial cumulant $g_{s_{\text{1}},s_{\text{2}}}^{\left(\text{2}\right)}$ is available for the general family of RCP models.

We consider the random vector of agent densities 𝐙 to follow a multivariate distribution whose distributional form is unknown, but the expectation μ=E[𝐙] and variance-covariance matrix Σ with elements $\sigma_{j_{\text{1}}j_{\text{2}}}:=\text{ Cov}\left(Z_{{s(j_{\text{1}}),i(j}_{\text{1}})},Z_{{s(j_{\text{2}}),i(j}_{\text{2}})}\right)$ known, as they are defined by equations (1)–(3). Here, we denote the number of a given agent type s(j) at a given sample plot i(j), with the index j=1…n going over all combinations of agent types and sample plots.

Accounting for the mathematically predicted variance-covariance structure enables one to predict the expected value of the density $Z_{s,i}$ conditional on some of the observed densities $z_{s,i}:=\;y_{s,i}/\delta_{i}$, with the observed number of agents $y_{s,i}$ being a realisation of the random variable $Y_{s,i}$, and hence beyond the marginal expectation of equation (1). We define such a conditioning generally through the equation 𝐔𝐙=𝐛, where 𝐛:=𝐔𝐳 denotes the observed realization of the random variable 𝐔𝐙, 𝐔 is some k×n matrix of rank k. Then the conditional expectation of 𝐙 can be formulated as


$$\begin{array}{c} \begin{array}{c} E\left[𝐙|𝐔𝐙=𝐛\right]=\mu +𝐂\left(𝐛-𝐔\mu \right)+\text{o}(\varepsilon^{d}), \end{array} \end{array}$$ (4)

where $𝐂=\;\Sigma {𝐔}^{T}{\left(𝐔\Sigma {𝐔}^{T}\right)}^{-\text{1}}$.

As exemplified below, when predicting a particular observation j, in some cases one wishes to condition the prediction on all other observations, whereas in other cases only on a particular subset. To enable a general approach, we denote by ${𝐔}^{\left(j\right)}$ and ${𝐛}^{\left(j\right)}={𝐔}^{\left(j\right)}𝐳$ the conditioning applied when predicting the observation $z_{j}$, resulting in the matrix ${𝐂}^{\left(j\right)}$. We note that ${𝐂}^{\left(j\right)}$ of equation (4) does not need to be computed from scratch for each j, but its values can be updated computationally efficiently using simple recursive formulae. We further note that as we wish to predict only the element $z_{j}$ (instead of the entire vector 𝐳) conditional on ${𝐔}^{\left(j\right)}$ and ${𝐛}^{\left(j\right)}$, one needs to include only the jth rows of the matrix ${𝐂}^{\left(j\right)}$ when applying equation (4). As further exemplified below, the process may be partially observed, so that the data may not contain all agent types, even if they are part of the model dynamics and thus remain components of the multivariate random variable 𝐙.

The procedure described above produces an asymptotically exact likelihood in the limit where the spatial scale parameter ε is small, so that equations (1)–(3) become accurate, and the number of agents per cell can be assumed to follow a Poisson distribution. For cases where the Poisson distribution is not appropriate, one can utilize other distributional assumptions. As one alternative, we derive an expression for the likelihood assuming the multivariate normal distribution, whose covariance structure we assume to follow equations (1)–(3). Due to the Central Limit Theorem, the multivariate normal likelihood is likely to be a good approximation in case there are many agents within a sampling plot, e.g., when the sampling plot size is large. The multivariate normal likelihood is computationally cheaper to evaluate numerically than the Poisson distribution, as it can be computed directly from the covariance matrix without the need to construct it incrementally cell by cell. In cases that may conform directly to neither the multivariate normal likelihood nor the Poisson likelihood, it can be instructive to compare the parameter estimates provided by both alternatives. We illustrate the accuracy of the likelihood approximation by comparing likelihood profiles for the simulated data example, see S1 File.

We next illustrate the general framework of equations (1)–(4) with three case studies, out of which the first two are based on simulated data of a simple toy model, and the third one on a more complex model applied to empirical data.

### Case study one: Snapshot data on a single species

As a toy model, we consider the spatial and stochastic logistic model (SSLM) which has been extensively studied in theoretical ecology [9,10,38,39]. This model consists of a single agent type for which the dynamics are governed by three processes: (i) density-independent death, (ii) density-dependent death, and (iii) dispersal and reproduction. Density-independent death occurs at rate m. For an agent at location x, density-dependent death occurs at rate $\sum_{y\;\in \;\gamma \setminus x}a^{-}(x-y),$ where γ denotes the current set of agents, and the spatial kernel function $a^{-}$ describes how much the presence of another agent at location y increases the death rate (e.g., due to competitive interactions) of an agent located at x, with the kernel’s integral $A^{-}=\int a^{-}(x)dx$ modelling the overall strength of competition. Reproduction and dispersal is defined by the spatial kernel function $a^{+}$, such that an individual at location x produces offspring to the vicinity of y with the per unit-area rate $a^{+}(x-y)$, and $A^{+}=\int a^{+}(x)dx$ is the total rate at which each agent produces offspring.

We simulated a particular parameterization of the model, with Fig 2a illustrating the distribution of agents at the end of a simulation. We assumed that data 𝐲 on counts of agents were available from sample plots that form a regular grid (blue area in Fig 2a) of N grid cells, each of equal size δ, so that the observed density is 𝐳=𝐲/δ. In the case of a single agent type, the index s in $y_{s,i}$ is redundant, and thus i(j)=j, and the dimension of 𝐲 is simply the number grid cells, n=N. We partition the joint probability of observing the data into the product of conditional probabilities as follows:

**Fig 2:**
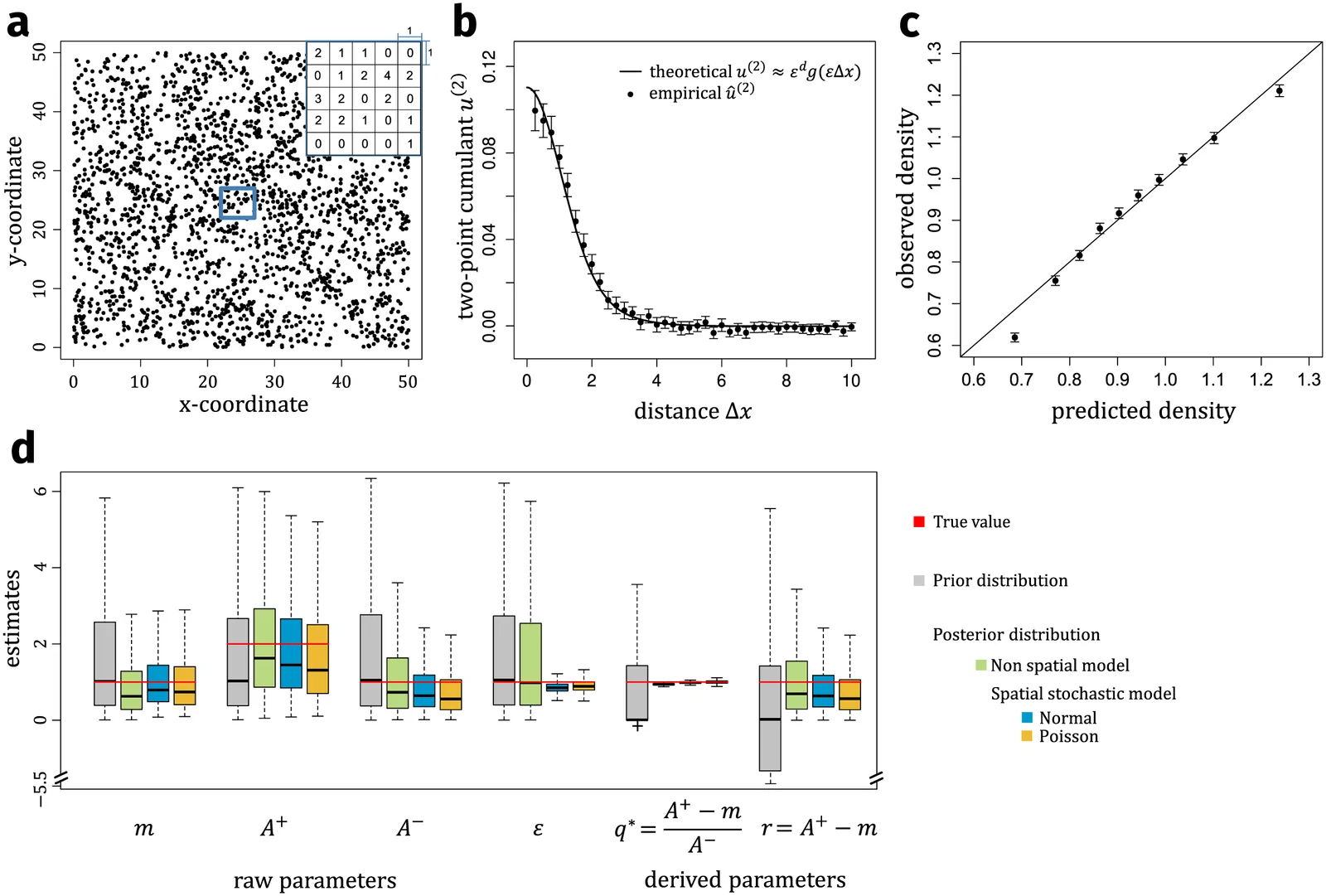
Results of the case study on snapshot data on a single species. **a**, Snapshot of the simulation at the final time t=6, where agents are shown by black dots. The blue area showcases the discretization of the point pattern with grid cell size δ=1 used in **c**-**d**. **b**, Comparison of the empirical two-point cumulant (dots) with its theoretical expectation (line). Here the empirical values represent averages over 100 replicated simulations, and the error bars represent ± 2 standard errors. **c**, Comparison of predicted densities (horizontal axis) and observed densities (vertical axis) in grid cells of the discretized domain of the 100 replicated simulations. In this panel the predicted values are based on conditioning on all other observations, even if in the likelihood approach (equation (5)) this is not the case. The grid-cell level data have been binned to ten equally sized bins based on the predicted values, and for each bin the mean ±2 standard errors are shown. **d**, Comparison of prior (grey) and posterior distributions for the raw and derived model parameters with snapshot data of one replicate. Derived parameters consist of the stationary population density $q^{*}$ and growth rate r. The posterior is computed either based on the non-spatial model, i.e., mean-field approximation (green; obtained by setting ε=0 in equations (1)-(3)) or the first-order perturbation approximation using either the likelihood of the multivariate normal distribution (light blue) or the Poisson distribution as in equations (5)-(7) (yellow; obtained by estimating the value of ε in equations (1)-(3)). True parameter values are indicated by red lines.


$$\begin{array}{c} \begin{array}{c} \text{Pr}\left(𝐘=𝐲\right)=\text{Pr}\left(Y_{\text{1}}=y_{\text{1}}\right)\prod_{j=\text{2}}^{n}\text{Pr}\left(Y_{j}=y_{j}|Y_{k}=y_{k}\;\forall k<j\right). \end{array} \end{array}$$ (5)

We thus assume some ordering of observations j, and predict the number of agents $y_{j}$ for observation j conditional on the observed data on all the preceding observations. As we assume that the data are Poisson distributed, these terms can be expressed as


$$\begin{array}{c} \begin{array}{c} \text{Pr}\left(Y_{j}=y_{j}|Y_{k}=y_{k}\;\forall k<j\right)\propto \lambda_{j}^{y_{j}}\text{exp}\left(-\lambda_{j}\right),\; \end{array} \end{array}$$ (6)

where


$$\begin{array}{c} \begin{array}{c} \lambda_{j}=\delta E\left[Z_{j}|Z_{k}=z_{k}\;\forall k<j\right]. \end{array} \end{array}$$ (7)

Note that the unconditional probability $\text{Pr}\left(Y_{\text{1}}=y_{\text{1}}\right)$ is given by equation (6) with $\lambda_{\text{1}}=\delta E\left[Z_{\text{1}}\right]$ where $E\left[Z_{\text{1}}\right]$ can be computed by equation (1). The conditional expectations in equation (7) can be computed by equation (4) by letting ${𝐔}^{\left(j\right)}$ be the (j−1)×n matrix obtained by including from the n-dimensional identity matrix the first j−1 rows, and ${𝐛}^{\left(j\right)}$ is the j−1 first elements of 𝐳. Since calculations of the joint probability in equations (5)–(7) can be computationally expensive we introduce in our examples two alternative approaches, a sliding window and a multivariate normal approximation.

Fig 2a shows the emerged point pattern of one simulation of the SSLM where the small blue panel showcases the discretization used in the analysis. Fig 2b illustrates how the two-point spatial cumulant captures the spatial aggregation in the distribution of agents, with the theoretical prediction matching well with the empirically observed pattern. While comparisons between predicted and observed cumulants such as those of Fig 2b have been published earlier [37,39], the new results of this paper are illustrated in panels c and d of Fig 2. Namely, panel c illustrates that the analytical predictions of the conditional density (equation (4)) match well with the empirically observed densities, and panel d illustrates that the likelihood approach (equation (5)) leads to accurate parameter estimates. As shown in Fig 2d, the first-order perturbation approximation leads to more accurate parameter estimates than the non-spatial mean-field approximation, especially for the spatial scale parameter ε about which the mean-field model is completely uninformative. Fig 2d indicates that a single spatial snapshot, summarized through the moments considered here, does not contain sufficient information to estimate some of the raw model parameters. This non-identifiability is structural rather than practical (e.g., to leading order, m and $A^{+}$ enter the model dynamics mostly through $r=A^{+}-m$), and is reflected in the relatively small reduction in uncertainty from prior to posterior distributions. Furthermore, strong posterior correlations between several raw model parameters suggest limited parameter identifiability (see S1 File). We note that while equation (5) is exact and thus does not depend on the ordering of the grid cells, equations (1)–(3) are approximations and hence the likelihood function may depend on the ordering. We show in the S1 File that different orderings, however, lead to very similar likelihood profiles, and thus the method is robust with respect to the ordering.

### Case study two: Time-series data on a single species

We next expand the first case study to demonstrate how the general framework of equations (1)–(4) can be applied to time-series data. We assume that in addition to collecting the count data $y_{t_{\text{1}},i}$ at time $t_{\text{1}}$, the researcher returned to the same study area at time $t_{\text{2}}=t_{\text{1}}+\Delta t$ and collected the count data $y_{t_{\text{2}},i}$. We consider two scenarios, which we call the marked and the unmarked agents. In the case where the researcher marked all agents during the first survey, the data involves direct information about the transition, i.e., how many agents were born and died between the two surveys. We model the number of new agents by $y_{+,i}\sim \text{ Poisson}\left(\delta_{i}b_{i}\right)$, where $b_{i}$ is the predicted per-unit-area birth rate for sample plot i over the time period between the two surveys. We model the number of dead agents by $y_{i,-}\;\sim \text{Bin}\left(y_{t_{\text{1}}},d_{i}\right)$, where $d_{i}$ is the predicted probability by which an individual at plot i will die between the two surveys. In case of the unmarked individuals, there remains some uncertainty between the balance of births and deaths. For example, if one agent was seen in the first survey and two in the second survey, it may be that either none died and one was born, or that one died and two were born. Thus, in the case of unmarked individuals, the likelihood includes summation over such sources of uncertainty so that we, in other words, integrate over all possible pathways that could lead to a certain realization.

In some cases, the joint likelihood L of the data collected at times $t_{\text{1}}$ and $t_{\text{2}}$ consists of the product $L=L_{\text{1}}L_{\text{1}\to \text{2}}$, where $L_{\text{1}}$ is the likelihood of the snapshot data at $t_{\text{1}}$, and $L_{\text{1}\to \text{2}}$ is the likelihood of the observed transition from $t_{\text{1}}$ to $t_{\text{2}}$. In some other cases, the likelihood may consist solely of the transition $L_{\text{1}\to \text{2}}$, for example if the initial condition before the snapshot $t_{\text{1}}$ is unknown, or if the distribution of agents at $t_{\text{1}}$ is fabricated as will be the case with the empirical data on cancer cells that we consider below. While we considered the likelihood of the snapshot data in the first case study where we assumed a known initial condition, here we focus solely on the transition and thus consider the likelihood $L=L_{\text{1}\to \text{2}}$.

To predict the birth ($b_{i}$) and death ($d_{i}$) rates for each grid cell i, we utilize the auxiliary model approach of Ovaskainen et al. [40], which expands each agent type into three auxiliary agent types, denoted by O, + and −. Here O are the original individuals, i.e., those who were present both in the first and the second survey, whereas + and − are respectively those individuals that appeared and disappeared between the two surveys. The time-series data (i.e., the two snapshots at $t_{\text{1}}$ to $t_{\text{2}}$) of the original model can then be reconstructed from a single snapshot of the auxiliary model:$y_{t_{\text{1}},i}=y_{O,i}+y_{-,i}$, and $y_{t_{\text{2}},i}=y_{O,i}+y_{+,i}$. The birth rate $b_{i}$ is predicted as the expected density of the + agents, $b_{i}=E[Z_{+,i}]$. The death rate is predicted by the ratio of the expected densities of the O and − agents, $d_{i}=E\left[Z_{-,i}\right]/(E\left[Z_{O,i}\right]+E\left[Z_{-,i}\right])$, with the calculation of the expectations as explained below.

To calculate for an RCP model the expectations of the multivariate random variables representing the densities $𝐙={\left({𝐙}_{𝐎},\;{𝐙}_{+},{𝐙}_{-}\right)}^{\text{T}}$ needed to compute the birth and death rates, we apply the general framework of equations (1)–(4). The index j now goes through all combinations of agent types s∈{O,+,−} and grid cells i∈I={1,…,N}, and hence n=3N. We order the combinations of agent types and grid cells j so that the indices j=1,…,N correspond to the O agents, the indices j=N+1,…,2N correspond to the + agents, and the indices j=2N+1,…,3N correspond to the − agents, each following the same ordering of the grid cells.

We condition the predictions for the case of the unmarked individuals as


$$\begin{array}{c} \begin{array}{c} E\left[Z_{O,i},\;Z_{+,i},\;Z_{-,i}\;|Z_{O,k}+Z_{+,k}=\;z_{t_{\text{2}},k}\;\forall k<i;\;Z_{O,k}+Z_{-,k}=\;z_{t_{\text{1}},k}\;\forall k\right]. \end{array} \end{array}$$ (8)

In other words, we assume that for predicting the transition rates $b_{i}\;\&\;d_{i}$ through the expectations, the researcher would use full information on the observed agent densities at the first survey ${𝐳}_{t_{\text{1}}}={𝐳}_{O}+{𝐳}_{-}$, but information on the agent densities at the second survey ${𝐳}_{t_{\text{2}}}={𝐳}_{O}+{𝐳}_{+}$ only for the grid cells preceding the focal cell. The motivation for this is a similar partitioning of the joint likelihood to product of conditionals as presented in equation (5) for the case of the snapshot. In case of the marked individuals, we condition the predictions as


$$\begin{array}{c} E\left[Z_{O,i},\;Z_{+,i},\;Z_{-,i}\;|Z_{O,k}=z_{O,k},\;Z_{+,k}=z_{+,k},\;Z_{-,k}=z_{-,k}\;\forall k<i;\;Z_{O,k}+Z_{-,k}=\;z_{t_{\text{1}},k}\;\forall k\geq i\right]. \end{array}$$ (9)

Thanks to marking the individuals, the second survey provides full information of the observed densities $z_{O,i}$, $z_{+,i}$ and $z_{-,i}$, and hence for the preceding cells it is possible to condition directly on these instead of the unions. As equation (8) and (9) condition partially on unions of agent types and hence sums of two counts on each grid cells, the corresponding rows of the matrices 𝐔 have two entries that are set to ones, the remaining entries being set to zeros.

We continued the simulation from the state shown in Fig 2a for an additional Δt=1 time units, resulting in the transition illustrated in Fig 3a. The match between the theoretically predicted and empirically observed two-point spatial cumulants among the O, + and − agents (Fig 3b) confirms that the extended auxiliary model approach of Ovaskainen et al. [40] is capable of predicting the spatiotemporal cumulants needed to construct the conditional predictions (Fig 3c). The time-series data results in much sharper estimates of the model parameters (Fig 3d), especially in the case of the marked individuals. This is expected, as the data now contains direct information about the birth and mortality rates, as well as their variation with population density. Compared to the first case study on spatial snapshot data, raw parameters are less correlated, suggesting improved parameter identifiability (see S1 File).

**Fig 3:**
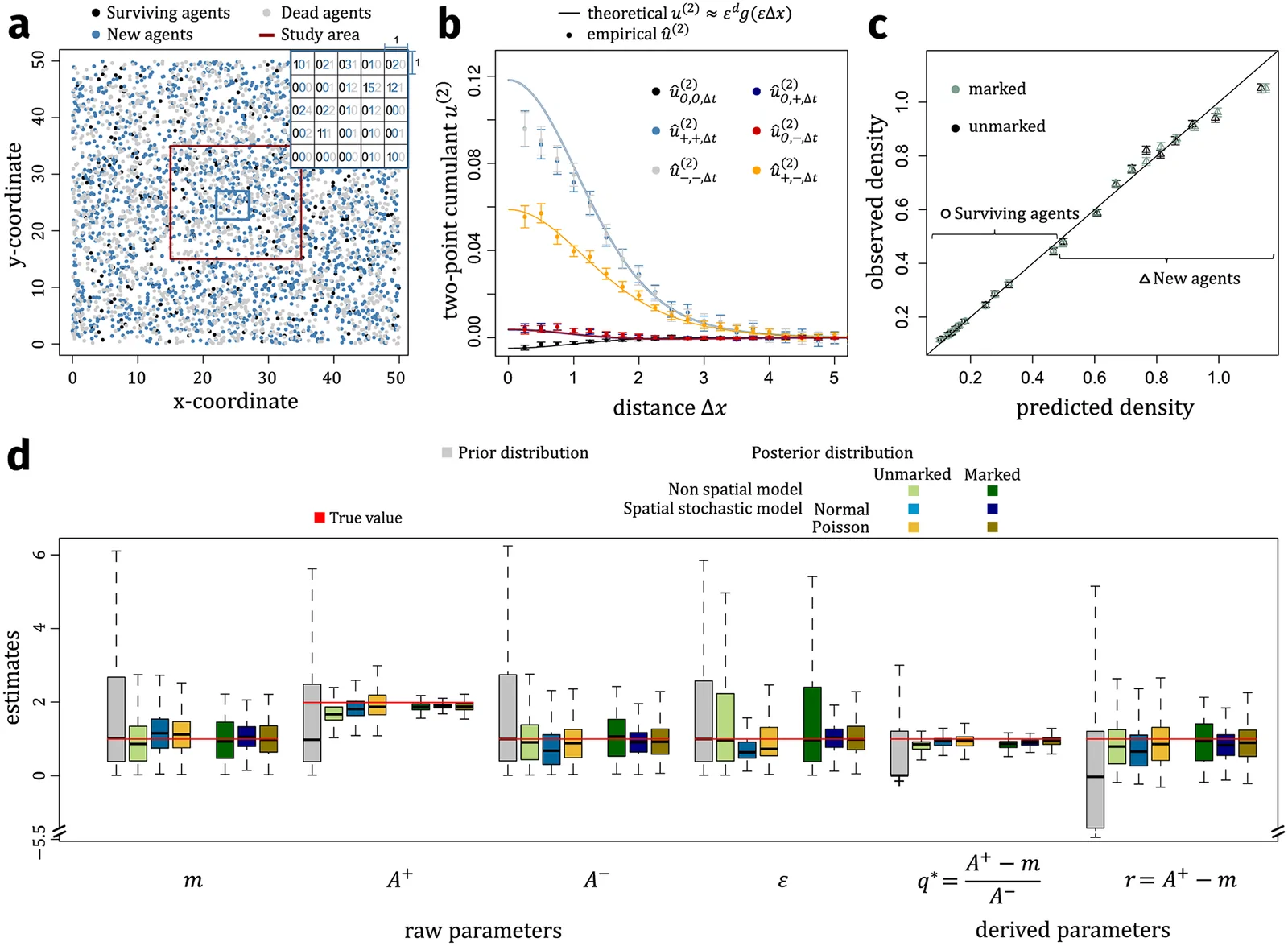
Results of the case study on time-series data on a single species. **a**, Snapshot of the simulation at time $t_{\text{1}}=\text{6}$ and $t_{\text{1}}+\Delta t=\text{7}$, where surviving agents, i.e., of type O, are shown by black dots, new agents, i.e., of type +, by blue dots and dead agents, i.e., of type −, by grey dots. The study area used in the analysis is indicated by the red square and the blue square shows the discretization used for **c**-**d**. **b**, Comparison of the empirical two-point cumulants (dots) with their theoretical expectations (line) calculated from the point pattern in the study area. **c**, Comparison of predicted densities (horizontal axis) and observed densities (vertical axis) in grid cells of the discretized domain. The grid-cell level data have been binned to ten equally spaced bins based on the predicted values, and for each bin the mean and its standard error is shown. Predictions and observations for the case of unmarked agents are illustrated in black, and for the case of marked agents in green. Densities of surviving agents are indicated by dots and densities of new agents by triangles. **d**, Comparison of prior (grey) and posterior distributions for the model parameters, the latter computed either based on the non-spatial model, i.e., mean-field approximation (green) or the spatial stochastic model using multivariate normal assumption (blue) or the Binomial and Poisson assumption (yellow). Posterior distributions are shown for unmarked agents (light colors) and marked agents (dark colors). Derived parameters consist of the stationary population density $q^{*}$ and growth rate r. True parameter values are highlighted by the solid red lines.

### Case study three: Experimental data on evolution of cancer cell populations

We demonstrate the utility of our framework to analyse empirical data using as example the evolution cancer cells (PC9 non-small lung cancer cells; see Fig 4). Our data consists of time-evolutions of drug-sensitive and drug-resistant cancer cells on experimental plates (1903 μm x 1404 μm windows of 6400 μm diameter wells in a Corning, USA 96-well plate to avoid boundary effects) with RPMI-1640 (10% Fetal Bovine Serum; 1% penicillin and streptomycin supplement) as growth medium. The experiments were conducted for three replicates of each of nine different drug concentrations (0, 0.27, 0.8, 2.5, 7.4, 22.2, 66.7, 200 and 600nM of the EGFR-inhibitor Gefitinib (Cayman, USA) per plate; homogeneously distributed) and three initial conditions (drug-resistant cells only, drug-sensitive cells only, both drug-resistant and drug-sensitive cells) (Fig 4a). The cancer cells were seeded into differential initial configurations, and their time evolution was then followed by imaging the plate every 4 hours for 92 hours and by extracting from each image the coordinates of the two types of cells (Fig 4b). The data do not enable identifying individual cancer cells and hence we will apply the framework for time-series data of unmarked individuals.

**Fig 4:**
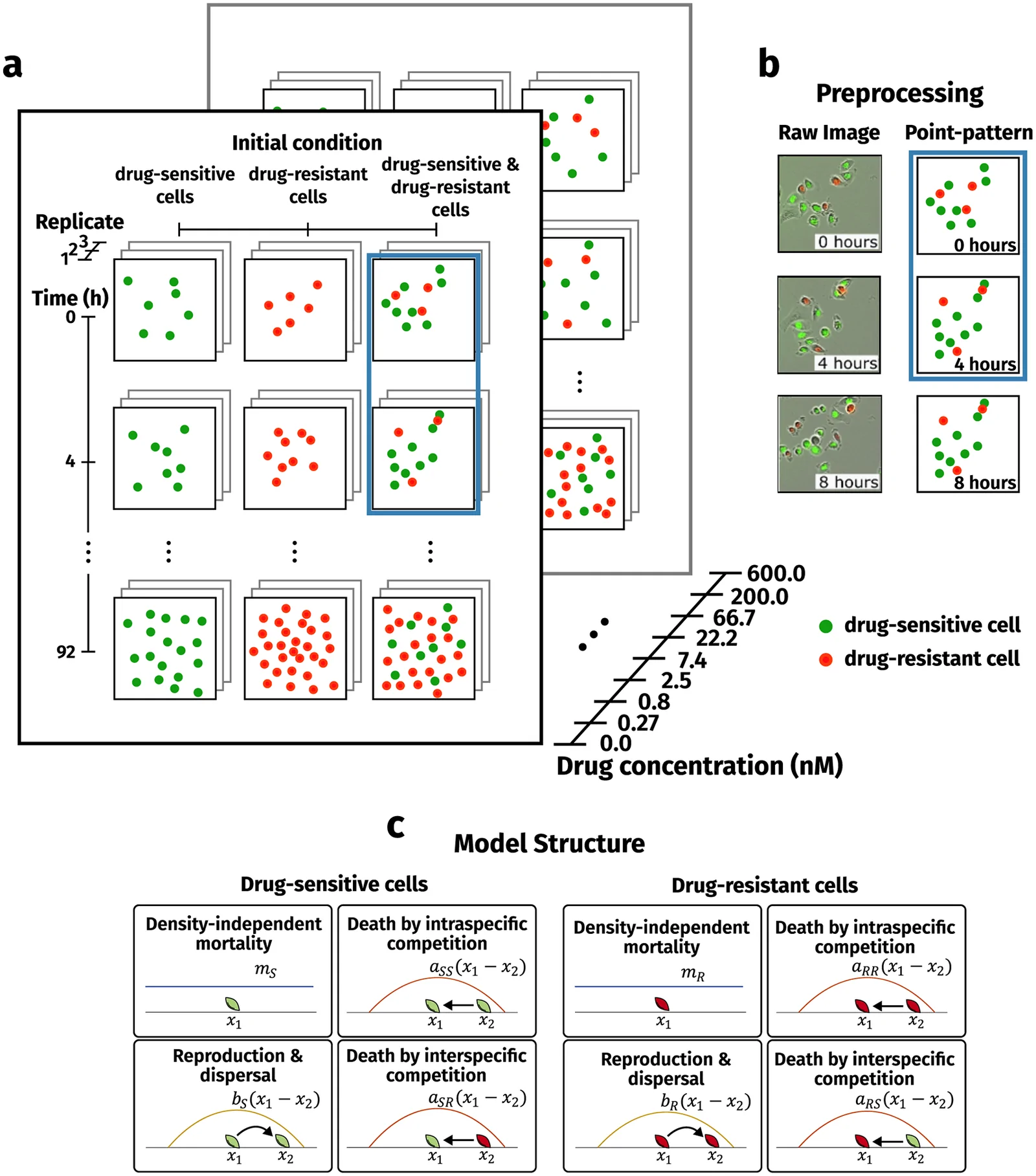
Schematic overview of experimental data & model structure of the cancer case study. **a**, The dataset comprises data for 9 different drug concentrations. For each drug concentration, experiments were conducted using 3 different initial conditions: only drug-sensitive cells seeded initially, only drug-resistant cells seeded and both together. For each of the 3 initial conditions 3 replicates were produced. Each replicate was imaged every 4 hours for 92 hours in total. For our analysis we randomly assign 2 replicates to training and the remaining replicate to test. **b**, Images of cells were converted to spatiotemporal point pattern data with coordinates for each individual cell. From the imaging, individuals could not be tagged. **c,** The model includes two agent types: drug-sensitive & -resistant cells. The dynamics of cells of each type are governed by 4 processes: density-independent mortality, death-inducing intra- & interspecific competition and reproduction & dispersal of offspring. We allow dispersal of the two types to vary in their length scale. For simplicity we assume competition to occur at the same length scale for the two types of agents.

We denote by R drug-resistant and by S drug-sensitive cells (Fig 4c). We assume that the competitive effect of cancer cells for space and resources, e.g., growth factors, is encoded in the interaction matrix $𝐀=[\begin{array}{cc} A_{RR} & A_{RS}\\ A_{SR} & A_{SS} \end{array}]$, where $A_{MN}$ denotes the total death-inducing competitive effect of cells of type N on cells of type M. Specifically, an agent of type M∈{R,S} at location x dies at rate $\sum_{y\;\in \;\gamma_{M}\setminus x}l_{a}^{-d}a_{MM}\left(\frac{x-y}{l_{a}}\right)+\sum_{y\;\in \gamma_{N}}l_{a}^{-d}a_{MN}\left(\frac{x-y}{l_{a}}\right),$ where $\gamma_{M}$ and $\gamma_{N}$ denote the set of agents of type M and N≠M, $a_{MM}$ and $a_{MN}$ are the spatial kernels of intra- and interspecific competition with overall strength $A_{MM}=\int a_{MM}(x)dx,\;A_{MN}=\int a_{MN}(x)dx,$ and $l_{a}^{-d}\;$is the length scale at which any interaction occurs relative to the length scale of reproduction and dispersal of cells of type S. Additionally, we let the dynamics of the two cell types be governed by two processes: (i) reproduction and dispersal, and (ii) density-independent mortality. For a cell of type M∈{R,S} at location x, the per-unit area rate at which it reproduces and disperses offspring to the vicinity of y is given by the spatial kernel $l_{b_{M}}^{-d}b_{M}\left(\frac{x-y}{l_{b_{M}}}\right)$ with the total rate $B_{M}=\int b_{M}(x)dx$ and length scale $l_{b_{M}}$ relative to the length scale of reproduction and dispersal of cells of type S. Density-independent death occurs at rate $m_{M}$. Hence, our model in this case study is an extension of the SSLM in the previous case studies but with agents of two types and all pairwise interactions. We fitted the model separately for each of the nine drug concentrations, leaving one replicate of each of the three initial conditions as test data and using the remaining two replicates as training data used to fit the model.

Posterior predictive simulations suggest that, despite its simplicity, the model was satisfactorily capable of capturing the dynamical behaviour of the system, with parameter values estimated from the training data generalizing also to the test data. We illustrate comparison between a single test dataset and posterior predictive simulations by visual comparison between realizations (Fig 5a and 5b), time-evolution of densities (Fig 5c), and spatial covariances in a snapshot (Fig 5d). Comparing predictive posterior data with the actual data showed greatly improved predictive performance in the posterior as compared to the prior parameterization, almost equally for the training and test data (Fig 5e and 5f showing the reduction in root mean squared error). In terms of the model’s assessment of uncertainty, we found the coverage probabilities be relatively close to the nominal values, yet indicating a level of undercoverage (Fig 5g and 5h), suggesting that the model is likely to miss some processes that generate variation in the data. As an additional validation, the S1 File demonstrates the extent by which the framework recovers the parameters from data generated by posterior predictive simulation.

**Fig 5:**
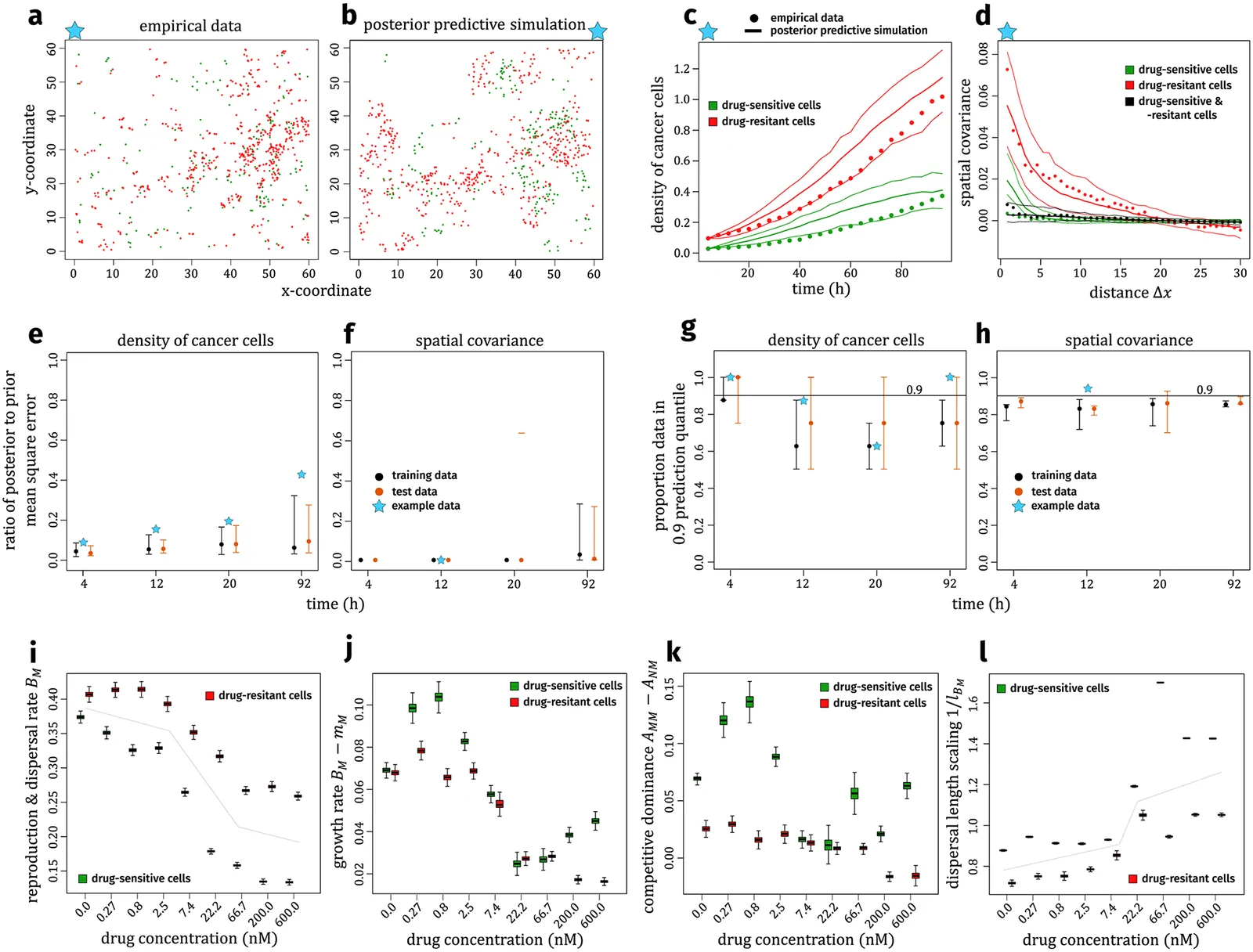
Results of the case study on the evolution of cancer cell populations. In **a**-**b** we compare empirical data with one posterior predictive simulation at 12 hours. **c**-**d**, For the example replicate marked in **a**-**h** with the blue star, we compare in **c**-**d** empirical density and spatial covariance at 12 hours with the 0.9 quantile and median of the posterior predictive simulations. **e-h,** Validation of the predictive performance for the 9 models (1 model per drug concentration) on training and test data at 4 time points. Each model was fitted using data on 3 initial conditions x 2 replicates. In **e**-**f** we calculated for each model and training/test dataset the ratio of mean square error from posterior to prior predictive simulations considering cancer cell density and spatial covariances. We show the median, upper and lower quartile over all 9 models. In **g**-**h** we take for each model of empirical data that lies within the 0.9 quantile of the posterior predictive simulations for cancer cell density and spatial covariance. We show the median, upper and lower quartile over all 9 models. **i**-**l**, from the posterior distribution we compare raw and derived model parameters of the two cancer cell types with increasing drug concentration. Here, we denote the cancer type by M,N∈{S,R} with N≠M. In **i**-**j**, we compare the rate of reproduction and unrestricted growth rate, in **k** the competitive dominance calculated as the difference of intraspecific competition and interspecific competition, and in **l** we compare the scaling of the length of dispersal $\text{1}/l_{B_{M}}$ where $l_{B_{M}}$ is the **l**ength at which dispersal occurs for cancer cells of type M.

In terms of inference, we found that with increasing drug concentration, the reproduction rate of drug-sensitive cells decreases more than drug-resistant cells (Fig 5i). Whereas the growth rate for drug-resistant cells decreases with increasing drug concentration, for drug-sensitive cells the growth rate follows a more complex pattern (Fig 5j). This pattern is consistent with the pattern of the intra- and interspecific competition for drug-sensitive cells, which are overall less competitively dominant than drug-resistant cells (Fig 5k). We further found the length scale of dispersal decreases for both cell types with increasing drug concentration and overall disperse drug-sensitive cells more locally offspring than drug-resistant cells (Fig 5l).

## Discussion

The framework presented in this paper is to our knowledge the first mathematically rigorous likelihood-based framework that applies to a broad class of ABMs. To date, fitting ABMs to data has largely relied on simulation-based approaches [28,29], typically fitted to summary statistics such as spatiotemporal patterns in the data. The method presented here overcomes the need to use extensive simulations and heuristic pattern-oriented criteria by directly tackling the agent-level processes with novel analytical machinery. Compared to recent approaches that use analytical approximations to the intractable likelihood of ABMs [32,33,41–43], our method generalizes not only to a broader class of models, i.e., continuous space, continuous time RCP models, but also to common data types and systems in stationary and transient regimes. Additionally, unlike previous attempts, applying our framework does not require the skills to perform laborious derivations, as our accompanying software automates the derivations. Since we rigorously derive the expressions for the conditional expectation of the density of agents using a perturbation expansion, as shown in the S1 File, our expression for the likelihood is guaranteed to be exact in the limit of long-range interactions.

Our examples provide a proof-of-concept for simulated snapshot and time-series data and showcase the utility of applying our method to empirical data to infer agent-level processes. While applying our method to snapshot data requires knowledge of either an initial state of the system or that the system is in equilibrium, time-series data overcomes this limitation by enabling the calculation of the likelihood of the transition between two observations. We illustrated how to apply our method to the cases of both marked and unmarked agents, the latter is the most common observational data type in many applied fields such as ecology, finance and social sciences. Compared to common spatial applications using pseudo-likelihoods, e.g., via compositional approaches [44], we showed how to construct and efficiently calculate the joint likelihood for both snapshot and time-series data. While our examples encompass only a subset of processes that can be represented as RCP models, a more exhaustive process library is presented by Cornell et al. [37]. With an experimental case study on cancer cells, we exemplified how our framework can be used to infer agent-based biological processes from empirical data. As our framework enabled computing the likelihood of the data, we could apply standard Bayesian inference for both fitting the model to the data as well as for evaluating the model fit. In this specific case study, we found that both reproductive and competitive processes occurred at local scales. This phenomenon could not have been detected by mean-field approaches that have thus far been the most common type of approach in oncological studies [45–48]. We further found that the dynamics of cell-to-cell interactions with increasing drug concentration show non-linear patterns, suggesting that not only growth factor secretion and uptake but also negative competition for space and nutrients drive the co-evolution of the cancer cell populations.

The cornerstone of our methodology is the expression of conditional agent densities, given some observed data (equation (4)). To derive the likelihood of data, this expression can be combined with an appropriate statistical distribution, the choice of which depends on the nature of the data. For the cancer case study, we assumed Poisson likelihood, which accounts for the discrete nature of count data. For the simulated case studies, we compared multivariate normal and Poisson likelihoods. We found these two to yield consistent inference, with similar median, range of values and improvement over the non-spatial model (e.g., for $A^{+},\;\varepsilon ,$ or r in Fig 3d), even if the multivariate normal likelihood does not account for the integer-valued nature of count data. This suggests that it is more important to replicate the correct mean and covariance structure than to implement the exact probability distribution. The multivariate normal likelihood is much more computationally efficient, and simpler to implement, than the Poisson likelihood, and may be more suitable when it is preferable to prioritise simplicity and efficiency over correctness. Furthermore, the multivariate normal likelihood is expected to perform well when the number of individuals per cell is large, due to the Central Limit Theorem.

Even though the cancer case study demonstrates that our framework applies to a broad class of empirical data, three challenges remain: (i) incorporation of agent-level processes that violate RCP model assumptions, (ii) scalability to large number of agent types, and (iii) analysis of data from heterogeneous spatial conditions. Regarding challenge (i), our current framework does not apply to processes that take place in discrete space and/or discrete time, or that involve agents whose behaviour is influenced by their memory. In many practical examples the Markovian assumption might suffice, e.g., if the observational scale is larger than the memory decay scale, or by accounting for the memory effect through agents switching between different types. Addressing challenge (ii), while our framework conceptually applies to an arbitrary number of agent types B, the computational costs are approximately of order $O\left(N^{\text{3}B}\right)$, where N is the number of grid cells in the domain, hence making it inefficient to analyze large community data. This may be approached by implementing more advanced techniques such as GPU parallelization. Finally, for challenge (iii), the current framework assumes homogeneous spatial conditions such as the homogeneously distributed growth media in our cancer cell case study. However, especially observational non-manipulative data often arises from heterogeneous environments, to which our current framework does not apply, unless the spatial structure, such as habitat patches, are directly included as agents in the current framework. A more direct implementation of environmental heterogeneity could likely be implemented as an extension of the work presented here, as the underlying mathematical tools for spatiotemporal cumulants have been established for both homogeneous and heterogeneous space [37].

In summary, our framework proposes a rigorously derived asymptotically exact tractable likelihood for a broad class of ABMs and for common data types in many applied research fields. We illustrated with both simulated and empirical examples how our method can be used to directly infer agent-level processes from empirical data, and to critically validate the fitted models. Since many systems in life-sciences [1,49,50] and other areas of application [2,3,51,52] consist of agent-level processes, we consider our framework to be a crucial and general advancement. Specifically, as our framework matches models with complex system dynamics, it allows to broaden system understanding and consequently make more reliable predictions.

## Methods

### Mathematical background for RCP-models

We apply the mathematical framework of Cornell et al. [37], which models discrete agents that interact with each other, e.g., through competition or reproduction and dispersal, in continuous space and continuous time. We denote the agent configuration (including their positions and marks) by γ and consider their evolution in d-dimensional space $R^{d}$ where for any bounded region Λ the number of agents is assumed to remain finite. The agent configuration γ can be expressed as the disjoint union of the configurations $\gamma_{s}$ where the index s denotes the marks s∈S of the agents. Thus,


$$\begin{array}{c} \gamma ={⨆}_{s\in \text{S}}\gamma_{s}\;. \end{array}$$ (10)

We define the space of locally finite configurations Γ


$$\begin{array}{c} \begin{array}{c} \Gamma :=\;\left\{\gamma \subset X||\gamma \cap \Lambda |<\infty ,\text{ for all }\Lambda \text{ bounded}\right\},\; \end{array} \end{array}$$ (11)

where |·|  denotes the cardinality of a set, X is the space of real-valued positions and their marks [53]. Note that for the agent configuration of a given mark s it holds that $\gamma_{s}\subset R^{d}$.

We introduce the probability measure $\mu_{t}$ on Γ to describe the state of the system. The measure μ describes the probability of the system to be in a certain state at time t, given the initial state defined at some earlier time. The system can be probed by observables F, which operate on Γ and return real-numbered values. As one example of an observable, we may let F be a function which counts the number of agents of certain type within a certain domain. The state of the system can be described by the pairing of observable F and probability measure $\mu_{t}$


$$\begin{array}{c} \begin{array}{c} \left\langle F,\mu_{t}\right\rangle :=\int_{\Gamma }F(\gamma )d\mu_{t}(\gamma ). \end{array} \end{array}$$ (12)

The evolution of the system through time t is defined through the change in observables as


$$\begin{array}{c} \begin{array}{c} \frac{d}{dt}\left\langle F,\;\mu_{t}\right\rangle =\left\langle LF,\;\mu_{t}\right\rangle , \end{array} \end{array}$$ (13)

where the model is specified through the Markov operator L. This Markov operator is defined by the system processes P as


$$\begin{array}{c} L=\sum_{p=\text{1}}^{P}L_{p},\; \end{array}$$ (14)

where each process p describes a specific type of interaction among the agents, such as competition or reproduction.

The mathematical framework of Cornell et al. [37] allows to derive exact equations for the evolution of spatial correlation functions of RCP models. The first-order spatial correlation function $k_{s,t}^{\left(\text{1}\right)}(x)$ measures the expected density of agents with mark s at location x and time t. The expected number of agents with mark s in an area Λ at time t can be computed as


$$\begin{array}{c} \begin{array}{c} E\left[\left|\gamma_{s,t}\cap \Lambda \right|\right]\;=\int_{\Lambda }k_{s,t}^{\left(\text{1}\right)}(x)dx. \end{array} \end{array}$$ (15)

The second-order correlation function $k_{s_{\text{1}},s_{\text{2}},t}^{\left(\text{2}\right)}(x_{\text{1}},x_{\text{2}})$ can be used to compute the product of the number of agents with mark $s_{\text{1}}$ in an area $\Lambda_{\text{1}}$ and the number of agents with mark $s_{\text{2}}$ in an area $\Lambda_{\text{2}}$ at time t as


$$\begin{array}{c} \begin{array}{c} E\left[\left|\gamma_{s_{\text{1}},t}\cap \Lambda_{\text{1}}\right|\left|\gamma_{s_{\text{2}},t}\cap \Lambda_{\text{2}}\right|\right]\;=\int_{\Lambda_{\text{1}}}\int_{\Lambda_{\text{2}}}k_{s_{\text{1}},s_{\text{2}},t}^{\left(\text{2}\right)}\left(x_{\text{1}},x_{\text{2}}\right)dx_{\text{1}}dx_{\text{2}}+\delta_{s_{\text{1}},s_{\text{2}}}\int_{\Lambda_{\text{1}}\cap \Lambda_{\text{2}}}k_{s_{\text{1}},t}^{\left(\text{1}\right)}(x)dx, \end{array} \end{array}$$ (16)

where Kronecker’s delta $\delta_{s_{\text{1}},s_{\text{2}}}$equals one if $s_{\text{1}}=s_{\text{2}}$ and otherwise zero, and hence the last summand counts the self-pairs of agents for the case where the two areas $\Lambda_{\text{1}}$ and $\Lambda_{\text{2}}$ are not disjoint [40].

For each Markov operator L it is possible to derive a corresponding operator $L^{\Delta }$ which describes the evolution of the spatial correlation functions $k={\left\{k^{\left(l\right)}\right\}}_{l=\text{1}}^{\infty }$ of all orders and of all mark combinations as [37]


$$\begin{array}{c} \begin{array}{c} \frac{d}{dt}k_{t}(\eta )=\left(L^{\Delta }k_{t}\right)(\eta ),\; \end{array} \end{array}$$ (17)

where η denotes a finite configuration. Spatial cumulants are obtained by subtracting from the spatial correlations the products of the lower order terms. For example, the second-order cumulant is related to the moments as $u_{s_{\text{1}},s_{\text{2}},t}^{\left(\text{2}\right)}(x,y)=k_{s_{\text{1}},s_{\text{2}},t}^{\left(\text{2}\right)}(x,y)-k_{s_{\text{1}},t}^{\left(\text{1}\right)}(x)k_{s_{\text{2}},t}^{\left(\text{1}\right)}(y)$. For each Markov operator L it is possible to derive a corresponding operator $Q^{\Delta }$ which describes the evolution of the spatial cumulants u of all order and of all mark combinations as


$$\begin{array}{c} \begin{array}{c} \frac{d}{dt}u_{t}(\eta )=\left(Q^{\Delta }u_{t}\right)(\eta ),\; \end{array} \end{array}$$ (18)

where $L^{\Delta }=Q^{\Delta }+M^{\Delta }$, and $M^{\Delta }\;$ is a non-linear operator.

We note that typically the spatial moments and cumulants form an unclosed hierarchy and thus the systems of differential equations above cannot be exactly solved. We follow the systematic perturbation expansion proposed by Cornell et al. [37] to approximate the spatial moments and cumulants. Cornell et al. [37] rescaled all spatial kernel functions as


$$\begin{array}{c} \begin{array}{c} a_{\varepsilon }(x):=\varepsilon^{d}a(\varepsilon x),\; \end{array} \end{array}$$ (19)

which makes interactions increasingly long-ranged with ε→0 but keeps their intensity (the integrals of the kernels) constant. Following this rescaling, Cornell et al. [37] showed that the fist-order spatial correlation function can be computed as


$$\begin{array}{c} \begin{array}{c} k_{\varepsilon ,s,t}^{\left(\text{1}\right)}(x)=q_{s,t}(\varepsilon x)+\varepsilon^{d}p_{s,t}(\varepsilon x)+\;o\left(\varepsilon^{d}\right),\; \end{array} \end{array}$$ (20)

where $q_{s,t}(x)$ is the mean-field density, $p_{s,t}(x)$ is its first-order correction term and the approximation error behaves as $o\left(\varepsilon^{d}\right).$ Cornell et al. [37] further showed that the second-order cumulant follows the expansion


$$\begin{array}{c} \begin{array}{c} u_{\varepsilon ,s_{\text{1}}s_{\text{2}},t}^{\left(\text{2}\right)}(x,y)=\varepsilon^{d}g_{s_{\text{1}},s_{\text{2}},t}(\varepsilon x,\varepsilon y)+o\left(\varepsilon^{d}\right),\; \end{array} \end{array}$$ (21)

where the zeroth-order term is zero (in mean-field, there are no spatial correlations) and hence missing, and thus the leading term of the expansion $g_{s_{\text{1}},s_{\text{2}},t}(x,y)$ is of order $\varepsilon^{d}$. The perturbation expansion produces a closed system of differential equations that can be described as


$$\begin{array}{c} \begin{array}{c} \frac{d}{dt}q_{t}=H_{q}\left(q_{t}\right),\;\frac{d}{dt}p_{t}=H_{p}\left(q_{t},g_{t}\right),\;\frac{d}{dt}g_{t}=H_{g}\left(q_{t},p_{t},g_{t}\right)\;,\; \end{array} \end{array}$$ (22)

where the functions $H_{q}$, $H_{p}$ and $H_{g}$ can be derived for any RCP model using automated tools [37] and act on the S×x vectors $q_{t},p_{t}$ with entries for all marks S, and if the system is heterogeneous all locations $x\in R^{d}$, and the S×S×z matrix $g_{t}$ with entries of all pairs of marks S and all distances z=f(x,y) where $x,y\in R^{d}$ and f is the Euclidean distance.

### Derivation of the conditional expectation (equation (4))

Equation (4) is a general expression for the conditional expectation of a multivariate random variable $𝐙={\left(Z_{\text{1}},Z_{\text{2}},\;\ldots ,\;Z_{n}\right)}^{\text{T}}$, whose expectation is E[𝐙]=μ and variance-covariance matrix Σ. For any k×n matrix 𝐔 of rank k and vector 𝐛 of length k, equation (4) states that the expectation of 𝐙 conditional on 𝐔𝐙=𝐛 can be expressed as E[𝐙|𝐔𝐙=𝐛]=𝐀μ+𝐂𝐛, where $𝐂=\Sigma {𝐔}^{T}{\left(𝐔\Sigma {𝐔}^{T}\right)}^{-\text{1}}$ and $𝐀={𝐈}_{n}-𝐂𝐔$ where ${𝐈}_{n}$ denotes the n×n identity matrix. As $𝐀+𝐂𝐔={𝐈}_{n}$ and hence 𝐙=𝐀𝐙+𝐂𝐔𝐙,   it follows that


$$\begin{array}{c} E\left[𝐙|𝐔𝐙=𝐛\right]=E\left[𝐀𝐙+𝐂𝐔𝐙|𝐔𝐙=𝐛\right] \end{array}$$

$$\begin{array}{c} \begin{array}{c} =E\left[𝐀𝐙|𝐔𝐙=𝐛\right]+E\left[𝐂𝐔𝐙|𝐔𝐙=𝐛\right] \end{array} \end{array}$$ (23)

The matrix 𝐀 has been constructed so that 𝐀𝐙 and 𝐔𝐙 have zero covariance: $\text{Cov}\left(𝐀𝐙,\;𝐔𝐙\right)=𝐀\Sigma {𝐔}^{T}=\Sigma {𝐔}^{T}-𝐂𝐔\Sigma {𝐔}^{T}=\Sigma {𝐔}^{T}-\Sigma {𝐔}^{T}{\left(𝐔\Sigma {𝐔}^{T}\right)}^{-\text{1}}𝐔\Sigma {𝐔}^{T}=\text{0}$. If 𝐀𝐙 and 𝐔𝐙 would be independent, Eq. 4 would follow, as in that case


$$\begin{array}{c} \begin{array}{c} E\left[𝐀𝐙|𝐔𝐙=𝐛\right]+E\left[𝐂𝐔𝐙|𝐔𝐙=𝐛\right]=𝐀E\left[𝐙\right]+𝐂E\left[𝐔𝐙|𝐔𝐙=𝐛\right]\\ =𝐀\mu +𝐂𝐛=\mu +𝐂\left(𝐛-𝐔\mu \right). \end{array} \end{array}$$ (24)

While we cannot in the general case show their independence, we still consider equation (4) to hold approximately for the following two reasons. First, zero covariance is a necessary (yet not sufficient) condition for independence. If 𝐙 would be, e.g., multivariate normally distributed, independence would follow from zero covariance. Second, as we show in the S1 File, equation (4) holds at the level of the first-order perturbation, which is the level of approximation at which we have developed the likelihood approach here. We note that while we use in our framework only the conditional expectation, it would be possible to compute also uncertainties of such predictions through Var(𝐙|𝐔𝐙=𝐛).

### Efficient computation

#### Full conditioning of equation (4).

For large matrices 𝐔,Σ, calculating ${\left(𝐔\Sigma {𝐔}^{T}\right)}^{-\text{1}}$ to find 𝐂 is the most expensive computation in equation (4). Especially, in the case where we want to condition our prediction on observations of all other locations than the focal location or when computing the likelihood where we condition on iteratively less or more data points, and thus need to predict each data point separately. For example, if we wish to predict j data points we would need to compute the inverse j times. Instead, we propose two recursive strategies: (i) for computing the inverse when conditioning on one data point less via simple rank-one updates directly to ${\left(𝐔\Sigma {𝐔}^{T}\right)}^{-\text{1}}$ (ii) for computing the inverse when conditioning on one data point more. While (i) is needed in all our case studies (ii) is needed in the time-series case of marked individuals, as shown below. Assume we have computed the inverse of $𝐔\Sigma {𝐔}^{T}:=𝐅$ for some k×n matrix 𝐔 and want to find the inverse of ${𝐔}^{(j)}\Sigma {{𝐔}^{(j)\;}}^{T}:=𝐒$ where ${𝐔}^{\left(k\right)}$ is the (k−j)×n matrix obtained by dropping the first j rows of 𝐔. We can partition 𝐅 and 𝐕 so that ${𝐅}_{\text{1}\text{1}},{𝐕}_{\text{1}\text{1}},{𝐅}_{\text{2}\text{2}}$ and ${𝐕}_{\text{2}\text{2}}$ are square matrices as


$$\begin{array}{c} \begin{array}{c} 𝐅=[\begin{array}{cc} {𝐅}_{\text{1}\text{1}} & {𝐅}_{\text{1}\text{2}}\\ {𝐅}_{\text{2}\text{1}} & 𝐒={𝐅}_{\text{2}\text{2}} \end{array}], \end{array} \end{array}$$ (25)

and


$$\begin{array}{c} \begin{array}{c} {𝐅}^{-\text{1}}:=𝐕=[\begin{array}{cc} {𝐕}_{\text{1}\text{1}} & {𝐕}_{\text{1}\text{2}}\\ {𝐕}_{\text{2}\text{1}} & {𝐕}_{\text{2}\text{2}} \end{array}].\; \end{array} \end{array}$$ (26)

We compute ${𝐅}_{\text{2}\text{2}}^{-\text{1}}$ from 𝐅𝐕=𝐈, where 𝐈 denotes the identity matrix. Since


$$\begin{array}{c} \begin{array}{c} {𝐅}_{\text{2}\text{1}}{𝐕}_{\text{1}\text{2}}+{𝐅}_{\text{2}\text{2}}{𝐕}_{\text{2}\text{2}}=𝐈,\; \end{array} \end{array}$$ (27)

$$\begin{array}{c} \begin{array}{c} {𝐅}_{\text{2}\text{1}}{𝐕}_{\text{1}\text{1}}+{𝐅}_{\text{2}\text{2}}{𝐕}_{\text{2}\text{1}}=0,\; \end{array} \end{array}$$ (28)

and thus


$$\begin{array}{c} \begin{array}{c} {𝐒}^{-\text{1}}={𝐅}_{\text{2}\text{2}}^{-\text{1}}={𝐕}_{\text{2}\text{2}}-{𝐕}_{\text{2}\text{1}}{𝐕}_{\text{1}\text{1}}^{-\text{1}}{𝐕}_{\text{1}\text{2}}. \end{array} \end{array}$$ (29)

Note that we can compute $𝐂={𝐁𝐒}^{-1}$, where $𝐁=\Sigma {{𝐔}^{\left(j\right)}}^{T}={\left(\Sigma {𝐔}^{T}\right)}^{(j)}$ is computed by dropping the first j rows of $\Sigma {𝐔}^{T}$. Next, we want to compute ${𝐅}^{-1}=𝐕$ in the case where we have first explicitly computed ${𝐒}^{-1}={\left({𝐔}^{\left(j\right)}\Sigma {{𝐔}^{\left(j\right)}}^{T}\right)}^{-\text{1}}$. We compute 𝐕 block-wise as


$$\begin{array}{c} \begin{array}{c} {𝐕}_{\text{1}\text{1}}={\left({𝐅}_{\text{1}\text{1}}-{𝐅}_{\text{1}\text{2}}{𝐒}^{-\text{1}}{𝐅}_{\text{2}\text{1}}\right)}^{-\text{1}},\; \end{array} \end{array}$$ (30)

$$\begin{array}{c} \begin{array}{c} {𝐕}_{\text{2}\text{1}}={-𝐒}^{-\text{1}}{𝐅}_{\text{2}\text{1}}{𝐕}_{\text{1}\text{1}},\; \end{array} \end{array}$$ (31)

$$\begin{array}{c} \begin{array}{c} {𝐕}_{\text{1}\text{2}}={-\left({𝐅}_{\text{1}\text{1}}+{𝐅}_{\text{1}\text{2}}{𝐕}_{\text{2}\text{1}}{𝐕}_{\text{1}\text{1}}^{-\text{1}}\right)}^{-\text{1}}{𝐅}_{\text{1}\text{2}}{𝐒}^{-\text{1}}, \end{array} \end{array}$$ (32)

$$\begin{array}{c} \begin{array}{c} {𝐕}_{\text{2}\text{2}}={𝐒}^{-\text{1}}+{𝐕}_{\text{2}\text{1}}{𝐕}_{\text{1}\text{1}}^{-\text{1}}{𝐕}_{\text{1}\text{2}}. \end{array} \end{array}$$ (33)

From ${𝐅}^{-1}$ we calculate $𝐂=𝐃{𝐅}^{-1}$ where $𝐃=\Sigma {𝐔}^{T}=[\begin{array}{cc} \Sigma {𝐔}_{j}^{T} & \Sigma {{𝐔}^{\left(j\right)}}^{T} \end{array}]\;$and ${𝐔}_{\text{j}}$ denotes the first j rows added as first rows to ${𝐔}^{\left(j\right)}$ to obtain 𝐔.

#### Sliding window approach.

When computing the joint probability using, e.g., the partitioning in equations (5)–(7) it is required to evaluate the inverse ${𝐒}^{-\text{1}}:={\left(𝐔\Sigma {𝐔}^{T}\right)}^{-\text{1}}$ for every grid cell in the domain. However, if the domain is large compared to the distance at which the spatial covariance decays, then ${𝐒}^{-\text{1}}$ is effectively the same for a large fraction of grid cells. We can hence truncate 𝐔 and Σ to a smaller window size W and numerically calculate the inverse ${𝐒}_{W}^{-\text{1}}:={\left({𝐔}_{W}\Sigma_{W}{𝐔}_{W}^{T}\right)}^{-\text{1}}$ once for all grid cells with the same window. Clearly, this approach is computationally advantageous in the cases where W≪N where N is the number of grid cells in the domain.

#### Multivariate normal approximation.

Considering the limit where the number of agents in a grid cell is large the likelihood becomes that of the multivariate normal distribution. Here the likelihood can be directly computed once from the covariance matrix without iteration through all grid cells. This is advantageous to the approach of equations (1)–(4), since there one needs to recalculate equation (4) N times and hence its calculation time scales with the discretization of the domain and the number of agent types. Specifically, in the limit of large number of agents per grid cell the multivariate random variable $𝐙={\left(Z_{\text{1}},Z_{\text{2}},\ldots ,Z_{n}\right)}^{\text{T}}$ follows a multivariate normal distribution with mean E[𝐙]=μ and variance-covariance matrix Σ. The log-likelihood of observing densities 𝐳 has the closed form


$$\begin{array}{c} \begin{array}{c} \text{log}L\left(𝐳\right)\propto -\frac{\text{1}}{\text{2}}\left[\text{log}\left(\left|\Sigma \right|\right)+{\left(𝐳-\mu \right)}^{T}\Sigma^{-\text{1}}\left(𝐳-\mu \right)\right], \end{array} \end{array}$$ (34)

where |Σ| denotes the determinant of Σ. In the case of snapshot data, we can straightforwardly calculate μ and Σ as described in equation (1)–(3). In the case of time-series data, we compute the likelihood of the transition between $t_{\text{1}}$ and $t_{\text{1}}+\Delta t$. Hence, we condition our predictions on the observed densities of agents in $t_{\text{1}}$. Recall that in the time-series case we let $𝐙={\left({𝐙}_{O},\;{𝐙}_{+},{𝐙}_{-}\right)}^{\text{T}}$ where ${𝐙}_{O}$ is the random variable representing densities of surviving agents, ${𝐙}_{+}$ new agents and ${𝐙}_{-}$ dead agents, each of length N. We condition our predictions on observed densities of agents at time $t_{\text{1}}$, hence on 𝐛=𝐔𝐳 with $𝐔=[\begin{array}{ccc} {𝐈}_{N} & {𝐎}_{N} & {𝐈}_{N} \end{array}]$ consisting of N×N dimensional identity and null matrices, and the vector of observed densities $𝐳={\left({𝐳}_{O},{𝐳}_{+},{𝐳}_{-}\right)}^{\text{T}}$. We construct


$$\begin{array}{c} \begin{array}{c} \mu =[\begin{array}{c} \mu_{O}\\ \mu_{+}\\ \mu_{-} \end{array}],\;\Sigma =[\begin{array}{ccc} \Sigma_{O,O} & \Sigma_{O,+} & \Sigma_{O,-}\\ \Sigma_{+,O} & \Sigma_{+,+} & \Sigma_{+,-}\\ \Sigma_{-,O} & \Sigma_{-,+} & \Sigma_{-,-} \end{array}], \end{array} \end{array}$$ (35)

which we use to compute conditional expectation and variance of 𝐙 using equation (4). In the case of unmarked agents we assume that the density of agents in $t_{\text{1}}+\Delta t$ conditional on $t_{\text{1}}$ follows a multivariate normal distribution with mean $𝐒E\left[𝐙|𝐔𝐙=𝐛\right]=E\left[{𝐙}_{O}|𝐔𝐙=𝐛\right]+E\left[{𝐙}_{+}|𝐔𝐙=𝐛\right]$ and variance-covariance matrix constructed by $𝐒\text{Var}\left(𝐙|𝐔𝐙=𝐛\right){𝐒}^{T}=\text{Var}({𝐙}_{O}|𝐔𝐙=𝐛)+\text{Var}({𝐙}_{+}|𝐔𝐙=𝐛)$ where $𝐒=[\begin{array}{ccc} {𝐈}_{N} & {𝐈}_{N} & {𝐎}_{N} \end{array}]$. We note that we compute the conditional variances as $\text{var}\left(𝐙|𝐔𝐙=𝐛\right)=𝐀\text{var}\left(𝐙\right){𝐀}^{T}$ with $𝐀={𝐈}_{N}-𝐂𝐔$ and $𝐂=\Sigma {𝐔}^{T}{\left(𝐔\Sigma {𝐔}^{T}\right)}^{-\text{1}}$ (see S1 File). The conditional mean and variance-covariance matrix are then used to compute the likelihood. In the case of marked agents, we use the same approach for the conditional expectation and variance. Here, we assume that $\left({𝐙}_{O},{𝐙}_{+}\right)$ follows a multivariate normal distribution (and not ${𝐙}_{O}+{𝐙}_{+}$) with mean $E[{𝐙}_{O},{𝐙}_{+}|𝐔𝐙=𝐛]$ and variance $\text{Var}\left({𝐙}_{O},{𝐙}_{+}|𝐔𝐙=𝐛\right)$.

### Parameter estimation

For each model in case study one and two, we used a Bayesian approach to estimate the model parameters, where we assumed for each model parameter θ the log-normal prior distribution $\text{log}\theta \sim N\left({\text{0},e}^{\text{2}}\right)$ with e=2. In case study three, we assumed for all parameters, except the scaling of the spatial kernels, log-normal prior distributions $\text{log}\theta \sim N\left({-\text{1}.\text{5},e}^{\text{2}}\right)$ with e=4. For the scaling parameters of the spatial kernels, we assumed log-uniformly distributed priors on the interval [a,b] with a=log(0.1) and b=log(4) according to the dimensions of the sliding window. We used an adaptive Metropolis-Hastings algorithm to sample from the posterior distributions. We ran each of 4 chains from different random initializations for 1000 adaptive iterations and 10000 sampling iterations where we thinned the sampled parameters to every 10-th parameter combination, resulting in 1000 samples per chain. If visual inspection of the trace plots of the chains suggested that the MCMC algorithm was still in a transient phase after 1000 adaptive iterations, we continued the MCMC algorithm for 1000 additional adaptive iterations before conducting the sampling iterations. We evaluated MCMC convergence by calculating the Gelman diagnostic (for results on MCMC convergence, see S1 File).

### Details of case study one

#### Mathematical description of the SSLM.

The model dynamics of the SSLM are defined by the linear Markov operator L as [39]


$$\begin{array}{c} \begin{array}{c} \begin{array}{c} LF(\gamma )=\sum_{x\in \gamma }\left(m+\sum_{y\in \gamma \setminus x}a^{-}(x-y)\right)\left[F(\gamma \setminus x)-F(\gamma )\right]\\ +\sum_{y\in \gamma }\int_{R^{d}}a^{+}(x-y)\left[F(\gamma \cup x)-F(\gamma )\right]dx, \end{array} \end{array} \end{array}$$ (36)

where the first part of the equation models the death events and the second part the birth events. Considering the dynamics of the SSLM in two-dimensional space, the system of differential equations that describes (at the level of first-order perturbation) the time-evolution of population density and two-point cumulant becomes [39]


$$\begin{array}{c} \frac{d}{dt}q_{t}=\;{(A}^{+}-m)q_{t}-A^{-}q_{t}^{\text{2}}, \end{array}$$

$$\begin{array}{c} \begin{array}{c} \frac{d}{dt}p_{t}=\;-\text{2}\pi \int_{\text{0}}^{\infty }k{\tilde{g}}_{t}(k){\tilde{a}}^{-}(k)dk+\left(A^{+}-m\right)p_{t}-\text{2}A^{-}q_{t}p_{t}, \end{array} \end{array}$$ (37)

$$\begin{array}{c} \frac{d}{dt}{\tilde{g}}_{t}(k)=\;\text{2}\left({\tilde{g}}_{t}(k)\left[{\tilde{a}}^{+}(k)-m-\left(A^{-}+{\tilde{a}}^{-}(k)\right)q_{t}\right]+q_{t}\left[{\tilde{a}}^{+}(k)-{\tilde{a}}^{-}(k)q_{t}\right]\right), \end{array}$$

where $\tilde{a}$ denotes the Fourier-transform of the function a. In the S1 File we show the differential equations of the auxiliary model of the SSLM, used to calculate the leading terms of the spatiotemporal cumulants.

#### Model simulations.

We assumed the parameter values $m=\text{1},a^{+}(x)=\text{2}b(x)$ and $a^{-}(x)=b(x)$, where b(x) is the truncated Gaussian kernel


$$\begin{array}{c} \begin{array}{c} b(x)=\left\{ \begin{array}{c} C,\text{ if }|x|\leq \text{3},\\ \text{0},\text{ if }|x|>\text{3}, \end{array}\right.\; \end{array} \end{array}$$ (38)

with $C=\frac{\text{1}}{\text{2}\pi }\int_{|x|\leq \text{3}}\text{exp}\left(-|x{|}^{\text{2}}/\text{2}\right)dx\;$ so that b(x) integrates to one over all space. We furthermore set the spatial scaling parameter to ε=1, and considered the dynamics of the SSLM in the domain $\Omega =[\text{0},\text{8}\text{0}{]}^{\text{2}}$, where we assumed periodic boundary conditions. We simulated the dynamics for t=6 time units, initializing the simulation with a homogeneous Poisson distribution (also called complete spatial randomness) with intensity 1. We discretized the domain into a grid with cell size 1 x 1 and collected from each grid cell data on the number of agents at the end of the simulation.

### Details of case study two

For a mathematical description of the SSLM see Details of case study one and the S1 File.

#### Implementation of the conditional expectations*.*

In case study two we present how we make conditional predictions, i.e., equations (8)–(9), for marked and unmarked individuals in notations that explicitly show the conditioning in terms of the marks of agents. Here we present these conditional predictions in the notation of our framework equation (4) by explicitly defining 𝐔. In the case of unmarked individuals, we do not have data on O,+,− agents separately but rather on counts during the first and second surveys, i.e., on the sums$\;y_{t_{\text{1}},i}=y_{O,i}+y_{-,i}$, and $y_{t_{\text{2}},i}=y_{O,i}+y_{+,i}$. We compute conditional predictions for all grid cells i as


$$\begin{array}{c} \begin{array}{c} E\left[𝐙|{𝐔}^{\left(i\right)}𝐙={𝐛}^{\left(i\right)}\;\forall i\in I\right], \end{array} \end{array}$$ (39)

where


$$\begin{array}{c} {𝐔}^{\left(i\right)}=[\begin{array}{ccc} {𝐈}_{N} & {𝐎}_{N} & {𝐈}_{N}\\ {𝐈}^{F(i-\text{1})} & {𝐈}^{F(i-\text{1})} & {𝐎}^{F(i-\text{1})} \end{array}]. \end{array}$$

Here ${𝐈}_{N}$ and ${𝐎}_{N}$ respectively denote the N-dimensional identity and zero matrix and superscript F(m) means only the first m rows of these matrices are included. Here ${𝐛}^{\left(i\right)}$ is a vector of length N+i−1 where the first N elements are the sums of O and − agents and the last i−1 elements are the sums of O and + agents.

In other words, we assumed that for predicting the transition rates, the researcher would use full information on the agent densities at the first survey, but information on the agent densities at the second survey only for the grid cells preceding the focal cell. The motivation for this is a similar partitioning of the joint likelihood to product of conditionals as presented in equation (5) for the case of the snapshot.

In the case of marked individuals, the first survey yields data on the sum of the O and − agents, but after the second survey it is possible to count the O,+,− agents separately. When making the predictions for the expectations of the densities $Z_{O,i}$, $Z_{+,i}$ and $Z_{-,i}$ for grid cell i, we assume that the cells of index smaller i have already been surveyed twice and hence we condition on their counts of O,+ and − agents. The cells with index larger i have not been surveyed twice and we condition thus on their sum of counts of O and − agents. This yields the conditioning


$$\begin{array}{c} \begin{array}{c} E\left[𝐙|{𝐔}^{\left(i\right)}𝐙={𝐛}^{\left(i\right)}\;\forall i\in I\right], \end{array} \end{array}$$ (40)

where


$$\begin{array}{c} {𝐔}^{\left(i\right)}=[\begin{array}{ccc} {𝐈}^{L(N-i+\text{1})} & {𝐎}^{L(N-i+\text{1})} & {𝐈}^{L(N-i+\text{1})}\\ {𝐈}^{F(i-\text{1})} & {𝐎}^{F(i-\text{1})} & {𝐎}^{F(i-\text{1})}\\ {𝐎}^{F(i-\text{1})} & {𝐈}^{F(i-\text{1})} & {𝐎}^{F(i-\text{1})}\\ {𝐎}^{F(i-\text{1})} & {𝐎}^{F(i-\text{1})} & {𝐈}^{F(i-\text{1})} \end{array}], \end{array}$$

and ${𝐛}^{\left(i\right)}$ is a vector of length N−i+1+3(i−1)=N+2i−2. Note that here the 1,…,N−i+1th element of ${𝐛}^{\left(i\right)}\;$refers to the sum of O and − agents of the first snapshot of grid cells following grid cell i−1 and the N−i+1, …,N+2i−2 th elements to observations of O,+,− agents in grid cells preceding grid cell i. Here 𝐈 and 𝐎 respectively denote the N-dimensional identity and zero matrices, and the superscripts F(m) and L(m) respectively mean that only the first or last m rows of the matrix are included.

#### Likelihood of transition with unknown initial condition*.*

Our framework is based on the leading terms of spatial and spatiotemporal cumulants of RCP models. To compute the leading terms, we either need to know the initial distribution of agents at time $t_{\text{0}}$, e.g., complete spatial randomness, or that the dynamics of the system are in stationary state. If neither of these is possible, we cannot predict the conditional densities in the case of only having a single snapshot observation. However, if we have time-series data we can still model the transition between the two observation and use that information to compute the likelihood. In this case we empirically extract $k^{\left(\text{1}\right)}\;$the one- and $k^{\left(\text{2}\right)}\;$two-point cumulant from the data. Note that from $k^{\left(\text{1}\right)}$ we cannot easily separate the leading terms q and p, and thus for simplicity we set p :=0. From the empirical two-point cumulant we calculate the leading term g as $g(x,y)=\varepsilon^{-d}k^{\left(\text{2}\right)}(\varepsilon^{-\text{1}}x,\;\varepsilon^{-\text{1}}y)$.

### Details of case study three

#### Setup of cancer cell experiments.

Our experimental model comprised time-lapsed observations of drug-sensitive parental non-small lung cancer (NSCLC) PC9 (Sigma-Aldrich, USA) cells stably engineered to express fluorescent label EGFP (VectorBuilder, USA) and drug-resistant cancer cells engineered to express clinically relevant drug-resistant mutation B-RAF-V600E (Addgene, USA) stably expressing fluorescent label mCherry (VectorBuilder, USA). Engineering mutant cells were maintained in RPMI-1640 medium supplemented with 10% heat-inactivated fetal bovine serum and 1% penicillin/streptomycin containing the appropriate concentration of antibiotic selection. Experimentally, 1500 fluorescently labelled cells containing homogenously mixed three initial conditions (drug-resistant cells only, drug-sensitive cells only, co-culture containing different initial ratios of both drug-resistant and drug-sensitive cells) were plated in 96-well microplates (Corning, USA) in 100 µL of culture medium. The experiments were conducted for three technical replicates of each of nine different drug concentrations (0, 0.27, 0.8, 2.5, 7.4, 22.2, 66.7, 200 and 600nM of EGFR-inhibitor Gefitinib (Cayman, USA)). Plating of cells and drug treatment was performed using Microlab liquid handler robot (Hamilton, USA) to ensure consistency across replicates. The cancer cells were seeded into differential initial proportions and time-lapse imaging was acquired using the BioSpa live-cell analysis system (Agilent, USA) every 4 hours for 96 hours to assess cellular dynmacis and evolution under treatment. The resulting images were processed using the open-source software CellProfiler. Briefly, image processing included background subtraction, conversion to 8-bit format, contrast enhancement, and thresholding to isolate individual cells. Then, raw cell counts and coordinates of the two types of cells were extracted for each time point.

#### Mathematical description of the cancer cell model*.*

Note that the cancer cell model comprises agents of types {S,R}. The dynamics of the cancer cell model are defined by the microscopic equation


$$\begin{array}{cc} LF(\gamma )= & \;\sum_{\begin{array}{c} M,N\in \left\{S,R\right\}\\ M\neq N \end{array}}\sum_{x\in \gamma_{M}}\left(m_{M}+\sum_{y\in \gamma_{M}\setminus x}a_{MM}(x-y)+\sum_{z\in \gamma_{N}}a_{MN}(x-z)\right)\left[F\left(\gamma_{M}\setminus x,\gamma_{N}\right)-F\left(\gamma_{M},\;\gamma_{N}\right)\right]\\ & +\sum_{y\in \gamma_{M}}\int_{R^{d}}b_{M}(x-y)\left[F\left(\gamma_{M}\cup x,\gamma_{N}\right)-F\left(\gamma_{M},\gamma_{N}\right)\right]dx, \end{array}$$ (41)

where $\gamma =\left(\gamma_{M},\gamma_{N}\right)$. We apply the mathematical framework of Cornell et al. [37] to derive the system of differential equations for the leading terms of the spatial and spatiotemporal cumulants. The time-evolution of density and two-point cumulant in two-dimensional space are given by


$$\begin{array}{c} \frac{d}{dt}q_{t,S}=-q_{t,S}\left(m_{S}-B_{S}+A_{SS}q_{t,S}+A_{SR}q_{t,R}\right), \end{array}$$

$$\begin{array}{c} \begin{array}{c} \frac{d}{dt}q_{t,R}=-q_{t,R}\left(m_{R}-B_{R}+A_{RR}q_{t,R}+A_{RS}q_{t,S}\right), \end{array} \end{array}$$ (42)

$$\begin{array}{cc} \frac{d}{dt}p_{t,S}= & -\text{2}\pi \int_{\text{0}}^{\infty }k{\tilde{a}}_{SS}(k){\tilde{g}}_{t,S,S}(k)dk+\text{2}\pi \int_{\text{0}}^{\infty }k{\tilde{a}}_{SR}(k){\tilde{g}}_{t,S,R}(k)dk+\left(B_{S}-m_{S}\right)p_{t,S}\\ & -\text{2}A_{SS}p_{t,S}q_{t,S}-A_{SR}\left(p_{t,R}q_{t,S}+p_{t,S}q_{t,R}\right), \end{array}$$ (43)

$$\begin{array}{c} \frac{d}{dt}p_{t,R}=-\text{2}\pi \int_{\text{0}}^{\infty }k{\tilde{a}}_{RR}(k){\tilde{g}}_{t,R,R}(k)dk+\text{2}\pi \int_{\text{0}}^{\infty }k{\tilde{a}}_{RS}(k){\tilde{g}}_{t,R,S}(k)dk+\left(B_{R}-m_{R}\right)p_{t,R}-\text{2}A_{RR}p_{t,R}q_{t,R}-A_{RS}\left(p_{t,S}q_{t,R}+p_{t,R}q_{t,S}\right), \end{array}$$

$$\begin{array}{c} \frac{d}{dt}{\tilde{g}}_{t,S,S}(k)=-\text{2}q_{t,S}\left(-{\tilde{b}}_{S}(k)+{\tilde{a}}_{SR}(k){\tilde{g}}_{t,S,R}(k)+{\tilde{a}}_{SS}(k)q_{t,S}\right)-\text{2}{\tilde{g}}_{t,S,S}(k)\left[m_{S}-{\tilde{b}}_{S}(k)+\left({\tilde{a}}_{SS}(k)+A_{SS}\right)q_{t,S}+{\tilde{a}}_{SR}(k)q_{t,R}\right], \end{array}$$

$$\begin{array}{c} \frac{d}{dt}{\tilde{g}}_{t,R,R}(k)=-\text{2}q_{t,R}\left(-{\tilde{b}}_{R}(k)+{\tilde{a}}_{RS}(k){\tilde{g}}_{t,S,R}(k)+{\tilde{a}}_{RR}(k)q_{t,R}\right)-\text{2}{\tilde{g}}_{t,R,R}(k)\left[m_{R}-{\tilde{b}}_{R}(k)+\left({\tilde{a}}_{RR}(k)+A_{RR}\right)q_{t,R}+{\tilde{a}}_{RS}(k)q_{t,S}\right], \end{array}$$ (44)

$$\begin{array}{cc} \frac{d}{dt}{\tilde{g}}_{t,S,R}(k)= & -{\tilde{a}}_{RS}(k)q_{t,R}\left({\tilde{g}}_{t,S,S}(k)+q_{t,S}\right)-{\tilde{a}}_{SR}(k)q_{t,S}\left({\tilde{g}}_{t,R,R}(k)+q_{t,R}\right)\\ & -{\tilde{g}}_{t,S,R}(k)[m_{S}+m_{R}-{\tilde{b}}_{S}(k)-{\tilde{b}}_{S}(k)+\left({\tilde{a}}_{SS}(k)+A_{SS}+A_{RS}\right)q_{t,S}\\ & +\left({\tilde{a}}_{RR}(k)+A_{RR}+A_{SR}\right)q_{t,R}], \end{array}$$

The differential equations for the auxiliary model is shown in the S1 File.

#### Model fitting.

We fitted one model per drug concentration. Each of the nine drug concentration experiments consists of three initial conditions with three replicates each, i.e., nine datasets (Fig 4a). From those nine datasets, we randomly selected one replicate per initial condition for the test data, and the remaining six datasets were used as training data to fit the model in a Bayesian parameter estimation framework. We denote by D the dataset used in the training, consisting of $T_{d}$ time steps. We calculate the log-likelihood of the data as


$$\begin{array}{c} \text{log}L=\sum_{d=\text{1}}^{D}\sum_{t=\text{1}}^{T_{d}-\text{1}}\sum_{M\in \left\{R,S\right\}}\text{log}L_{t\to t+\text{1}}^{M},\; \end{array}$$ (45)

where $\text{log}L_{t\to t+\text{1}}^{M}$ denotes the log-likelihood of the transition from t to t+1 for agents of type M. To calculate this log-likelihood, we assumed a Poisson distribution for number of new agents appearing, and the Binomial distribution for the number of existing agents surviving from t to t+1. For computational reasons we applied a sliding window approach to compute the conditional expectations in equation (8).

#### Prior & posterior predictive simulations.

For each of the 9 models, we sample 100 parameter vectors from the prior and posterior distributions. For all 9 replicates (6 training & 3 test data) we extract the configurations at the initial time to initialize the posterior predictive simulations from. We use the RCP-simulator of Cornell et al. [37], simulate with each 100 sampled parameter vectors from the true initial conditions of all 9 replicates for 92 hours (92 time steps saved every 4 time step) with different random seed.

## Supporting information

S1 File Section 1–2 include mathematical descriptions of the models used in the case studies. Section 3 provides results on the effect of the ordering of grid cells on the likelihood calculation. Section 4 includes a justification for the conditional variance of agent densities. Section 5 includes results on MCMC convergence, posterior correlations and further validation tests. Section 6 presents a proof of the conditional expectation of agent density.
(PDF)
