# Supplementary material for: R-package agentBayes: Likelihood-based statistical methods for agent-based models
Source: PLoS Comput Biol. 2026 Sep 15;22(9):e1014786. doi: 10.1371/journal.pcbi.1014786 (PMC13619099; doi:10.1371/journal.pcbi.1014786)
Supplement: Supplementary file 1 [file pcbi.1014786.s001.pdf]

Supplementary Information

This Supplementary Information contains:

1. Mathematical description of the auxiliary model of the SSLM used in the second case study
2. Mathematical description of the auxiliary model used in the third case study on cancer cells
3. Partitioning of the joint likelihood
4. Derivation of the conditional variance
5. MCMC convergence analysis, pairwise posterior distributions & validation
6. Justification of equation (4)

1. Mathematical description of the auxiliary model of the SSLM used in the second case study

We derive the dynamics of the auxiliary model of the SSLM. Recall that the auxiliary model consists of agents of three types: surviving agents with mark O, new appearing agents with mark $+$ and dead agents with mark $-$. The dynamics of this model are described by the Markov operator

$$L = L_1 + L_2 + L_3$$

where

$$L_1 F(\gamma) = \sum_{x \in \gamma_O \cup \gamma_+} \int_{\mathbb{R}^d} a^+(x-y)\,[F(\gamma_O, \gamma_+ \cup y, \gamma_-) - F(\gamma)]dy,$$

describes that surviving and newly appearing agents produce and disperse offspring. Further,

$$L_2 F(\gamma) = \sum_{x \in \gamma_O} m\,[F(\gamma_O \setminus x, \gamma_+, \gamma_- \cup x) - F(\gamma)] + \sum_{x \in \gamma_+} m\,[F(\gamma_O, \gamma_+ \setminus x, \gamma_-) - F(\gamma)],$$

where the first summand accounts for surviving agents dying and hence becoming dead agents and the second summand accounts for new agents dying. Finally,

$$\begin{aligned} L_3 F(\gamma) = &\sum_{x \in \gamma_O} \sum_{y \in \gamma_O \setminus x \cup \gamma_+} a^-(x-y)\,[F(\gamma_O \setminus x, \gamma_+, \gamma_- \cup x) - F(\gamma)] \\ &+ \sum_{x \in \gamma_+} \sum_{y \in \gamma_+ \setminus x \cup \gamma_O} a^-(x-y)\,[F(\gamma_O, \gamma_+ \setminus x, \gamma_-) - F(\gamma)], \end{aligned}$$

where the first and second line refer to surviving and new agents dying due to competition with surviving and new agents. We use Mathematica code to derive the differential equations of the leading terms of the cumulants. For the mean-field density we get

$$\frac{d}{dt} q_{t,O} = -q_{t,O} d_q$$

$$\frac{d}{dt} q_{t,+} = b_q - q_{t,+} d_q$$

$$\frac{d}{dt} q_{t,-} = q_{t,O} d_q,$$

with the death rate $d_q = m + A^-(q_{t,O} + q_{t,+})$ and the birth rate $b_q = A^+(q_{t,O} + q_{t,+})$. Further,

$$\frac{d}{dt} p_{t,O} = -2\pi \int_0^\infty k \tilde{a}^-(k)\big[\tilde{g}_{t,O,O}(k) + \tilde{g}_{t,O,+}(k)\big]dk - \big(p_{t,O} d_q + q_{t,O} d_p\big),$$

$$\frac{d}{dt} p_{t,+} = -2\pi \int_0^\infty k \tilde{a}^-(k)\big[\tilde{g}_{t,O,+}(k) + \tilde{g}_{t,+,+}(k)\big]dk - \big(p_{t,+} d_q + q_{t,+} d_p\big) + b_p,$$

$$\frac{d}{dt} p_{t,-} = 2\pi \int_0^\infty k \tilde{a}^-(k)\big[\tilde{g}_{t,O,O}(k) + \tilde{g}_{t,O,+}(k)\big]dk + p_{t,O} d_q + q_{t,O} d_p,$$

with the correction to the death rate $d_p = A^-(p_{t,O} + p_{t,+})$ and the correction to the birth rate $b_p = A^+(p_{t,O} + p_{t,+})$. Finally,

$$\frac{d}{dt}\tilde{g}_{t,O,O}(k) = -2\left(\tilde{a}^-(k)q_{t,O}\left[\tilde{g}_{t,O,+}(k) + q_{t,O}\right] + \tilde{g}_{t,O,O}(k)\left[\tilde{a}^-(k)q_{t,O} + d_q\right]\right),$$

$$\begin{aligned}\frac{d}{dt}\tilde{g}_{t,O,+}(k) = {} & \tilde{a}^+(k)\left[\tilde{g}_{t,O,O}(k) + \tilde{g}_{t,O,+}(k) + q_{t,O}\right] \\ & - \tilde{a}^-(k)\left[\tilde{g}_{t,+,+}(k)q_{t,O} + \left(\tilde{g}_{t,O,O}(k) + 2q_{t,O}\right)q_{t,+}\right] - \tilde{g}_{t,O,+}(k)\left(\tilde{a}^-(k)\left[q_{t,O} + q_{t,+}\right]\right) \\ & + 2d_q,\end{aligned}$$

$$\frac{d}{dt}\tilde{g}_{t,O,-}(k) = \tilde{a}^-(k)q_{t,O}\left[\tilde{g}_{t,O,+}(k) - \tilde{g}_{t,+,-}(k) + q_{t,O}\right] + \left[\tilde{g}_{t,O,O}(k) - \tilde{g}_{t,O,-}(k)\right]\left[\tilde{a}^-(k)q_{t,O} + d_q\right],$$

$$\begin{aligned}\frac{d}{dt}\tilde{g}_{t,+,+}(k) = {} & -2\left(\tilde{a}^-(k)q_{t,O}\left[\tilde{g}_{t,O,+}(k) + q_{t,O}\right] - \tilde{a}^+(k)\left[\tilde{g}_{t,O,+}(k) + \tilde{g}_{t,+,+}(k) + q_{t,O}\right]\right. \\ & \left. + \tilde{g}_{t,+,+}(k)\left[\tilde{a}^-(k)q_{t,+} + d_q\right]\right),\end{aligned}$$

$$\begin{aligned}\frac{d}{dt}\tilde{g}_{t,+,-}(k) = {} & d_q\left[\tilde{g}_{t,O,+}(k) - \tilde{g}_{t,+,-}(k)\right] + \tilde{a}^+(k)\left[\tilde{g}_{t,O,-}(k) - \tilde{g}_{t,+,-}(k)\right] \\ & + \tilde{a}^-(k)\left[q_{t,O}\left(\tilde{g}_{t,O,+}(k) + \tilde{g}_{t,+,+}(k) + q_{t,+}\right) - q_{t,+}\left(\tilde{g}_{t,O,-}(k) + \tilde{g}_{t,+,-}(k)\right)\right],\end{aligned}$$

$$\frac{d}{dt}\tilde{g}_{t,-,-}(k) = 2\left(\tilde{a}^-(k)q_{t,O}\tilde{g}_{t,+,-}(k) + \tilde{g}_{t,O,-}(k)\left[\tilde{a}^-(k)q_{t,O} + d_q\right]\right).$$

2. Mathematical description of the auxiliary model used in the third case study on cancer cells

For the auxiliary model of the cancer cells, we denote the configurations $\gamma = (\gamma_S, \gamma_R) = \left(\gamma_{S,O}, \gamma_{S,+}, \gamma_{S,-}, \gamma_{R,O}, \gamma_{R,+}, \gamma_{R,-}\right)$ where the first subscript refers to the cell type and the second subscript to the auxiliary type. The dynamics of the auxiliary model are described by the Markov operator

$$L = L_1 + L_2 + L_3,$$

where

$$L_1F(\gamma) = \sum_{\substack{M,N\in\{S,R\} \\ M\neq N}} \sum_{x\in\gamma_{M,O}\cup\gamma_{M,+}} \int_{\mathbb{R}^d} b_M(x-y)\left[F\left(\gamma_{M,O}, \gamma_{M,+}\cup x, \gamma_{M,-}, \gamma_N\right) - F(\gamma)\right]dy$$

describes birth events of both types of cells. Next,

$$\begin{aligned}L_2F(\gamma) = {} & \sum_{M\in\{S,R\}} \sum_{x\in\gamma_{M,O}} m\left[F\left(\gamma_{M,O}\setminus x, \gamma_{M,+}, \gamma_{M,-}\cup x\right) - F(\gamma)\right] \\ & + \sum_{x\in\gamma_{M,+}} m\left[F\left(\gamma_{M,O}, \gamma_{M,+}\setminus x, \gamma_{M,-}\right) - F(\gamma)\right],\end{aligned}$$

which describes the density-independent mortality. Finally,

$$L_3F(\gamma) = \sum_{\substack{M,N\in\{S,R\} \\ M\neq N}} \sum_{x\in\gamma_{M,O}} \left(\sum_{y\in\gamma_{M,O}\setminus x\cup\gamma_{M,+}} a_{MM}(x-y) + \sum_{z\in\gamma_{N,O}\cup\gamma_{N,+}} a_{MN}(x-z)\right)$$

$$
\begin{aligned}
&[F(\gamma_{M,O} \setminus x, \gamma_{M,+}, \gamma_{M,-} \cup x, \gamma_N) - F(\gamma_M, \gamma_N)] \\
&\qquad + \sum_{x \in \gamma_{M,+}} \left(\sum_{y \in \gamma_{M,O} \setminus x \cup \gamma_{M,+}} a_{MM}(x-y) + \sum_{z \in \gamma_{N,O} \cup \gamma_{N,+}} a_{MN}(x-z) \right) \\
&\qquad [F(\gamma_{M,O}, \gamma_{M,+} \setminus x, \gamma_{M,-}, \gamma_N) - F(\gamma_M, \gamma_N)],
\end{aligned}
$$

which accounts for surviving agents dying (and becoming dead agents) and new agents dying (and disappearing) by intra- and interspecific interactions. We derive the differential equations for the leading terms of the cumulants for this model. For agents of type $M \in \{S, R\}$, the evolution of the mean-field densities is

$$
\frac{d}{dt} q_{t,M,O} = -q_{t,M,O} d_{q,M}
$$

$$
\frac{d}{dt} q_{t,M,+} = B_M (q_{t,M,O} + q_{t,M,+}) - q_{t,M,+} d_{q,M},
$$

$$
\frac{d}{dt} q_{t,M,-} = q_{t,M,-} d_{q,M},
$$

with the death term $d_{q,M} = m_M + A_{MM}(q_{t,M,O} + q_{t,M,+}) + A_{MN}(q_{t,N,O} + q_{t,N,+})$ consisting of density-independent death, and intra- & interspecific interaction. Next, the evolution of the first-order correction to the densities is given by

$$
\begin{aligned}
\frac{d}{dt} p_{t,M,O} = &-2\pi \int_0^\infty k \tilde{a}_{MM}(k) [\tilde{g}_{t,M,O,M,O}(k) + \tilde{g}_{t,M,O,M,+}(k)] \\
&- 2\pi \int_0^\infty k \tilde{a}_{MN}(k) [\tilde{g}_{t,M,O,N,O}(k) + \tilde{g}_{t,M,O,N,+}(k)] - d_{M,p} ,
\end{aligned}
$$

with the correction to the death term

$$
\begin{aligned}
d_{M,p} = m_M p_{t,M,O} &+ A_{MM}[(p_{t,M,+} q_{t,M,O} + p_{t,M,O} q_{t,M,+}) + 2 p_{t,M,O} q_{t,M,O}] \\
&+ A_{MN}[(p_{t,N,O} q_{t,M,O} + p_{t,M,O} q_{t,N,O}) + (p_{t,N,+} q_{t,M,O} + p_{t,M,O} q_{t,N,+})],
\end{aligned}
$$

Finally, recall that $M \in \{S, R\}$ and let $N \in \{S, R\}$ with $N \neq M$, then the evolution of the leading term of the two-point cumulants is

$$
\begin{aligned}
&\frac{d}{dt} \tilde{g}_{t,M,O,M,O}(k) \\
&\qquad = -2 \Big(\tilde{g}_{t,M,O,M,O}(k) [d_{q,M} + \tilde{a}_{MM}(k) q_{t,M,O}] \\
&\qquad + q_{t,M,O} \Big[\tilde{a}_{MN}(k) [\tilde{g}_{t,M,O,N,O}(k) + \tilde{g}_{t,M,O,N,+}(k)] \\
&\qquad + \tilde{a}_{MM}(k) [\tilde{g}_{t,M,O,M,+}(k) + q_{t,M,O}] \Big] \Big),
\end{aligned}
$$

$$\frac{d}{dt}\tilde{g}_{t,M,O,M,+}(k)$$
$$= -2d_{q,M}\tilde{g}_{t,M,O,M,+}(k)$$
$$- q_{t,M,O}\big(\tilde{a}_{MM}(k)\big[\tilde{g}_{t,M,O,M,+}(k) + \tilde{g}_{t,M,+,M,+}(k)\big]$$
$$+ \tilde{a}_{MN}(k)\big[\tilde{g}_{t,M,+,N,O}(k) + \tilde{g}_{t,M,+,N,+}(k)\big]\big)$$
$$+ \tilde{b}_M(k)\big[\tilde{g}_{t,M,O,M,O}(k) + \tilde{g}_{t,M,O,M,+}(k) + q_{t,M,O}\big]$$
$$- q_{t,M,+}\big(\tilde{a}_{MM}(k)\big[\tilde{g}_{t,M,O,M,O}(k) + \tilde{g}_{t,M,O,M,+}(k)\big]$$
$$+ \tilde{a}_{MN}(k)\big[\tilde{g}_{t,M,O,N,O}(k) + \tilde{g}_{t,M,O,N,+}(k)\big]\big) - 2\tilde{a}_{MM}(k)q_{t,M,O}q_{t,M,+},$$

$$\frac{d}{dt}\tilde{g}_{t,M,O,M,-}(k)$$
$$= d_{q,M}\left(\tilde{g}_{t,M,O,M,O}(k) + \tilde{g}_{t,M,O,M,-}(k)\right)$$
$$+ q_{t,M,O}\big(\tilde{a}_{MM}(k)\big[\tilde{g}_{t,M,O,M,O}(k) + \tilde{g}_{t,M,O,M,+}(k) - \tilde{g}_{t,M,O,M,-}(k)$$
$$- \tilde{g}_{t,M,+,M,-}(k)\big]$$
$$+ \tilde{a}_{MN}(k)\big[\tilde{g}_{t,M,O,N,O}(k) + \tilde{g}_{t,M,O,N,+}(k) - \tilde{g}_{t,M,-,N,O}(k) - \tilde{g}_{t,M,-,N,+}(k)\big]\big)$$
$$+ \tilde{a}_{MM}(k)q_{t,M,O}^2,$$

$$\frac{d}{dt}\tilde{g}_{t,M,+,M,+}(k)$$
$$= 2\tilde{b}_M(k)\big(\tilde{g}_{t,M,O,M,+}(k) + \tilde{g}_{t,M,+,M,+}(k) + q_{t,M,+}\big)$$
$$- 2\tilde{g}_{t,M,+,M,+}(k)\big(d_{q,M} + \tilde{a}_{MM}(k)q_{t,M,+}\big)$$
$$- 2q_{t,M,+}\big(\tilde{a}_{MN}(k)\big[\tilde{g}_{t,M,+,N,O}(k) + \tilde{g}_{t,M,+,N,+}(k)\big]$$
$$+ \tilde{a}_{MM}(k)\big[\tilde{g}_{t,M,O,M,+}(k) + q_{t,M,+}\big]\big),$$

$$\frac{d}{dt}\tilde{g}_{t,M,+,M,-}(k)$$
$$= \tilde{b}_M(k)\left(\tilde{g}_{t,M,O,M,-}(k) + \tilde{g}_{t,M,+,M,-}(k)\right)$$
$$+ d_{q,M}\left(\tilde{g}_{t,M,O,M,+}(k) - \tilde{g}_{t,M,+,M,-}(k)\right)$$
$$+ q_{t,M,O}\big(\tilde{a}_{MM}(k)\big[\tilde{g}_{t,M,O,M,+}(k) + \tilde{g}_{t,M,+,M,+}(k)\big]$$
$$+ \tilde{a}_{MN}(k)\big[\tilde{g}_{t,M,+,N,O}(k) + \tilde{g}_{t,M,+,N,+}(k)\big]\big)$$
$$- q_{t,M,+}\big(\tilde{a}_{MM}(k)\big[\tilde{g}_{t,M,O,M,-}(k) + \tilde{g}_{t,M,+,M,-}(k)\big]$$
$$+ \tilde{a}_{MN}(k)\big[\tilde{g}_{t,M,-,N,O}(k) + \tilde{g}_{t,M,-,N,+}(k)\big]\big) + \tilde{a}_{MM}(k)q_{t,M,O}q_{t,M,+},$$

$$\frac{d}{dt}\tilde{g}_{t,M,-,M,-}(k)$$
$$= 2d_{q,M}\tilde{g}_{t,M,O,M,-}(k)$$
$$+ 2q_{t,M,O}\big(\tilde{a}_{MM}(k)\big[\tilde{g}_{t,M,O,M,-}(k) + \tilde{g}_{t,M,+,M,-}(k)\big]$$
$$+ \tilde{a}_{MN}(k)\big[\tilde{g}_{t,M,-,N,O}(k) + \tilde{g}_{t,M,-,N,+}(k)\big]\big),$$

$$\frac{d}{dt}\tilde{g}_{t,M,O,N,O}(k)$$
$$= -q_{t,M,O}\big(\tilde{a}_{MM}(k)\tilde{g}_{t,M,+,N,O}(k) + \tilde{a}_{MN}(k)\big[\tilde{g}_{t,N,O,N,O}(k) + \tilde{g}_{t,N,O,N,+}(k)\big]\big)$$
$$- q_{t,N,O}\big(\tilde{a}_{NN}(k)\tilde{g}_{t,M,O,N,+}(k) + \tilde{a}_{NM}(k)\big[\tilde{g}_{t,M,O,M,O}(k) + \tilde{g}_{t,M,O,M,+}(k)\big]\big)$$
$$- q_{t,M,O}q_{t,N,O}\big(\tilde{a}_{MN}(k) + \tilde{a}_{NM}(k)\big)$$
$$- \tilde{g}_{t,M,O,N,O}(k)\big[d_{q,M} + d_{q,N} + \tilde{a}_{MM}(k) + \tilde{a}_{NN}(k)\big],$$

$$\frac{d}{dt}\tilde{g}_{t,M,O,N,+}(k)$$
$$= \tilde{b}_N(k)\left(\tilde{g}_{t,M,O,N,O}(k) + \tilde{g}_{t,M,O,N,+}(k)\right)$$
$$- q_{t,M,O}\big(\tilde{a}_{MM}(k)\tilde{g}_{t,M,+,N,+}(k) + \tilde{a}_{MN}(k)\big[\tilde{g}_{t,N,O,N,+}(k) + \tilde{g}_{t,N,+,N,+}(k)\big]\big)$$
$$- q_{t,N,+}\big(\tilde{a}_{NN}(k)\tilde{g}_{t,M,O,N,+}(k) + \tilde{a}_{NM}(k)\big[\tilde{g}_{t,M,O,M,O}(k) + \tilde{g}_{t,M,O,M,+}(k)\big]\big)$$
$$- q_{t,M,O}q_{t,N,+}\big(\tilde{a}_{MN}(k) + \tilde{a}_{NM}(k)\big)$$
$$- \tilde{g}_{t,M,O,N,+}(k)\big(d_{q,M} + d_{q,N} + \tilde{a}_{MM}(k)q_{t,M,O} + \tilde{a}_{NN}(k)q_{t,N,+}\big),$$

$$\frac{d}{dt}\tilde{g}_{t,M,O,N,-}(k)$$
$$= d_{q,N}\tilde{g}_{t,M,O,N,O}(k) - d_{q,M}\tilde{g}_{t,M,O,N,-}(k)$$
$$- q_{t,M,O}\big(\tilde{a}_{MM}(k)\big[\tilde{g}_{t,M,O,N,-}(k) + \tilde{g}_{t,M,+,N,-}(k)\big]$$
$$+ \tilde{a}_{MN}(k)\big[\tilde{g}_{t,N,O,N,-}(k) + \tilde{g}_{t,N,+,N,-}(k)\big]\big)$$
$$+ q_{t,N,O}\big(\tilde{a}_{NN}(k)\big[\tilde{g}_{t,M,O,N,O}(k) + \tilde{g}_{t,M,O,N,+}(k)\big]$$
$$+ \tilde{a}_{NM}(k)\big[\tilde{g}_{t,M,O,M,O}(k) + \tilde{g}_{t,M,O,M,+}(k)\big]\big) + q_{t,M,O}q_{t,N,O}\tilde{a}_{NM}(k),$$

$$\frac{d}{dt}\tilde{g}_{t,M,+,N,+}(k)$$
$$= \tilde{b}_N(k)\left(\tilde{g}_{t,M,+,N,O}(k) + \tilde{g}_{t,M,+,N,+}(k)\right)$$
$$+ \tilde{b}_M(k)\left(\tilde{g}_{t,M,O,N,+}(k) + \tilde{g}_{t,M,+,N,+}(k)\right) - \left(d_{q,M} + d_{q,N}\right)\tilde{g}_{t,M,+,N,+}(k)$$
$$- q_{t,M,+}\big(\tilde{a}_{MM}(k)\big[\tilde{g}_{t,M,O,N,+}(k) + \tilde{g}_{t,M,+,N,+}(k)\big]$$
$$+ \tilde{a}_{MN}(k)\big[\tilde{g}_{t,N,O,N,+}(k) + \tilde{g}_{t,N,+,N,+}(k)\big]\big)$$
$$- q_{t,N,+}\big(\tilde{a}_{NN}(k)\big[\tilde{g}_{t,M,+,N,O}(k) + \tilde{g}_{t,M,+,N,+}(k)\big]$$
$$+ \tilde{a}_{NM}(k)\big[\tilde{g}_{t,M,O,M,+}(k) + \tilde{g}_{t,M,+,M,+}(k)\big]\big) - q_{t,M,+}q_{t,N,+}\big(\tilde{a}_{MN}(k) + \tilde{a}_{NM}(k)\big),$$

$$\frac{d}{dt}\tilde{g}_{t,M,+,N,-}(k)$$
$$= d_{q,N}\tilde{g}_{t,M,+,N,O}(k) - d_{q,M}\tilde{g}_{t,M,+,N,-}(k)$$
$$+ \tilde{b}_M(k)\left(\tilde{g}_{t,M,O,N,-}(k) + \tilde{g}_{t,M,+,N,-}(k)\right)$$
$$- q_{t,M,+}\big(\tilde{a}_{MM}(k)\big[\tilde{g}_{t,M,O,N,-}(k) + \tilde{g}_{t,M,+,N,-}(k)\big]$$
$$+ \tilde{a}_{MN}(k)\big[\tilde{g}_{t,N,O,N,-}(k) + \tilde{g}_{t,N,+,N,-}(k)\big]\big)$$
$$+ q_{t,N,O}\big(\tilde{a}_{NN}(k)\big[\tilde{g}_{t,M,+,N,O}(k) + \tilde{g}_{t,M,+,N,+}(k)\big]$$
$$+ \tilde{a}_{NM}(k)\big[\tilde{g}_{t,M,O,M,+}(k) + \tilde{g}_{t,M,+,M,+}(k)\big]\big) + q_{t,M,+}q_{t,N,+}\tilde{a}_{NM}(k),$$

$$\frac{d}{dt}\tilde{g}_{t,M,-,N,-}(k)$$
$$= d_{q,M}\tilde{g}_{t,M,O,N,-}(k) + d_{q,N}\tilde{g}_{t,M,-,N,O}(k)$$
$$+ q_{t,M,O}\big(\tilde{a}_{MM}(k)\big[\tilde{g}_{t,M,O,N,-}(k) + \tilde{g}_{t,M,+,N,-}(k)\big]$$
$$+ \tilde{a}_{MN}(k)\big[\tilde{g}_{t,N,O,N,-}(k) + \tilde{g}_{t,N,+,N,-}(k)\big]\big)$$
$$+ q_{t,N,O}\big(\tilde{a}_{NN}(k)\big[\tilde{g}_{t,M,-,N,O}(k) + \tilde{g}_{t,M,-,N,+}(k)\big]$$
$$+ \tilde{a}_{NM}(k)\big[\tilde{g}_{t,M,O,M,-}(k) + \tilde{g}_{t,M,+,M,-}(k)\big]\big).$$

3. Partitioning of the joint likelihood

The ordering of the grid cells may numerically affect the results of the likelihood calculations with Eq. 1-3. Here we tested and show in Fig A the log-likelihood profiles of Eq. 5-7 using a fixed ordering of the grid cells (black line) and with three random orderings (coloured). While the absolute value of the likelihood varies between the orderings, the likelihood profile remains stable.

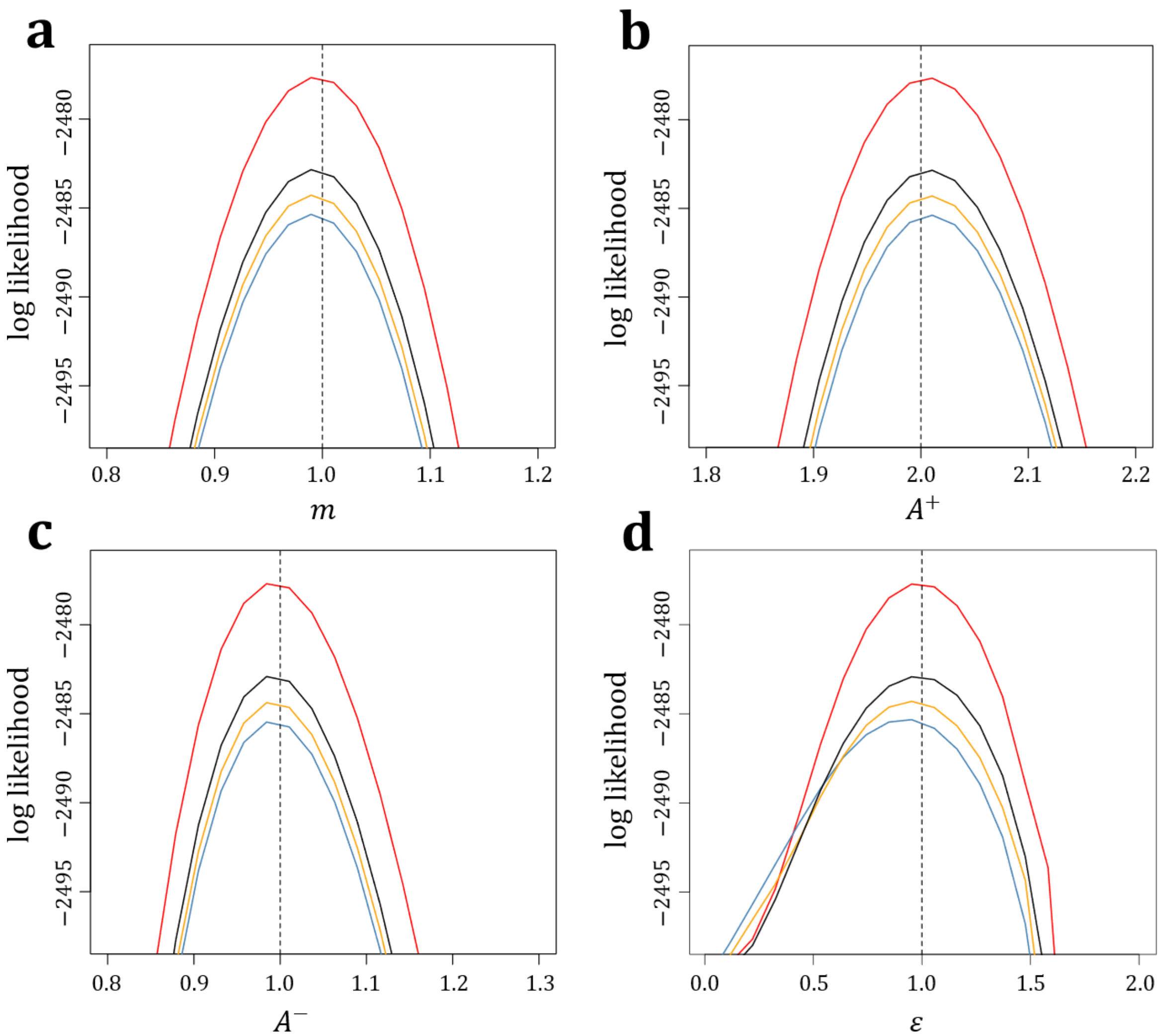

Fig A: Log-likelihood profiles of the model parameters. *We use a fixed ordering of the grid cells in Eq. 5-7 (black) and three random orderings (coloured). Profiles are calculated varying one model parameter and using true parameter values for all other model parameters.* ***a,*** *Profile for density-independent mortality m.* ***b,*** *Profile for reproduction and dispersal rate A^+.* ***c,*** *Profile for competition rate A^-.* ***d,*** *Profile for scaling of interaction kernels ε.*

4. Derivation of the conditional variance

Let $\mathbf{Z} = (Z_1, \dots, Z_n)^\mathrm{T}$ be a multivariate random variable with $E[\mathbf{Z}] = \boldsymbol{\mu}$ and the variance-covariance matrix $\boldsymbol{\Sigma}$. For any $k \times n$ dimensional matrix $\mathbf{U}$ of rank k and vector $\mathbf{b}$ of length k let $\mathbf{UZ} = \mathbf{b}$, $\mathbf{A} = \mathbf{I}_n - \mathbf{CU}$ such that $\boldsymbol{Z} = \mathbf{AZ} + \mathbf{CUZ}$ where $\mathbf{C} = \boldsymbol{\Sigma}\mathbf{U}^T(\mathbf{U}\boldsymbol{\Sigma}\mathbf{U}^T)^{-1}$. Then,

$$\mathrm{Var}(\mathbf{Z}|\mathbf{UZ} = \mathbf{b}) = \mathrm{Var}(\mathbf{AZ} + \mathbf{CUZ}|\mathbf{UZ} = \mathbf{b})$$

$$= \mathrm{Var}(\mathbf{AZ}|\mathbf{UZ} = \mathbf{b}) + \mathrm{Var}(\mathbf{CUZ}|\mathbf{UZ} = \mathbf{b}) + \mathrm{Cov}(\mathbf{AZ}, \mathbf{CUZ}) + \mathrm{Cov}(\mathbf{CUZ}, \mathbf{AZ})$$

$$= \mathrm{Var}(\mathbf{AZ}|\mathbf{UZ} = \mathbf{b}) + \mathrm{Var}(\mathbf{CUZ}|\mathbf{UZ} = \mathbf{b}) + \mathbf{C}\mathrm{Cov}(\mathbf{AZ}, \mathbf{UZ}) + \mathrm{Cov}(\mathbf{UZ}, \mathbf{AZ})\mathbf{C}^T$$

Since from $\mathrm{Cov}(\mathbf{AZ}, \mathbf{UZ}) = 0$, if $\mathbf{AZ}$ and $\mathbf{UZ}$ would be independent (as explained in *Methods*) and $\mathrm{Var}(\mathbf{CUZ}|\mathbf{UZ} = \mathbf{b}) = \mathbf{C}\mathrm{Var}(\mathbf{UZ}|\mathbf{UZ} = \mathbf{b})\mathbf{C}^T = 0$, the expression simplifies to

$$\mathrm{Var}(\mathbf{Z}|\mathbf{UZ} = \mathbf{b}) = \mathrm{Var}(\mathbf{AZ}|\mathbf{UZ} = \mathbf{b}) = \mathbf{A}\mathrm{Var}(\mathbf{Z})\mathbf{A}^T,$$

where $\mathrm{Var}(\mathbf{z}) = \mathrm{diag}(\boldsymbol{\Sigma})$.

5. MCMC convergence analysis, pairwise posterior distributions & validation

For the inference tasks in the three case studies, we assessed the MCMC convergence by computing the mean and upper confidence intervals of the potential scale reduction factor. The results are presented in Table A-C and indicate overall convergence. For case study one and two, we additionally assessed parameter correlations by visualizing the pairwise posterior distributions for all models. The results are presented in Fig B-J and indicate correlations among few parameters, which become less prevalent when moving from spatial snapshot to time-series data. For case study three, we conducted an additional validation test by performing inference on data generated by one of the calibrated models. The results are presented in Fig K. A comparison between the true values used to simulate the data and the estimated posterior distribution generally suggests the validity of the estimation scheme. As an exception, one of the spatial length scale parameters (parameter $l_{b_s}^{-1}$) remains underestimated. This may be either due to this parameterization being based on a single replicate simulation, or the fact that the perturbation framework is asymptotically exact in the limit of large interaction scales ($l^{-1} \to 0$) and therefore constitutes an approximation for finite interaction scales. For this additional validation, we assessed MCMC convergence using the mean potential scale reduction factor and inspected chain mixing through trace plots. The results are presented in Fig L and indicate satisfactory mixing and convergence.

***Table A: MCMC convergence analysis for case study 1**. For each non-spatial and spatial model, 4 chains were run for 11,000 iterations, of which we discarded the first 1000 iterations during which adaptation was applied, and thinned the remaining by 10 to obtain 1000 samples per chain. The mean and upper confidence interval (in brackets) for potential scale reduction factor are reported.*

	Density-independent death rate m	Fecundity rate A^{+}	Death by competition rate A^{-}	Length scale of interaction ε
Non-spatial	1.32 (1.78)	1.02 (1.07)	1.42 (1.97)	1.00 (1.00)
Spatial Normal	1.02 (1.05)	1.02 (1.05)	1.02 (1.04)	1.00 (1.01)
Spatial Poisson	1.01 (1.01)	1.01 (1.01)	1.02 (1.02)	1.01 (1.02)

***Table B: MCMC convergence analysis for case study 2**. For each non-spatial and spatial model, 4 chains were run for 11,000 iterations, of which we discarded the first 1000 iterations during which adaptation was applied, and thinned the remaining by 10 to obtain 1000 samples per chain. The mean and upper confidence interval (in brackets) for potential scale reduction factor are reported.*

**a) Unmarked Agents**				
	Density-independent death rate m	Fecundity rate A^{+}	Death by competition rate A^{-}	Length scale of interaction ε
Non-spatial	1.00 (1.01)	1.01 (1.02)	1.04 (1.09)	1.01 (1.01)
Spatial Normal	1.04 (1.10)	1.00 (1.00)	1.03 (1.08)	1.03 (1.08)
Spatial Poisson	1.01 (1.02)	1.03 (1.06)	1.01 (1.03)	1.02 (1.06)
**b) Marked Agents**				
	Density-independent death rate m	Fecundity rate A^{+}	Death by competition rate A^{-}	Length scale of interaction ε
Non-spatial	1.06 (1.15)	1.00 (1.00)	1.12 (1.30)	1.02 (1.07)
Spatial Normal	1.03 (1.06)	1.00 (1.00)	1.04 (1.11)	1.02 (1.06)
Spatial Poisson	1.06 (1.17)	1.00 (1.01)	1.10 (1.27)	1.03 (1.08)

***Table C: MCMC convergence analysis for case study 3**. For each of the 9 models, 4 chains were run for 11,000 iterations, of which we discarded the first 1000 iterations during which adaptation was applied, and thinned the remaining by 10 to obtain 1000 samples per chain. If visual inspection of the trace plots suggested that the chains were*

still in a transient phase, we continued the MCMC algorithm for additional 1000 adaptive iterations and additional 10,000 iterations which thinned by 10 obtained 1000 samples. The mean and upper confidence interval (in brackets) for potential scale reduction factor are reported. Asterisks indicate that one (*) or two (**) chains were discarded due to non-convergence.

	0.0	0.27*	0.8*	2.5	7.4	22.2	66.7**	200.0*	600.0
Fecundity of sensitive cells B_S	1.00 (1.00)	1.00 (1.01)	1.00 (1.00)	1.00 (1.00)	1.00 (1.00)	1.00 (1.00)	0.99 (0.99)	0.99 (1.00)	1.00 (1.01)
Fecundity of resistant cells B_R	1.00 (1.00)	1.00 (1.01)	1.00 (1.02)	0.99 (1.00)	1.01 (1.04)	1.00 (1.01)	0.99 (1.00)	1.00 (1.00)	1.00 (1.01)
Death by competition rate A_{SS}	1.00 (1.01)	1.00 (1.00)	1.03 (1.06)	1.00 (1.00)	1.00 (1.01)	1.00 (1.01)	1.00 (1.02)	1.00 (1.01)	1.00 (1.00)
Death by competition rate A_{SR}	1.00 (1.00)	1.00 (1.01)	1.00 (1.00)	1.00 (1.00)	1.00 (1.01)	1.00 (1.00)	1.00 (1.01)	1.00 (1.00)	1.00 (1.00)
Death by competition rate A_{RS}	1.00 (1.00)	1.00 (1.00)	1.00 (1.01)	1.00 (1.01)	1.07 (1.17)	1.00 (1.01)	0.99 (0.99)	0.99 (1.00)	1.00 (1.01)
Death by competition rate A_{RR}	1.04 (1.10)	1.00 (1.01)	1.01 (1.03)	0.99 (1.00)	1.07 (1.17)	1.00 (1.01)	1.00 (1.01)	1.00 (1.00)	1.00 (1.00)
Density-independent mortality m_S	1.00 (1.00)	1.00 (1.01)	1.01 (1.03)	1.00 (1.00)	1.00 (1.00)	1.00 (1.00)	1.00 (1.00)	1.00 (1.01)	1.00 (1.00)
Density-independent mortality m_R	1.00 (1.01)	1.00 (1.01)	1.00 (1.02)	0.99 (1.00)	1.03 (1.09)	1.00 (1.01)	1.00 (1.00)	1.00 (1.00)	1.00 (1.01)
Length scale of dispersal l_S	1.00 (1.01)	1.00 (1.00)	1.00 (1.01)	1.00 (1.00)	1.00 (1.00)	1.00 (1.01)	1.00 (1.00)	1.00 (1.00)	1.00 (1.01)
Length scale of dispersal l_R	1.04 (1.11)	1.00 (1.00)	1.01 (1.02)	1.00 (1.00)	1.05 (1.13)	1.00 (1.01)	1.00 (1.01)	1.03 (1.04)	1.00 (1.01)
Length scale of interaction l_A	1.00 (1.00)	1.00 (1.00)	1.01 (1.01)	1.01 (1.02)	1.06 (1.16)	1.00 (1.01)	1.01 (1.04)	1.01 (1.03)	1.00 (1.00)

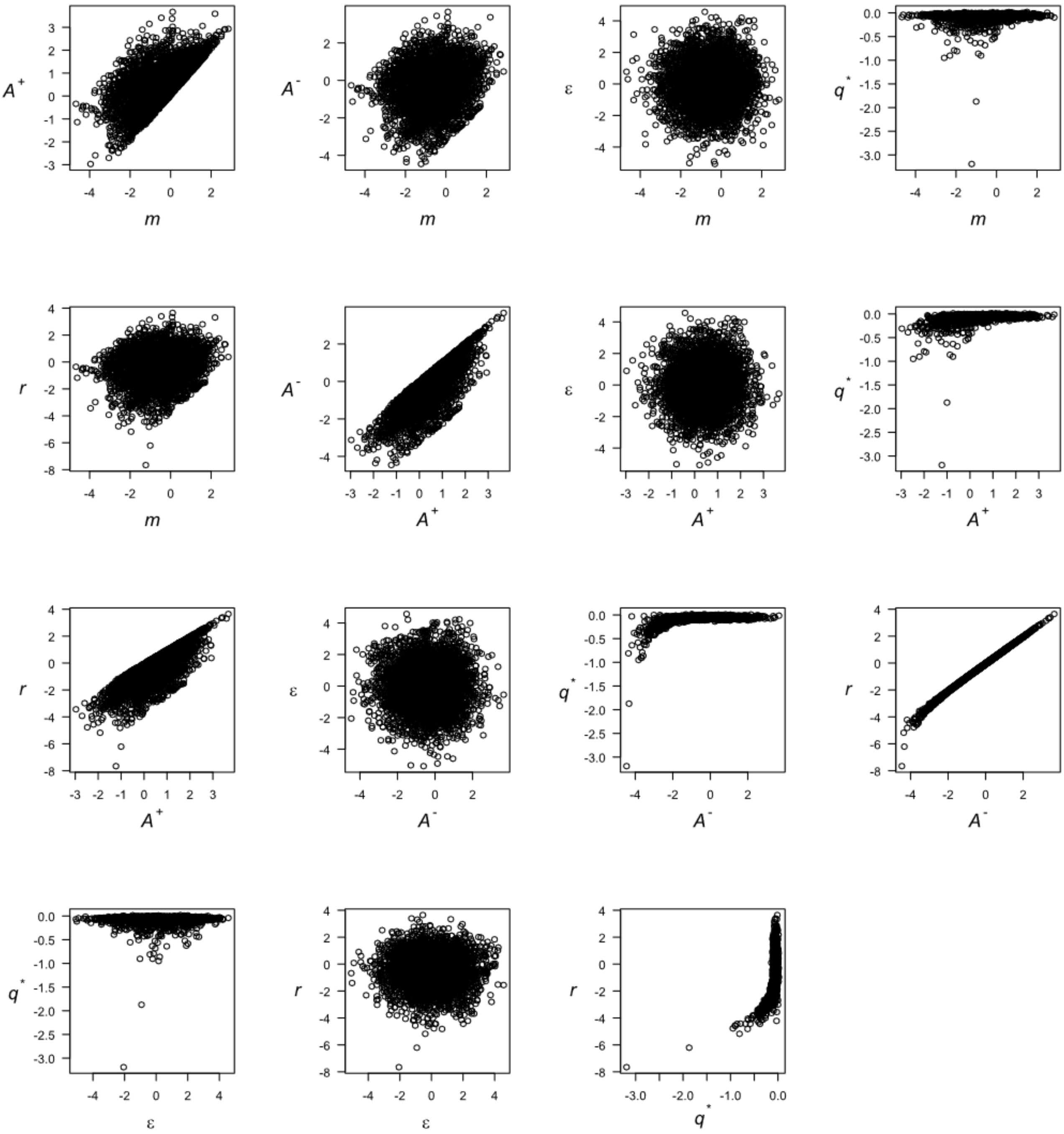

Fig B: Pairwise posterior distributions for non-spatial model in case study one. *We plot the pairwise posterior distributions of all raw and derived parameter pairwise combinations.*

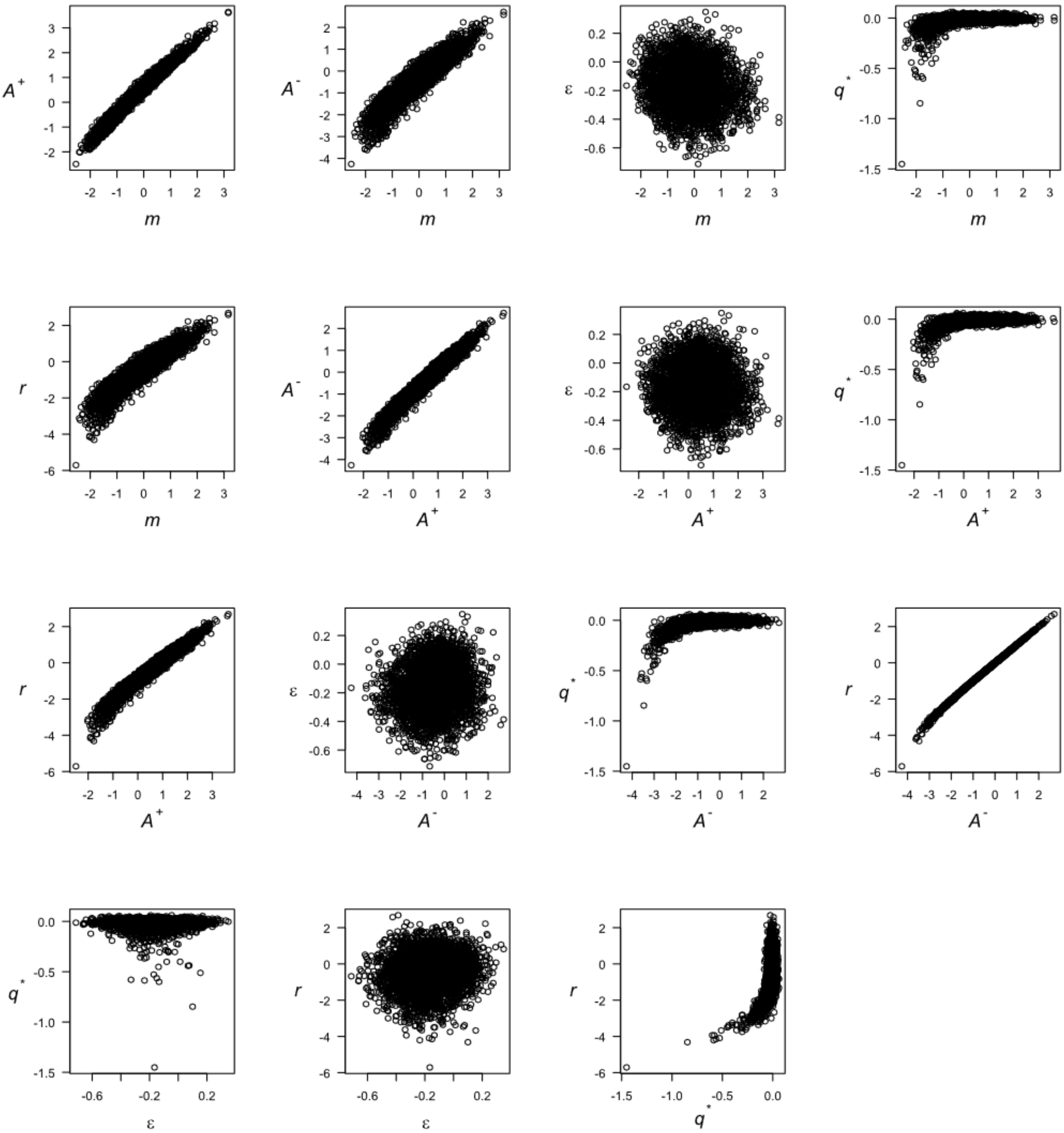

Fig C: Pairwise posterior distributions for spatial model using Normal approximation in case study one. *We plot the pairwise posterior distributions of all raw and derived parameter pairwise combinations.*

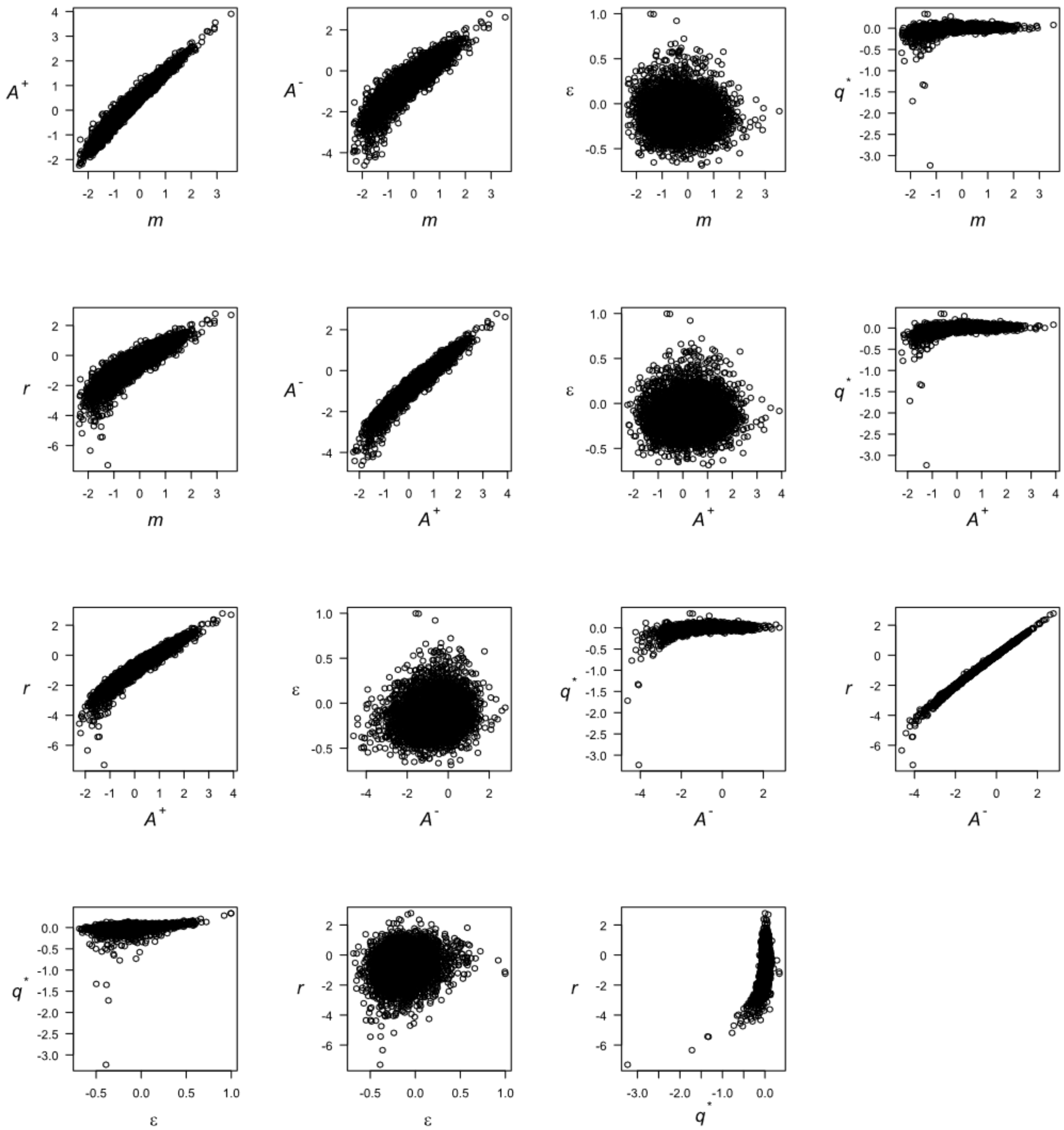

Fig D: Pairwise posterior distributions for spatial model using Poisson approximation in case study one. *We plot the pairwise posterior distributions of all raw and derived parameter pairwise combinations.*

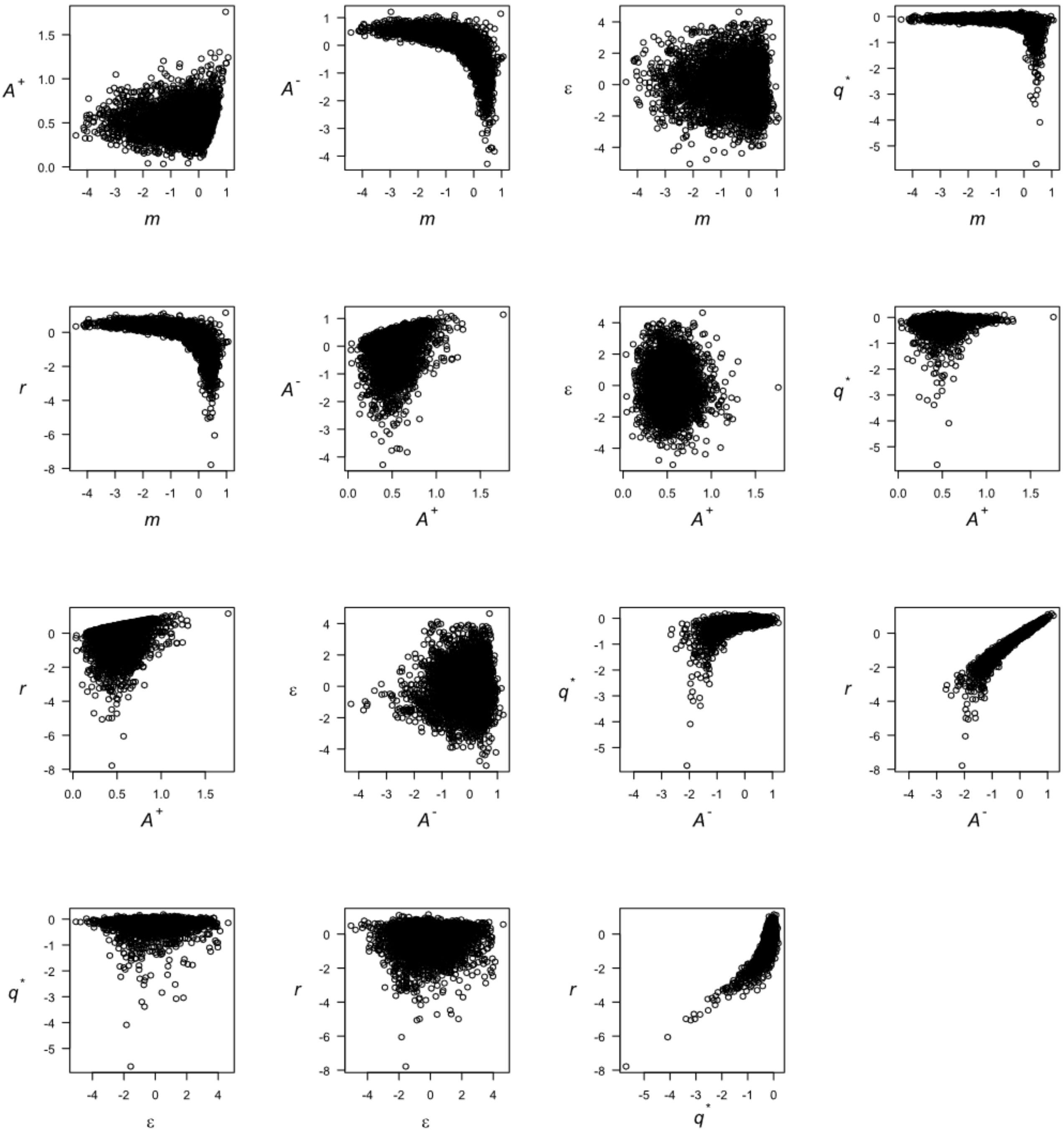

Fig E: Pairwise posterior distributions for non-spatial model in the unmarked data example in case study two. *We plot the pairwise posterior distributions of all raw and derived parameter pairwise combinations.*

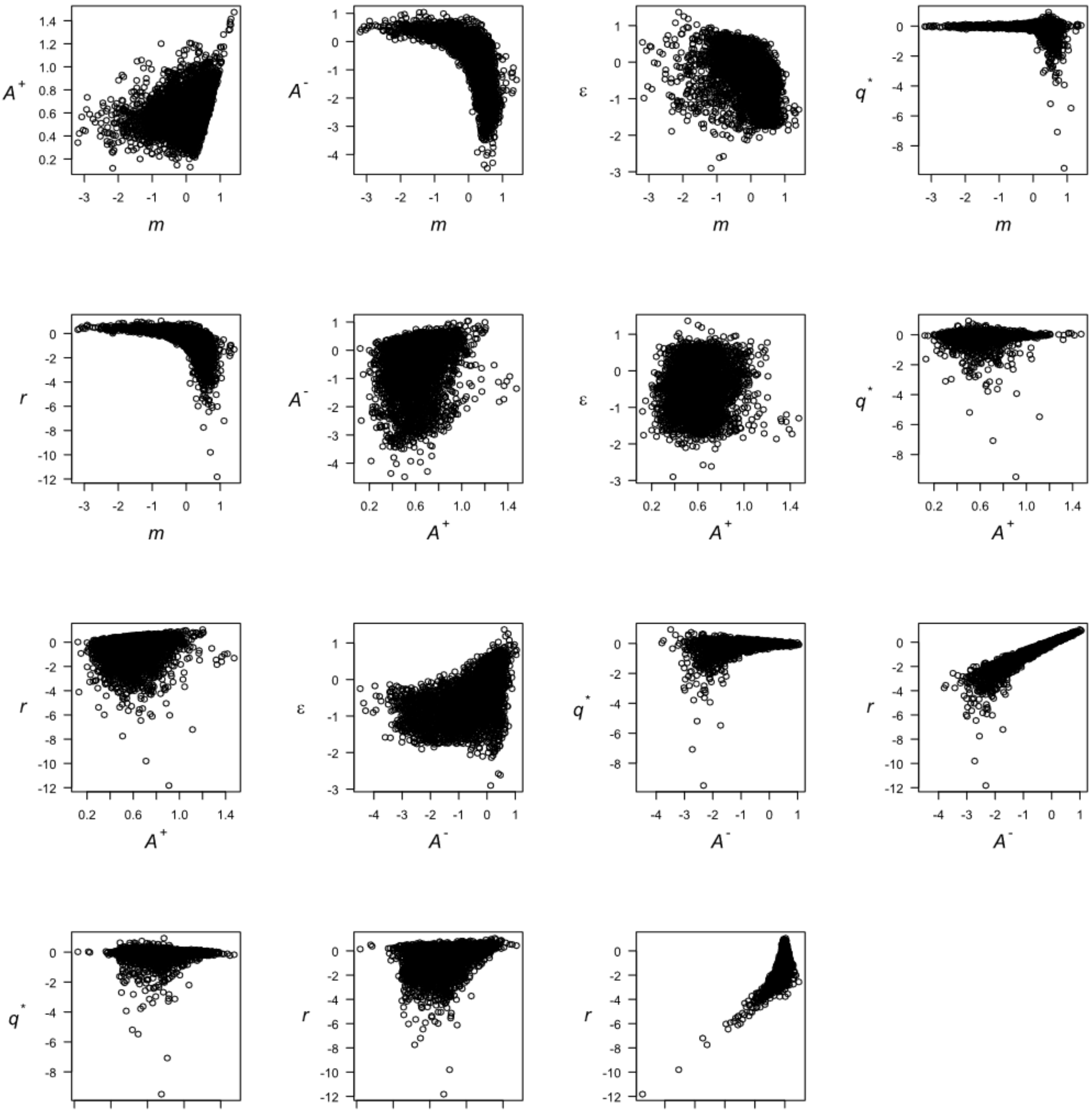

Fig F: Pairwise posterior distributions for spatial model using Normal approximation in the unmarked data example in case study two. *We plot the pairwise posterior distributions of all raw and derived parameter pairwise combinations.*

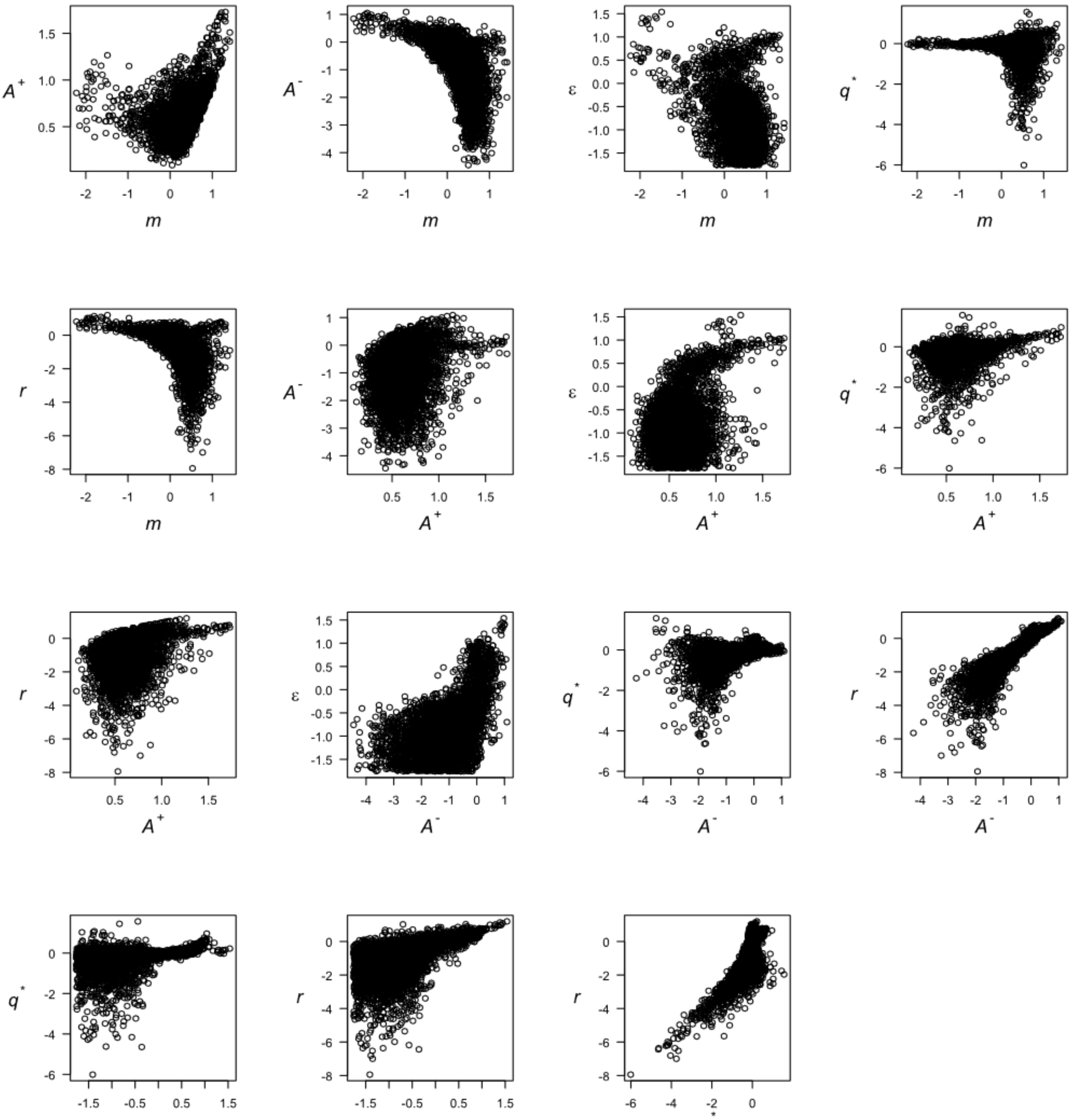

Fig G: Pairwise posterior distributions for spatial model using Poisson approximation in the unmarked data example in case study two. *We plot the pairwise posterior distributions of all raw and derived parameter pairwise combinations.*

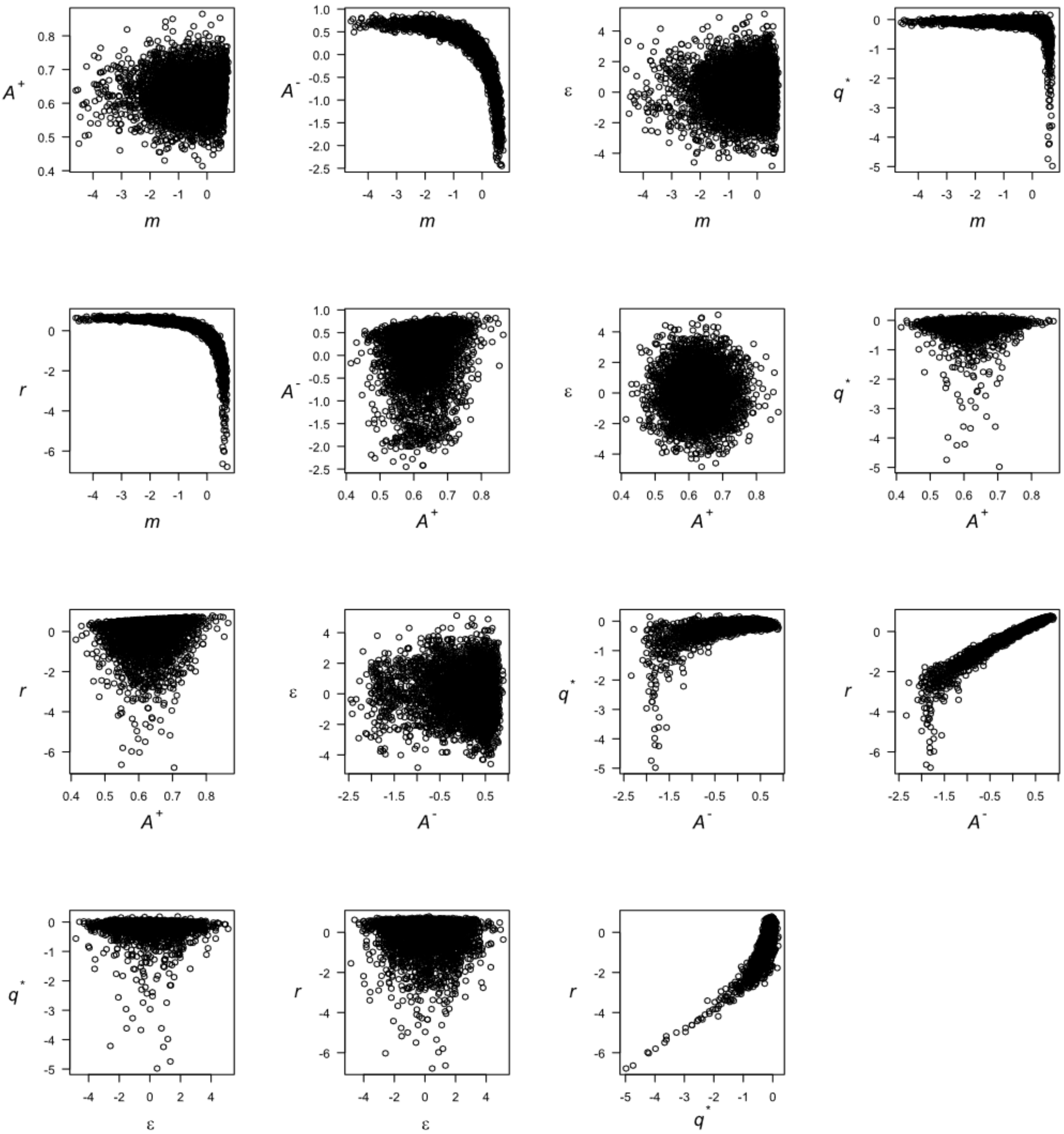

Fig H: Pairwise posterior distributions for non-spatial model in the marked data example in case study two. *We plot the pairwise posterior distributions of all raw and derived parameter pairwise combinations.*

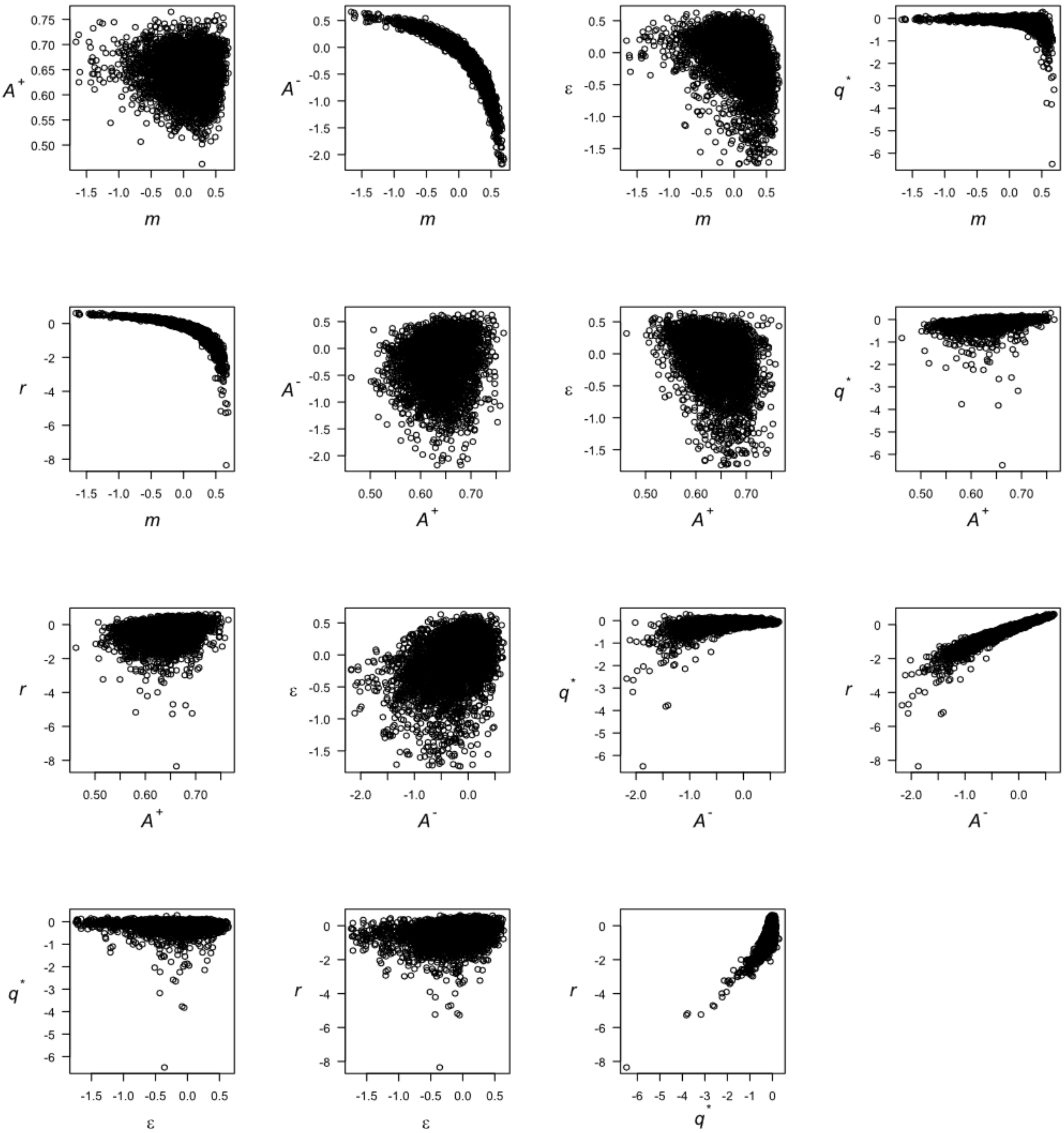

Fig I: Pairwise posterior distributions for spatial model using Normal approximation in the marked data example in case study two. *We plot the pairwise posterior distributions of all raw and derived parameter pairwise combinations.*

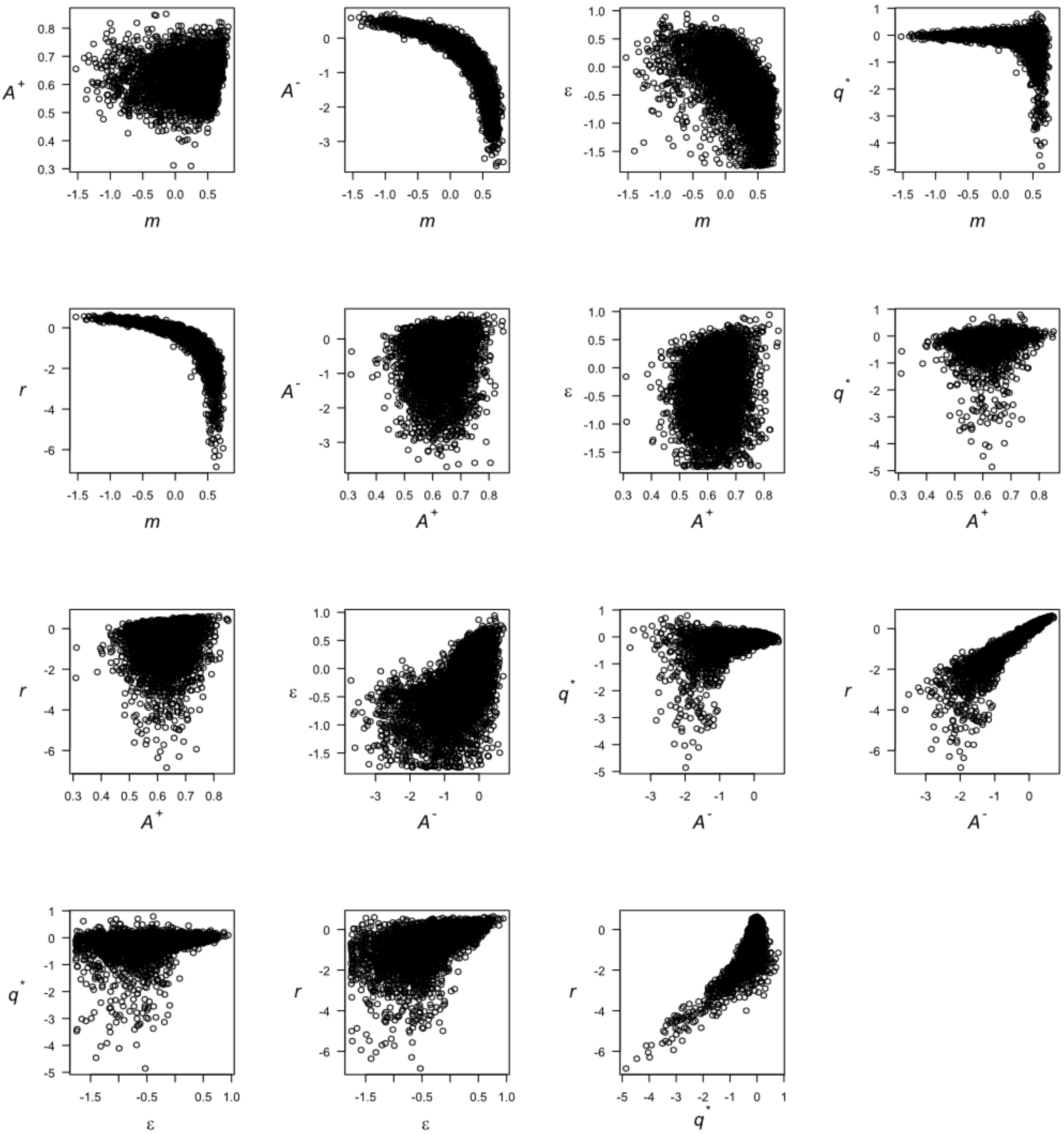

Fig J: Pairwise posterior distributions for spatial model using Poisson approximation in the marked data example in case study two. *We plot the pairwise posterior distributions of all raw and derived parameter pairwise combinations.*

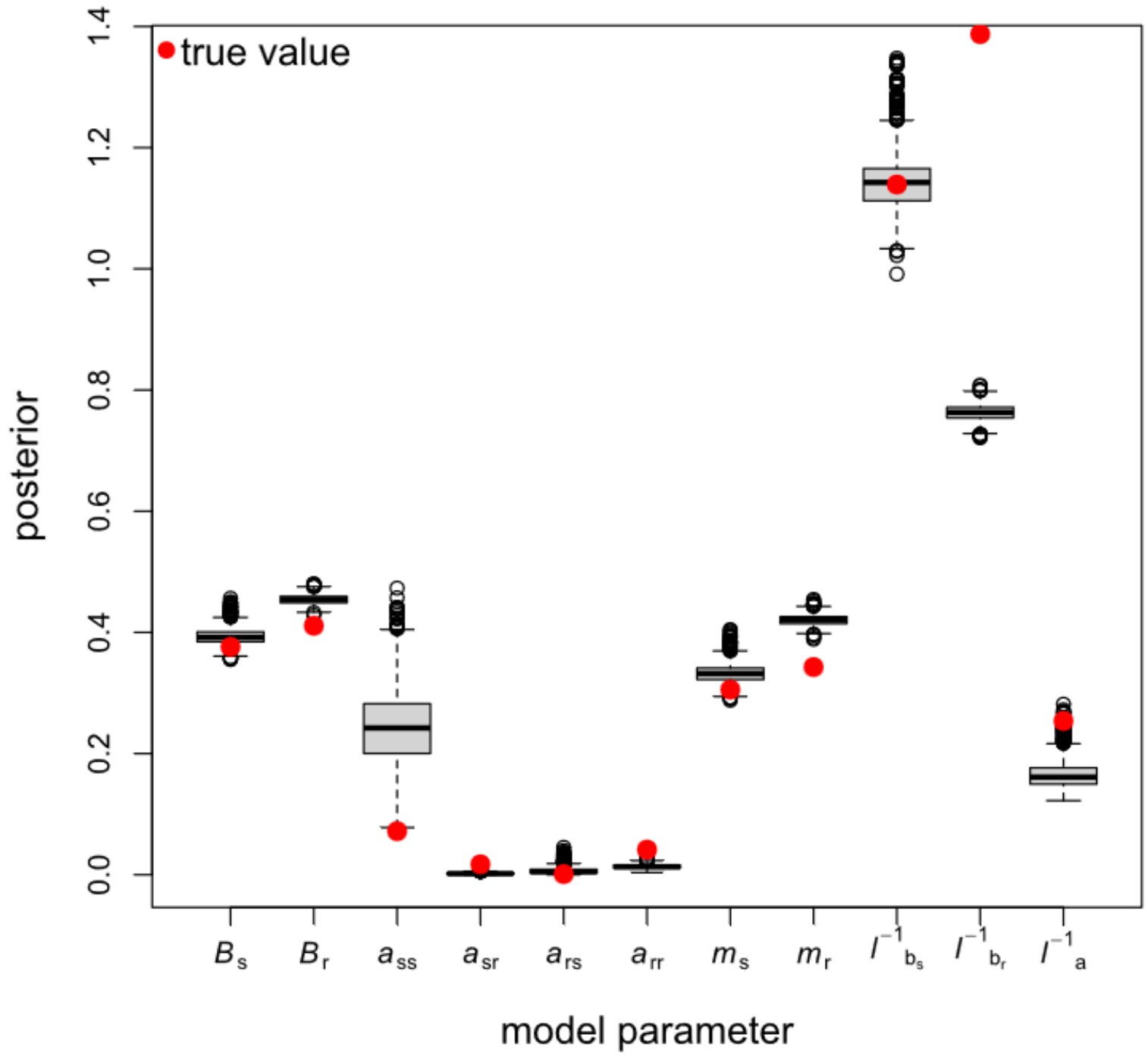

Fig K: Results of the validation of the cancer case study. *We take one posterior predictive simulation (drug concentration 0.0 nM and both cell types initially present) of the cancer case study and infer the parameter values used in the simulation. True parameter values are illustrated with red dots and the posterior distributions with boxplots.*

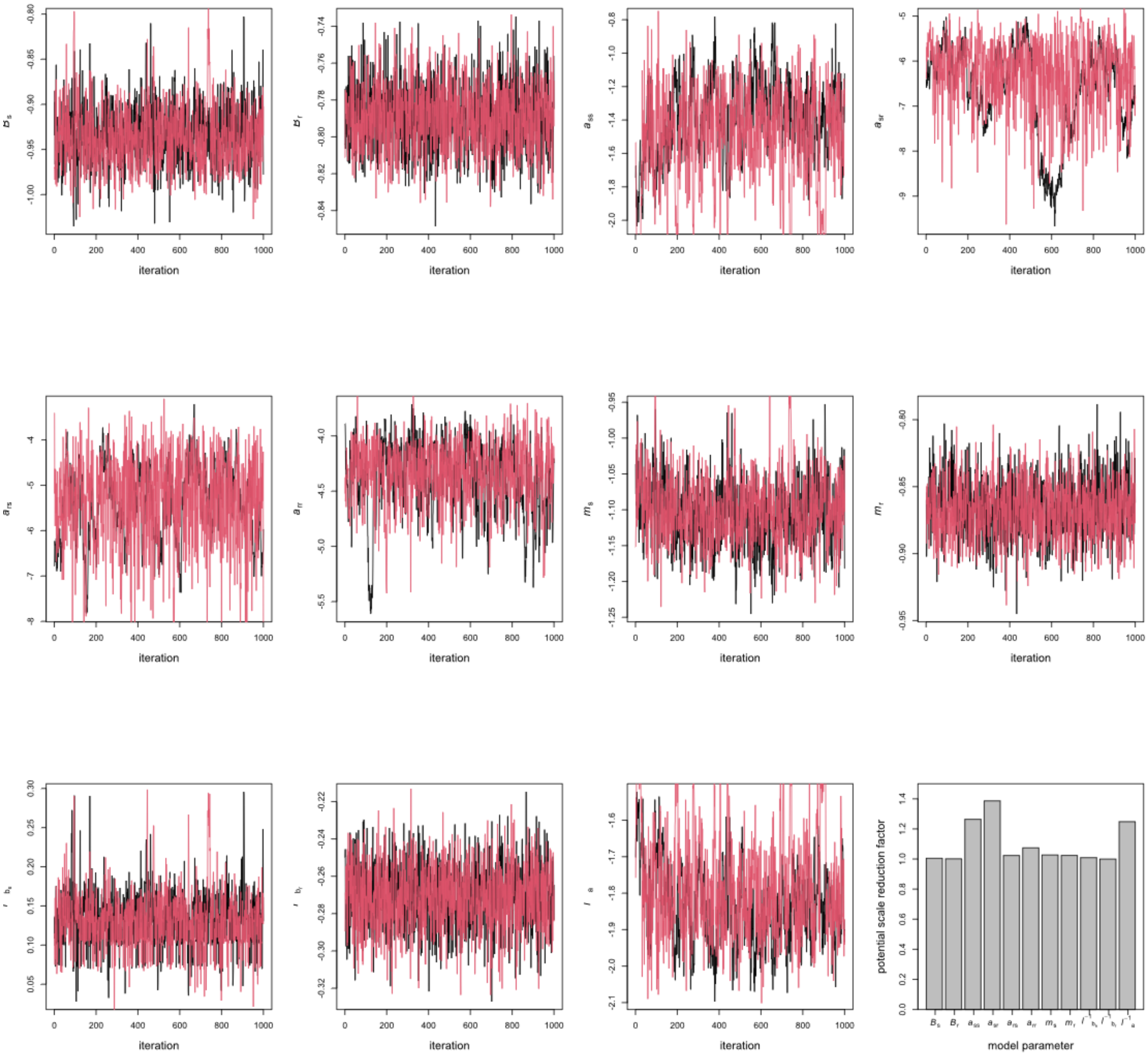

Fig L: MCMC convergence and mixing for the validation of the cancer case study. *2 chains were run for 11,000 iterations, of which we discarded the first 1000 iterations during which adaptation was applied, and thinned the remaining by 10 to obtain 1000 samples per chain. If visual inspection of the trace plots suggested that the chains were still in a transient phase, we continued the MCMC algorithm for additional 1000 adaptive iterations and additional 10,000 iterations which thinned by 10 obtained 1000 samples. The trace of the two chains and the mean of the potential scale reduction factor are reported.*

6 Justification of formula (4)

6.1 Notations and main statement

We consider the Euclidean space $\mathbb{R}^n$, $n \geq 1$. We interpret elements from $\mathbb{R}^n$ as column-vectors: $X = (x_1, \dots, x_n)^T \in \mathbb{R}^n$. Let $\mathcal{M}_{n,m}$ denote the space of $n \times m$ matrices.

For any two vector spaces $\mathtt{X}$ and $\mathtt{Y}$, we denote by $\mathcal{L}(\mathtt{X}, \mathtt{Y})$ the vector space of linear mappings from $\mathtt{X}$ to $\mathtt{Y}$. In particular, $\mathcal{M}_{n,m} = \mathcal{L}(\mathbb{R}^m, \mathbb{R}^n)$.

Consider the Hilbert space L^2 of random vectors $X \in \mathbb{R}^n$ with finite second moments: $\mathbb{E}[\|X\|^2] < \infty$ and the inner product given by $\mathbb{E}[X_1^T X_2]$ for $X_1, X_2 \in L^2$ (note that here X_1^T is a row-vector).

Let Y be a random vector in $\mathbb{R}^k$ with $1 \leq k \leq n$. Denote by L^2_Y the closed subspace of L^2 which consists of $\sigma(Y)$-measurable random vectors from L^2, i.e. $X \in L^2_Y$ iff $X \in L^2$ and there exists a Borel mapping $f : \mathbb{R}^k \to \mathbb{R}^n$ with $X = f(Y)$.

Let $Z \in L^2$. It is well-known that $\mathbb{E}[Z \mid Y]$ is the orthogonal projection of Z on L^2_Y, i.e.

$$\mathbb{E}\big[W^T \big(Z - \mathbb{E}[Z \mid Y]\big)\big] = 0 \tag{1}$$

for all $W \in L^2_Y$; moreover,

$$\mathbb{E}\big[\mathbb{E}[Z \mid Y]\big] = \mathbb{E}[Z]. \tag{2}$$

We denote also

$$\widetilde{Y} := Y - \mathbb{E}[Y], \qquad \Sigma_{ZY} := \operatorname{Cov}(Z, Y) \in \mathcal{M}_{n,k},$$
$$\widetilde{Z} := Z - \mathbb{E}[Z], \qquad \Sigma_{YY} := \operatorname{Cov}(Y, Y) \in \mathcal{M}_{k,k}.$$

In what follows, we assume additionally that

$$\mathbb{E}\big[\|Y\|^4\big] < \infty, \tag{3}$$

so that all mixed moments of $\widetilde{Y}$ up to the fourth order are finite.

Let $\mathcal{S}_k \subset \mathcal{M}_{k,k}$ denote the subspace of symmetric matrices; its dimension is

$$\varkappa := \frac{k(k+1)}{2}. \tag{4}$$

Let us introduce the mapping $T \in \mathcal{L}(\mathbb{R}^k, \mathcal{S}_k)$ by

$$(Tv)_{ij} = \sum_{m=1}^{k} \mathbb{E}[\widetilde{Y}_i \widetilde{Y}_j \widetilde{Y}_m] v_m, \qquad 1 \leq i, j \leq k. \tag{5}$$

(Note that indeed $Tv \in \mathcal{S}_k$, since $\mathbb{E}[\widetilde{Y}_i \widetilde{Y}_j \widetilde{Y}_m]$ is symmetric in i, j.) Then, we define

$$S(Y) := \widetilde{Y}\widetilde{Y}^T - \Sigma_{YY} - T(\Sigma_{YY}^{-1}\widetilde{Y}) \in \mathcal{S}_k, \tag{6}$$

or, entry-wise, for $1 \leq i, j \leq k$,

$$S(Y)_{ij} = \widetilde{Y}_i \widetilde{Y}_j - (\Sigma_{YY})_{ij} - \sum_{m=1}^{k} \mathbb{E}[\widetilde{Y}_i \widetilde{Y}_j \widetilde{Y}_m] (\Sigma_{YY}^{-1} \widetilde{Y})_m. \tag{7}$$

Next, let vech : $\mathcal{S}_k \to \mathbb{R}^{\varkappa}$ be the half-vectorization mapping which stacks the columns of the lower triangular part (including the main diagonal) of a symmetric matrix into a vector from $\mathbb{R}^{\varkappa}$; namely, for $M \in \mathcal{S}_k$, one has

$$\operatorname{vech}(M)_{\langle i,j\rangle} = M_{ij}, \qquad 1 \le j \le i \le k, \tag{8}$$

where

$$\langle i,j\rangle := i + (j-1)k - \frac{j(j-1)}{2}, \qquad 1 \le j \le i \le k. \tag{9}$$

The mapping $(i,j) \mapsto \langle i,j\rangle$ is a bijection between $\{(i,j) : 1 \le j \le i \le k\}$ and $\{1,\dots,\varkappa\}$; hence, vech is a linear bijection between $\mathcal{S}_k$ and $\mathbb{R}^{\varkappa}$.

We define then

$$s(Y) := \operatorname{vech}(S(Y)) \in \mathbb{R}^{\varkappa}. \tag{10}$$

Now, we can introduce

$$\Sigma_{ZS} := \mathbb{E}\big[\widetilde{Z}\,(s(Y))^T\big] = \operatorname{Cov}\big(Z, s(Y)\big) \in \mathcal{M}_{n,\varkappa}, \tag{11}$$

$$\Sigma_{SS} := \mathbb{E}\big[s(Y)(s(Y))^T\big] = \operatorname{Cov}\big(s(Y), s(Y)\big) \in \mathcal{M}_{\varkappa,\varkappa}. \tag{12}$$

Theorem 1. *Assume that* (3) *holds, and that* $\Sigma_{YY} \in \mathcal{M}_{k,k}$ *and* $\Sigma_{SS} \in \mathcal{M}_{\varkappa,\varkappa}$ *are invertible matrices. Then*

$$\mathbb{E}[Z \mid Y] = \mathbb{E}[Z] + C(Y - \mathbb{E}[Y]) + Ds(Y) + R_3(Y), \tag{13}$$

where

$$C := \Sigma_{ZY}\big(\Sigma_{YY}\big)^{-1} \in \mathcal{M}_{n,k}, \tag{14}$$

$$D := \Sigma_{ZS}\big(\Sigma_{SS}\big)^{-1} \in \mathcal{M}_{n,\varkappa}, \tag{15}$$

and $R_3(Y) \in L^2_Y$ *is orthogonal to all constant vectors and to all linear and quadratic polynomials of* $\widetilde{Y}$ *in* L^2_Y*. The entries of matrices* Σ_{ZS} *and* Σ_{SS} *can be found by formulas, for* $1 \le l \le n$, $1 \le j \le i \le k$, $1 \le q \le p \le k$*:*

$$(\Sigma_{ZS})_{l,\langle i,j\rangle} = \mathbb{E}[\widetilde{Z}_l \widetilde{Y}_i \widetilde{Y}_j] - \sum_{r=1}^{k} \mathbb{E}[\widetilde{Y}_i \widetilde{Y}_j \widetilde{Y}_r] C_{lr}, \tag{16}$$

and

$$(\Sigma_{SS})_{\langle i,j\rangle,\langle p,q\rangle} = \mathbb{E}[\widetilde{Y}_i \widetilde{Y}_j \widetilde{Y}_p \widetilde{Y}_q] - (\Sigma_{YY})_{ij}(\Sigma_{YY})_{pq} - \sum_{r=1}^{k}\sum_{u=1}^{k} \mathbb{E}[\widetilde{Y}_i \widetilde{Y}_j \widetilde{Y}_u]\,\mathbb{E}[\widetilde{Y}_p \widetilde{Y}_q \widetilde{Y}_r]\,(\Sigma_{YY}^{-1})_{ru}. \tag{17}$$

Remark 1. Note that (3) implies that, by the Cauchy–Schwarz inequality, $\mathbb{E}\big|\widetilde{Z}_l \widetilde{Y}_i \widetilde{Y}_j\big| < \infty$ for any $Z \in L^2$, $1 \le l \le n$, $1 \le i,j \le k$; hence, all terms in (16)–(17) are well-defined.

Remark 2. If one used instead the full vectorization $\mathrm{vec} : \mathcal{M}_{k,k} \to \mathbb{R}^{k^2}$, $\mathrm{vec}(M)_{i+k(j-1)} = M_{ij}$, $1 \le i, j \le k$, then, because of the symmetry of $S(Y)$, the coordinates of $\mathrm{vec}(S(Y))$ with the indices $i + k(j-1)$ and $j + k(i-1)$, $i \ne j$, would coincide almost surely. As a result, the covariance matrix of $\mathrm{vec}(S(Y))$ would be singular for any $k \ge 2$, and the invertibility assumption of Theorem 1 below would never hold. The half-vectorization removes these linearly dependent coordinates. Nevertheless, the invertibility of Σ_{SS} remains an additional non-degeneracy assumption on the distribution of Y.

6.2 Proof of Theorem 1

We construct the first terms of an orthogonal expansion of the centred (i.e. with zero mean) vector $\mathbb{E}[Z \mid Y] - \mathbb{E}[Z]$ through "powers" of $\widetilde{Y}$:

$$\mathbb{E}[Z \mid Y] - \mathbb{E}[Z] = r_1(Y) + r_2(Y) + R_3(Y), \tag{18}$$

where, for $m = 1, 2$, $r_m(Y) \in L^2_Y$ is a "polynomial" of order m of $\widetilde{Y}$ such that

$$\mathbb{E}[r_1(Y)] = \mathbb{E}[r_2(Y)] = 0, \qquad \mathbb{E}[r_2(Y)^T r_1(Y)] = 0, \tag{19}$$

and $R_3(Y)$ is orthogonal in L^2 to all constant vectors and to all such polynomials of orders 1 and 2.

It corresponds to the beginning of the orthogonal decomposition of L^2_Y:

$$L^2_Y = H^0_Y \oplus H^1_Y \oplus H^2_Y \oplus \left(H^0_Y \oplus H^1_Y \oplus H^2_Y\right)^\perp, \tag{20}$$

where $H^0_Y := \mathbb{R}^n$ (the space of "constant random vectors") and, for $m \ge 1$, H^m_Y is a subspace of the space of all polynomial expressions of order m of $\widetilde{Y}$ which are orthogonal to all polynomials of lower orders.

Remark 3. If polynomials of $\widetilde{Y}$ were dense in L^2_Y — which requires additional assumptions on the distribution of Y (e.g. a bounded support or finite exponential moments) and may fail otherwise (e.g. for a lognormal Y) — one would obtain the full orthogonal decomposition $L^2_Y = \bigoplus_{m \ge 0} H^m_Y$ and, correspondingly, the full expansion $\mathbb{E}[Z \mid Y] - \mathbb{E}[Z] = \sum_{m \ge 1} r_m(Y)$ in L^2. We will not use this below.

Namely, let $H^1_Y := \mathrm{span}(Y - \mathbb{E}[Y])$, i.e. H^1_Y is the subspace of L^2_Y consisting of all (centred) random vectors of the form $A(Y - \mathbb{E}[Y])$ for some (deterministic) matrix $A \in \mathcal{M}_{n,k}$.

Lemma 2. *Let $X \in L^2$ be such that $X \perp A\widetilde{Y}$ for any $A \in \mathcal{M}_{n,k}$. Then*

$$\mathbb{E}[X \widetilde{Y}^T] = 0 \in \mathcal{M}_{n,k}. \tag{21}$$

Proof. We have that, for any $A \in \mathcal{M}_{n,k}$,

$$0 = \mathbb{E}[X^T (A\widetilde{Y})] = \mathbb{E}\big[\mathrm{tr}((A\widetilde{Y})X^T)\big] = \mathrm{tr}\big(A\mathbb{E}[\widetilde{Y}X^T]\big).$$

It is well-known that if, for a matrix $M \in \mathcal{M}_{k,n}$, one has that $\mathrm{tr}(AM) = 0$ for any $A \in \mathcal{M}_{n,k}$, then $M = 0$. Therefore,

$$\mathbb{E}[\widetilde{Y} X^T] = 0,$$

and we can transpose both parts that implies (21). □

Let $r_1(Y)$ be the orthogonal projection of the centred random vector $\mathbb{E}[Z \mid Y] - \mathbb{E}[Z] \in L^2_Y$ onto H^1_Y, i.e.

$$\mathbb{E}\big[W^T\big(\mathbb{E}[Z \mid Y] - \mathbb{E}[Z] - r_1(Y)\big)\big] = 0, \tag{22}$$

for all $W \in H^1_Y$.

Proposition 3. *Suppose that Σ_{YY} is invertible. Then*

$$\boxed{r_1(Y) = C(Y - \mathbb{E}[Y]),} \tag{23}$$

with C given by (14).

Proof. Since $r_1(Y) \in H^1_Y$, we have that (23) holds for some matrix $C \in \mathcal{M}_{n,k}$. Then, each $W \in H^1_Y$ satisfies $W = A\widetilde{Y}$ for some matrix $A \in \mathcal{M}_{n,k}$. Therefore, (22) reads

$$\mathbb{E}\big[(A\widetilde{Y})^T\big(\mathbb{E}[Z \mid Y] - \mathbb{E}[Z] - C\widetilde{Y}\big)\big] = 0 \tag{24}$$

for all $A \in \mathcal{M}_{n,k}$. By Lemma 2,

$$\mathbb{E}\big[\big(\mathbb{E}[Z \mid Y] - \mathbb{E}[Z] - C\widetilde{Y}\big)\widetilde{Y}^T\big] = 0, \tag{25}$$

$$\mathbb{E}\big[\big(\mathbb{E}[Z \mid Y] - \mathbb{E}[Z]\big)\widetilde{Y}^T\big] = C\mathbb{E}[\widetilde{Y}\widetilde{Y}^T]. \tag{26}$$

Next,

$$\mathbb{E}[\widetilde{Y}\widetilde{Y}^T] = \mathbb{E}\big[(Y - \mathbb{E}[Y])(Y - \mathbb{E}[Y])^T\big] = \mathrm{Cov}(Y, Y) = \Sigma_{YY}, \tag{27}$$

and

$$\begin{aligned}
\mathbb{E}\big[\big(\mathbb{E}[Z \mid Y] - \mathbb{E}[Z]\big)\widetilde{Y}^T\big] &= \mathbb{E}\big[\big(\mathbb{E}[Z \mid Y] - \mathbb{E}[Z]\big)(Y - \mathbb{E}[Y])^T\big] \\
&= \mathbb{E}\big[\mathbb{E}[Z \mid Y]Y^T\big] - \mathbb{E}\big[\mathbb{E}[Z \mid Y]\big]\mathbb{E}\big[Y^T\big] \\
&= \mathbb{E}\big[ZY^T\big] - \mathbb{E}\big[Z\big]\mathbb{E}\big[Y^T\big] \\
&= \mathrm{Cov}(Z, Y) = \Sigma_{ZY}.
\end{aligned} \tag{28}$$

Therefore, (26) reads

$$\Sigma_{ZY} = C\Sigma_{YY}. \tag{29}$$

Since Σ_{YY} is assumed to be invertible, (14) holds. ☐

Since $\mathbb{E}[A(Y - \mathbb{E}[Y])] = 0$, we have that $H^0_Y \perp H^1_Y$ in L^2_Y. We have, hence, the orthogonal decomposition

$$L^2_Y = H^0_Y \oplus H^1_Y \oplus H^{\geqslant 2}_Y, \tag{30}$$

where $H^{\geqslant 2}_Y := (H^0_Y \oplus H^1_Y)^\perp$ is the orthogonal complement to $H^0_Y \oplus H^1_Y$.

Corollary 4. *For any $Z \in L^2$, the following expansion of $\mathbb{E}[Z \mid Y] \in L^2_Y$ holds*

$$\boxed{\mathbb{E}[Z \mid Y] = \mathbb{E}[Z] + C(Y - \mathbb{E}[Y]) + R_2(Y),} \tag{31}$$

where C is given by (14) *and* $\boxed{R_2(Y) \perp (H^0_Y \oplus H^1_Y)}$.

We can continue the orthogonal expansion. Note that H^1_Y can be described as the subspace of elements from

$$P^1_Y := \operatorname{span}(H^0_Y, \widetilde{Y}) = \{c + A\widetilde{Y} : c \in \mathbb{R}^n, A \in \mathcal{M}_{n,k}\} \tag{32}$$

orthogonal to H^0_Y. Similarly, since $\widetilde{Y}\widetilde{Y}^T \in \mathcal{M}_{k,k}$, we consider the space

$$\begin{aligned} P^2_Y :&= \operatorname{span}(H^0_Y, \widetilde{Y}, \widetilde{Y}\widetilde{Y}^T) \\ &= \{c + A\widetilde{Y} + B(\widetilde{Y}\widetilde{Y}^T) : c \in \mathbb{R}^n, A \in \mathcal{M}_{n,k}, B \in \mathcal{L}(\mathcal{S}_k, \mathbb{R}^n)\}. \end{aligned} \tag{33}$$

Next, we define H^2_Y as the set of vectors from P^2_Y orthogonal to $H^0_Y \oplus H^1_Y$.

Therefore, for an $X \in H^2_Y$, we have that $X = c + A\widetilde{Y} + B(\widetilde{Y}\widetilde{Y}^T)$ for some $c \in \mathbb{R}^n$, $A \in \mathcal{M}_{n,k}$, $B \in \mathcal{L}(\mathcal{S}_k, \mathbb{R}^n)$ and also $X \perp H^0_Y$ and $X \perp H^1_Y$. The first orthogonality means that

$$\begin{aligned} 0 &= \mathbb{E}[X] = c + B\Sigma_{YY}, \\ c &= -B\Sigma_{YY}. \end{aligned}$$

Next, $X \perp H^1_Y$ means that $X \perp A\widetilde{Y}$ for each $A \in \mathcal{M}_{n,k}$. By Lemma 2,

$$\begin{aligned} 0 = \mathbb{E}[X\widetilde{Y}^T] &= \mathbb{E}[(c + A\widetilde{Y} + B(\widetilde{Y}\widetilde{Y}^T))\widetilde{Y}^T] \\ &= A\Sigma_{YY} + \mathbb{E}[B(\widetilde{Y}\widetilde{Y}^T)\widetilde{Y}^T] \end{aligned} \tag{34}$$

Consider the action of B: it is a tensor $(B_{lij})_{1\le l\le n,\, 1\le j\le i\le k}$ such that, for any $M \in \mathcal{S}_k$, we have

$$\big(B(M)\big)_l = \sum_{1\le j\le i\le k} B_{lij} M_{ij}, \qquad 1 \le l \le n. \tag{35}$$

Therefore, since $(\widetilde{Y}\widetilde{Y}^T)_{ij} = \widetilde{Y}_i\widetilde{Y}_j$, we have that:

$$\big(B(\widetilde{Y}\widetilde{Y}^T)\widetilde{Y}^T\big)_{lm} = \sum_{1\le j\le i\le k} B_{lij}\widetilde{Y}_i\widetilde{Y}_j\widetilde{Y}_m, \qquad 1 \le l \le n, 1 \le m \le k;$$

hence, from (34), we obtain:

$$A\Sigma_{YY} = -B\kappa_{YYY}, \tag{36}$$

where

$$(B\kappa_{YYY})_{l,m} = \sum_{1\le j\le i\le k} B_{lij}\mathbb{E}[\widetilde{Y}_i\widetilde{Y}_j\widetilde{Y}_m], \qquad 1 \le l \le n, 1 \le m \le k. \tag{37}$$

Therefore,

$$A = -(B\kappa_{YYY})\Sigma^{-1}_{YY}, \tag{38}$$

and hence, $X \in H^2_Y$ iff

$$X = -B\Sigma_{YY} - (B\kappa_{YYY})\Sigma^{-1}_{YY}\widetilde{Y} + B\widetilde{Y}\widetilde{Y}^T. \tag{39}$$

Recall that $T \in \mathcal{L}(\mathbb{R}^k, \mathcal{S}_k)$ is given by (5). Then, since $B\kappa_{YYY} \in \mathcal{M}_{n,k}$, we have for $v \in \mathbb{R}^k$,

$$\begin{aligned} \big((B\kappa_{YYY})v\big)_l &= \sum_{m=1}^{k}(B\kappa_{YYY})_{l,m}v_m = \sum_{m=1}^{k}\sum_{1\le j\le i\le k} B_{lij}\mathbb{E}[\widetilde{Y}_i\widetilde{Y}_j\widetilde{Y}_m]v_m \\ &= \sum_{1\le j\le i\le k} B_{lij}(Tv)_{ij} = \big(B(Tv)\big)_l, \end{aligned}$$

for all $1 \le l \le n$, i.e.

$$(B\kappa_{YYY})v = B(Tv), \qquad v \in \mathbb{R}^k. \tag{40}$$

As a result, $X \in H_Y^2$ iff

$$X = B\big(S(Y)\big), \tag{41}$$

where $S(Y)$ is given by (6).

By Corollary 4,

$$R_2(Y) = \mathbb{E}[Z \mid Y] - \mathbb{E}[Z] - C(Y - \mathbb{E}[Y]) \perp H_Y^0 \oplus H_Y^1. \tag{42}$$

Let $r_2(Y)$ be the next term of the orthogonal expansion of $\mathbb{E}[Z \mid Y]$, i.e. let $r_2(Y)$ be the projection of $R_2(Y)$ on H_Y^2.

Note that, for $M \in \mathcal{S}_k$ and $1 \le l \le n$,

$$\big(B(M)\big)_l = \sum_{1\le j\le i\le k} B_{lij} M_{ij} = \sum_{1\le j\le i\le k} B_{lij} \operatorname{vech}(M)_{\langle i,j\rangle}.$$

Therefore, there exists a unique matrix $D = D(B) \in \mathcal{M}_{n,\varkappa}$ such that

$$B(M) = D \operatorname{vech}(M), \qquad M \in \mathcal{S}_k;$$

namely, $D_{l,\langle i,j\rangle} := B_{lij}$, $1 \le l \le n$, $1 \le j \le i \le k$. Conversely, every $D \in \mathcal{M}_{n,\varkappa}$ corresponds in this way to some $B \in \mathcal{L}(\mathcal{S}_k, \mathbb{R}^n)$. Since $S(Y) \in \mathcal{S}_k$, we obtain, as a result,

$$B(S(Y)) = Ds(Y). \tag{43}$$

Now, we can find the conditions on D to have that

$$r_2(Y) = D\, s(Y) \tag{44}$$

would be the orthogonal projection of $R_2(Y)$, given by (42), on H_Y^2; it can be done similarly to how it was obtained for $r_1(Y)$.

Namely, since $r_2(Y) \in H_Y^2$, (44) holds with some $D \in \mathcal{M}_{n,\varkappa}$. Next, $D\, s(Y)$ is the orthogonal projection of $R_2(Y)$ on H_Y^2 iff

$$R_2(Y) - D\, s(Y) \perp W, \qquad W \in H_Y^2.$$

Each $W \in H_Y^2$ can be represented as $W = As(Y)$, for some $A \in \mathcal{M}_{n,\varkappa}$. Therefore,

$$R_2(Y) - D\, s(Y) \perp As(Y)$$

for each $A \in \mathcal{M}_{n,\varkappa}$. Applying Lemma 2 with $\mathbb{R}^k$ replaced by $\mathbb{R}^\varkappa$ and $\widetilde{Y}$ replaced by $s(Y)$, we have that

$$\mathbb{E}\big[\big(R_2(Y) - D\, s(Y)\big)s(Y)^T\big] = 0 \in \mathcal{M}_{n,\varkappa}. \tag{45}$$

Next, by (6),

$$\mathbb{E}[S(Y)] = \mathbb{E}[\widetilde{Y}\widetilde{Y}^T] - \Sigma_{YY} - T(\Sigma_{YY}^{-1}\mathbb{E}[\widetilde{Y}]) = 0 \in \mathcal{S}_k,$$

therefore, by (10),

$$\mathbb{E}[s(Y)] = 0 \in \mathbb{R}^\varkappa. \tag{46}$$

Furthermore, $s(Y)\widetilde{Y}^T \in \mathcal{M}_{\varkappa,k}$, and, for $1 \le j \le i \le k$, $1 \le m \le k$,

$$\mathbb{E}\big[s(Y)\widetilde{Y}^T\big]_{\langle i,j\rangle,m} = \mathbb{E}\big[s(Y)_{\langle i,j\rangle}\widetilde{Y}_m\big] = \mathbb{E}\big[S(Y)_{ij}\widetilde{Y}_m\big], \tag{47}$$

where we used (8). By (7),

$$S(Y)_{ij} = \widetilde{Y}_i\widetilde{Y}_j - (\Sigma_{YY})_{ij} - \sum_{r=1}^{k}\sum_{u=1}^{k}\mathbb{E}[\widetilde{Y}_i\widetilde{Y}_j\widetilde{Y}_r](\Sigma_{YY}^{-1})_{ru}\widetilde{Y}_u. \tag{48}$$

Hence,

$$\mathbb{E}\big[S(Y)_{ij}\widetilde{Y}_m\big] = \mathbb{E}[\widetilde{Y}_i\widetilde{Y}_j\widetilde{Y}_m] - \sum_{r=1}^{k}\sum_{u=1}^{k}\mathbb{E}[\widetilde{Y}_i\widetilde{Y}_j\widetilde{Y}_r](\Sigma_{YY}^{-1})_{ru}\mathbb{E}[\widetilde{Y}_u\widetilde{Y}_m] = 0,$$

since

$$\begin{aligned}\sum_{r=1}^{k}\sum_{u=1}^{k}\mathbb{E}[\widetilde{Y}_i\widetilde{Y}_j\widetilde{Y}_r](\Sigma_{YY}^{-1})_{ru}\mathbb{E}[\widetilde{Y}_u\widetilde{Y}_m] &= \sum_{r=1}^{k}\sum_{u=1}^{k}\mathbb{E}[\widetilde{Y}_i\widetilde{Y}_j\widetilde{Y}_r](\Sigma_{YY}^{-1})_{ru}(\Sigma_{YY})_{um}\\ &= \sum_{r=1}^{k}\mathbb{E}[\widetilde{Y}_i\widetilde{Y}_j\widetilde{Y}_r](\Sigma_{YY}^{-1}\Sigma_{YY})_{rm}\\ &= \sum_{r=1}^{k}\mathbb{E}[\widetilde{Y}_i\widetilde{Y}_j\widetilde{Y}_r]\delta_{rm} = \mathbb{E}[\widetilde{Y}_i\widetilde{Y}_j\widetilde{Y}_m].\end{aligned}$$

Therefore, by (47),

$$\mathbb{E}[s(Y)\widetilde{Y}^T] = 0 \in \mathcal{M}_{\varkappa,k},$$

and taking transposition, we obtain that

$$\mathbb{E}[\widetilde{Y}s(Y)^T] = 0 \in \mathcal{M}_{k,\varkappa},$$

and therefore,

$$\mathbb{E}[(C\widetilde{Y})s(Y)^T] = 0 \in \mathcal{M}_{n,\varkappa}, \tag{49}$$

where $C \in \mathcal{M}_{n,k}$ is given by (14).

Then, from (45), (46), (49), and (42), we have a simplified condition on D:

$$\mathbb{E}\Big[\big(\mathbb{E}[Z \mid Y] - Ds(Y)\big)s(Y)^T\Big] = 0 \in \mathcal{M}_{n,\varkappa},$$

that gives the normal equation:

$$\mathbb{E}\big[\mathbb{E}[Z \mid Y]s(Y)^T\big] = D\mathbb{E}[s(Y)s(Y)^T];$$

where we used that $(Ds(Y))s(Y)^T = D(s(Y)s(Y)^T) \in \mathcal{M}_{n,\varkappa}$. Since $s(Y)$ is $\sigma(Y)$-measurable,

$$\mathbb{E}\big[\mathbb{E}[Z \mid Y]s(Y)^T\big] = \mathbb{E}\big[\mathbb{E}[Zs(Y)^T \mid Y]\big] = \mathbb{E}[Zs(Y)^T],$$

and hence we can rewrite the normal equation:

$$\mathbb{E}\big[Zs(Y)^T\big] = D\mathbb{E}[s(Y)s(Y)^T].$$

By (46), we can replace here Z by $\widetilde{Z} = Z - \mathbb{E}[Z]$, and therefore, we get by (11) that

$$\Sigma_{ZS} = D\Sigma_{SS}.$$

Since we assume that Σ_{SS} is invertible, we then get (15).

As a result,

$$r_2(Y) = \Sigma_{ZS}(\Sigma_{SS})^{-1}\operatorname{vech}(S(Y)) = \Sigma_{ZS}(\Sigma_{SS})^{-1}s(Y). \tag{50}$$

We can compute also the entries of $\Sigma_{ZS} \in \mathcal{M}_{n,\varkappa}$. Namely, for $1 \le l \le n$ and $1 \le j \le i \le k$, we have by (11) and (48) that

$$\begin{aligned}(\Sigma_{ZS})_{l,\langle i,j\rangle} &= \mathbb{E}[\widetilde{Z}_l(S(Y))_{ij}] \\ &= \mathbb{E}[\widetilde{Z}_l\widetilde{Y}_i\widetilde{Y}_j] - \sum_{r=1}^{k}\sum_{u=1}^{k}\mathbb{E}[\widetilde{Y}_i\widetilde{Y}_j\widetilde{Y}_r](\Sigma_{YY}^{-1})_{ru}\mathbb{E}[\widetilde{Z}_l\widetilde{Y}_u].\end{aligned}$$

Since Σ_{YY} is a symmetric matrix, $(\Sigma_{YY}^{-1})_{ru} = (\Sigma_{YY}^{-1})_{ur}$, and then

$$\sum_{u=1}^{k}(\Sigma_{YY}^{-1})_{ur}\mathbb{E}[\widetilde{Z}_l\widetilde{Y}_u] = (\Sigma_{ZY}\Sigma_{YY}^{-1})_{lr} = C_{lr},$$

where C is given by (14). Therefore, (16) holds.

Next, we compute the entries of $\Sigma_{SS} \in \mathcal{M}_{\varkappa,\varkappa}$. We have, for $1 \le j \le i \le k$ and $1 \le q \le p \le k$,

$$(\Sigma_{SS})_{\langle i,j\rangle,\langle p,q\rangle} = \mathbb{E}[s(Y)_{\langle i,j\rangle}s(Y)_{\langle p,q\rangle}] = \mathbb{E}[S(Y)_{ij}S(Y)_{pq}].$$

Denote

$$\begin{aligned}\widetilde{\Sigma}_{ij}(Y) &:= \widetilde{Y}_i\widetilde{Y}_j - (\Sigma_{YY})_{ij}, \\ H_{ij}(Y) &:= \sum_{r=1}^{k}\sum_{u=1}^{k}\mathbb{E}[\widetilde{Y}_i\widetilde{Y}_j\widetilde{Y}_r](\Sigma_{YY}^{-1})_{ru}\widetilde{Y}_u,\end{aligned}$$

so that, by (48),

$$S(Y)_{ij} = \widetilde{\Sigma}_{ij}(Y) - H_{ij}(Y).$$

Then

$$\begin{aligned}\mathbb{E}[S(Y)_{ij}S(Y)_{pq}] &= \mathbb{E}[(\widetilde{\Sigma}_{ij}(Y) - H_{ij}(Y))(\widetilde{\Sigma}_{pq}(Y) - H_{pq}(Y))] \\ &= \mathbb{E}[\widetilde{\Sigma}_{ij}(Y)\widetilde{\Sigma}_{pq}(Y)] - \mathbb{E}[\widetilde{\Sigma}_{ij}(Y)H_{pq}(Y)] \\ &\quad - \mathbb{E}[\widetilde{\Sigma}_{pq}(Y)H_{ij}(Y)] + \mathbb{E}[H_{ij}(Y)H_{pq}(Y)]\end{aligned}$$

and since $\mathbb{E}[H_{ij}(Y)] = \mathbb{E}[H_{pq}(Y)] = 0$, we can continue

$$\begin{aligned}&= \mathbb{E}[\widetilde{\Sigma}_{ij}(Y)\widetilde{\Sigma}_{pq}(Y)] - \mathbb{E}[\widetilde{Y}_i\widetilde{Y}_jH_{pq}(Y)] \\ &\quad - \mathbb{E}[\widetilde{Y}_p\widetilde{Y}_qH_{ij}(Y)] + \mathbb{E}[H_{ij}(Y)H_{pq}(Y)].\end{aligned}$$

Next,

$$\mathbb{E}[\widetilde{\Sigma}_{ij}(Y)\widetilde{\Sigma}_{pq}(Y)] = \mathbb{E}[\widetilde{Y}_i\widetilde{Y}_j\widetilde{Y}_p\widetilde{Y}_q] - (\Sigma_{YY})_{ij}(\Sigma_{YY})_{pq},$$

and

$$\mathbb{E}[\widetilde{Y_i}\widetilde{Y_j}H_{pq}(Y)] = \sum_{r=1}^{k}\sum_{u=1}^{k}\mathbb{E}[\widetilde{Y_p}\widetilde{Y_q}\widetilde{Y_r}](\Sigma_{YY}^{-1})_{ru}\mathbb{E}[\widetilde{Y_i}\widetilde{Y_j}\widetilde{Y_u}].$$

Similarly,

$$\mathbb{E}[\widetilde{Y_p}\widetilde{Y_q}H_{ij}(Y)] = \sum_{r=1}^{k}\sum_{u=1}^{k}\mathbb{E}[\widetilde{Y_i}\widetilde{Y_j}\widetilde{Y_r}](\Sigma_{YY}^{-1})_{ru}\mathbb{E}[\widetilde{Y_p}\widetilde{Y_q}\widetilde{Y_u}], \tag{51}$$

and

$$\mathbb{E}[H_{ij}(Y)H_{pq}(Y)] = \sum_{r=1}^{k}\sum_{u=1}^{k}\sum_{r'=1}^{k}\sum_{u'=1}^{k}\mathbb{E}[\widetilde{Y_i}\widetilde{Y_j}\widetilde{Y_r}](\Sigma_{YY}^{-1})_{ru}\mathbb{E}[\widetilde{Y_p}\widetilde{Y_q}\widetilde{Y_{r'}}](\Sigma_{YY}^{-1})_{r'u'}\mathbb{E}[\widetilde{Y_u}\widetilde{Y_{u'}}].$$

Since $\mathbb{E}[\widetilde{Y_u}\widetilde{Y_{u'}}] = (\Sigma_{YY})_{uu'}$, and

$$\sum_{u=1}^{k}\sum_{u'=1}^{k}(\Sigma_{YY}^{-1})_{ru}(\Sigma_{YY}^{-1})_{r'u'}(\Sigma_{YY})_{uu'} = \sum_{u=1}^{k}(\Sigma_{YY}^{-1})_{ru}\delta_{ur'} = (\Sigma_{YY}^{-1})_{rr'},$$

we have

$$\mathbb{E}[H_{ij}(Y)H_{pq}(Y)] = \sum_{r=1}^{k}\sum_{r'=1}^{k}\mathbb{E}[\widetilde{Y_i}\widetilde{Y_j}\widetilde{Y_r}]\mathbb{E}[\widetilde{Y_p}\widetilde{Y_q}\widetilde{Y_{r'}}](\Sigma_{YY}^{-1})_{rr'} = \mathbb{E}[\widetilde{Y_p}\widetilde{Y_q}H_{ij}(Y)],$$

by (51).

As a result, we obtain (17).

This finishes the proof of Theorem 1.

6.3 Beyond mean field expansion: linear term

For $Z = (Z_1, \ldots, Z_n)^T \in \mathbb{R}^n$, we have

$$\mathbb{E}[Z_i] = q(Z)_i + \varepsilon^d p(Z)_i + o(\varepsilon^d), \tag{52}$$

$$\mathrm{Var}(Z_i) = \frac{1}{\delta}q(Z)_i + \varepsilon^d\frac{1}{\delta}p(Z)_i + o(\varepsilon^d), \tag{53}$$

$$\mathrm{Cov}(Z_i, Z_j) = \varepsilon^d g(Z,Z)_{ij} + o(\varepsilon^d), \qquad i \neq j. \tag{54}$$

We write, for brevity, $q_i := q(Z)_i$, $p_i := p(Z)_i$, $g_{ij} := g(Z,Z)_{ij}$, and consider the vectors $q := (q_1, \ldots, q_n)^T$, $p := (p_1, \ldots, p_n)^T \in \mathbb{R}^n$, together with the matrices $Q, P, G \in \mathcal{M}_{n,n}$, given by

$$Q = \mathrm{diag}(q_1, \ldots, q_n), \tag{55}$$

$$P = \mathrm{diag}(p_1, \ldots, p_n), \tag{56}$$

$$G_{ij} = g_{ij}, \quad i \neq j, \qquad G_{ii} = 0. \tag{57}$$

Then,

$$\mathbb{E}[Z] = q + \varepsilon^d p + o(\varepsilon^d), \tag{58}$$

$$\mathrm{Cov}(Z,Z) = \delta^{-1}Q + \varepsilon^d(\delta^{-1}P + G) + o(\varepsilon^d). \tag{59}$$

For the analysis of the quadratic term below, we will additionally assume the following asymptotics of the third moments of $\widetilde{Z} = Z - \mathbb{E}[Z]$: for all a, for all $a \neq b$, and for all pairwise distinct a, b, c in $\{1, \dots, n\}$, respectively,

$$\begin{aligned} \mathbb{E}[\widetilde{Z}_a^3] &= \frac{1}{\delta^2}\big(q_a + \varepsilon^d p_a\big) + o(\varepsilon^d), \\ \mathbb{E}[\widetilde{Z}_a^2 \widetilde{Z}_b] &= \frac{\varepsilon^d}{\delta}\, g_{ab} + o(\varepsilon^d), \\ \mathbb{E}[\widetilde{Z}_a \widetilde{Z}_b \widetilde{Z}_c] &= o(\varepsilon^d). \end{aligned} \tag{60}$$

Suppose that

$$Y = UZ, \qquad U \in \mathcal{M}_{k,n}, \qquad \operatorname{rank}(U) = k. \tag{61}$$

Then

$$\mathbb{E}[Y] = Uq + \varepsilon^d Up + o(\varepsilon^d), \tag{62}$$

and since

$$\begin{aligned} \operatorname{Cov}(Y, Y) &= \mathbb{E}[\widetilde{Y}\widetilde{Y}^T] \\ &= \mathbb{E}[U\widetilde{Z}(U\widetilde{Z})^T] = U\mathbb{E}[\widetilde{Z}\widetilde{Z}^T]U^T = U \operatorname{Cov}(Z, Z)U^T, \end{aligned}$$

and

$$\begin{aligned} \operatorname{Cov}(Z, Y) &= \mathbb{E}[\widetilde{Z}\widetilde{Y}^T] \\ &= \mathbb{E}[\widetilde{Z}(U\widetilde{Z})^T] = \mathbb{E}[\widetilde{Z}\widetilde{Z}^T]U^T = \operatorname{Cov}(Z, Z)U^T, \end{aligned}$$

we have

$$\Sigma_{YY} = \delta^{-1}UQU^T + \varepsilon^d(\delta^{-1}UPU^T + UGU^T) + o(\varepsilon^d), \tag{63}$$

$$\Sigma_{ZY} = \delta^{-1}QU^T + \varepsilon^d(\delta^{-1}PU^T + GU^T) + o(\varepsilon^d). \tag{64}$$

Assume that $q_i > 0$ for all $1 \le i \le n$. Then Q is positive definite and, since $\operatorname{rank}(U) = k$, the matrix UQU^T is positive definite, hence invertible; therefore, $\Sigma_{YY} = \delta^{-1}UQU^T + O(\varepsilon^d)$ is invertible for all sufficiently small $\varepsilon > 0$. To calculate its inverse, we note that, for any $B_0, B_1 \in \mathcal{M}_{k,k}$, with invertible B_0, one has

$$(B_0 + \varepsilon^d B_1)^{-1} = B_0^{-1} - \varepsilon^d B_0^{-1} B_1 B_0^{-1} + o(\varepsilon^d).$$

Therefore,

$$\Sigma_{YY}^{-1} = \delta(UQU^T)^{-1} - \varepsilon^d(UQU^T)^{-1}(\delta UPU^T + \delta^2 UGU^T)(UQU^T)^{-1} + o(\varepsilon^d). \tag{65}$$

As a result, the matrix C from (14) has the form

$$\begin{aligned} C &= \Sigma_{ZY}\Sigma_{YY}^{-1} \\ &= QU^T(UQU^T)^{-1} \\ &\quad + \varepsilon^d\Big(\big(P + \delta G\big)U^T(UQU^T)^{-1} \\ &\qquad - QU^T(UQU^T)^{-1}U\big(P + \delta G\big)U^T(UQU^T)^{-1}\Big) + o(\varepsilon^d). \end{aligned} \tag{66}$$

6.4 Beyond mean field expansion: quadratic term

Recall that matrix D in (13) has form (15) similar to the form of C, with Y replaced by $s(Y)$. Matrix Σ_{SS} has entries given by (17). Normally, we expect that

$$\Sigma_{SS} = \Sigma_{SS}^{(0)} + \varepsilon^d \Sigma_{SS}^{(1)} + o(\varepsilon^d) = O(1)$$

for some ε-independent matrices $\Sigma_{SS}^{(0)}, \Sigma_{SS}^{(1)} \in \mathcal{M}_{\varkappa,\varkappa}$; we assume additionally that $\Sigma_{SS}^{(0)}$ is invertible. Note that $\Sigma_{SS}^{(0)} = \lim_{\varepsilon\to 0} \Sigma_{SS}$ is symmetric and positive semidefinite, being a limit of the (symmetric positive semidefinite) covariance matrices Σ_{SS}; hence, its invertibility automatically means that $\Sigma_{SS}^{(0)}$ is positive definite. In particular, for all sufficiently small $\varepsilon > 0$, the smallest eigenvalue of Σ_{SS} is bounded away from zero, and

$$(\Sigma_{SS})^{-1} = \big(\Sigma_{SS}^{(0)}\big)^{-1} + O(\varepsilon^d) = O(1).$$

Next, note that

$$\mathbb{E}[\|s(Y)\|^2] = \mathbb{E}[s(Y)^T s(Y)] = \operatorname{tr} \Sigma_{SS}$$

(here the norm is taken in $\mathbb{R}^\varkappa$). Thus, $s(Y) = O_{L^2}(1)$, where we write $\xi = O_{L^2}(\varepsilon^\gamma)$ (respectively, $\xi = o_{L^2}(\varepsilon^\gamma)$) for a random vector ξ with $\mathbb{E}[\|\xi\|^2]^{1/2} = O(\varepsilon^\gamma)$ (respectively, $= o(\varepsilon^\gamma)$).

Moreover, for $r_2(Y) = Ds(Y) = \Sigma_{ZS}(\Sigma_{SS})^{-1}s(Y)$, we have, denoting $\Sigma_{SZ} := (\Sigma_{ZS})^T$, the exact identity

$$\mathbb{E}\big[\|r_2(Y)\|^2\big] = \operatorname{tr}\big(D\,\mathbb{E}[s(Y)s(Y)^T]D^T\big) = \operatorname{tr}\big(\Sigma_{ZS}\Sigma_{SS}^{-1}\Sigma_{SZ}\big),$$

whence

$$\|\Sigma_{SS}\|^{-1}\,\|\Sigma_{ZS}\|_{\mathrm{HS}}^2 \le \mathbb{E}\big[\|r_2(Y)\|^2\big] \le \|\Sigma_{SS}^{-1}\|\,\|\Sigma_{ZS}\|_{\mathrm{HS}}^2,$$

where $\|\cdot\|$ denotes the operator norm and $\|\cdot\|_{\mathrm{HS}}$ the Hilbert–Schmidt norm. Since, by the above, both $\|\Sigma_{SS}\|$ and $\|\Sigma_{SS}^{-1}\|$ are $O(1)$, the order in ε of $r_2(Y)$ in L^2 is exactly the order of $\|\Sigma_{ZS}\|_{\mathrm{HS}}$.

(Note also that $\Sigma_{SS} = O(\varepsilon^\alpha)$, $\alpha > 0$ would imply then that $s(Y) = O_{L^2}(\varepsilon^{\frac{\alpha}{2}})$, so $(\Sigma_{SS})^{-1}s(Y) = O_{L^2}(\varepsilon^{-\frac{\alpha}{2}})$, and Σ_{ZS} would need to have an additional smallness in ε.)

The problem is, however, that, by (16), Σ_{ZS} may have order $O(1)$. In principle, it depends on U. Indeed, under the assumption (60), the third moments $\mathbb{E}[\widetilde{Y_i}\widetilde{Y_j}\widetilde{Y_r}]$, being linear combinations of the third moments of $\widetilde{Z}$, have the order $O(1)$, with the leading term of order δ^{-2} coming from the "diagonal" moments $\mathbb{E}[\widetilde{Z}_a^3]$. Hence, if U is such that $C = O(1)$, i.e. if $QU^T(UQU^T)^{-1}$ does not "disappear", then $\Sigma_{ZS} = O(1)$ unless some cancellation happens. Therefore, $r_2(Y) = O(1)$ as well.

6.4.1 Case study 1

Let $U = (\mathbf{O}\ \mathbf{I})$, where $\mathbf{O}$ is the zero matrix from $\mathcal{M}_{k,n-k}$, and $\mathbf{I}$ is the unit matrix from $\mathcal{M}_{k,k}$. Then $Z = (X^T\ Y^T)^T$, where $X \in \mathbb{R}^{n-k}$ is the unobserved part and $Y \in \mathbb{R}^k$ is the observed part (the last k coordinates of Z). Moreover,

$$\mathbb{E}[Z \mid Y] = \big(\mathbb{E}[X \mid Y]^T \;\; Y^T\big)^T,$$

and to calculate $\mathbb{E}[X \mid Y]$ we can use the same formulas as above, with the crucial difference that $\operatorname{Cov}(X, Y)$ now does not contain the main diagonal elements, as X and Y are "disjoint", i.e.

$$\Sigma_{XY} = \varepsilon^d G_{XY} + o(\varepsilon^d),$$

where $G_{XY} \in \mathcal{M}_{n-k,k}$ is the corresponding off-diagonal block of G. Denote also by $Q_Y \in \mathcal{M}_{k,k}$ the (last) diagonal block of Q corresponding to the coordinates of Y; then $UQU^T = Q_Y$ and, by the above,

$$\Sigma_{YY}^{-1} = \delta Q_Y^{-1} + O(\varepsilon^d).$$

Then the corresponding matrix (14), computed for the pair (X, Y) and denoted here by C_X, satisfies

$$C_X = \Sigma_{XY}\Sigma_{YY}^{-1} = \varepsilon^d G_{XY}\big(\delta Q_Y^{-1} + O(\varepsilon^d)\big) = \varepsilon^d \delta\, G_{XY} Q_Y^{-1} + o(\varepsilon^d),$$

hence,

$$\begin{aligned} \mathbb{E}[X \mid Y] &= \mathbb{E}[X] + \Sigma_{XY}\Sigma_{YY}^{-1}(Y - \mathbb{E}[Y]) + r_2(Y) + \dots \\ &= q_X + \varepsilon^d p_X + \varepsilon^d \delta\, G_{XY} Q_Y^{-1}(Y - \mathbb{E}[Y]) + o_{L^2}(\varepsilon^d) + r_2(Y) + \dots, \end{aligned} \tag{67}$$

where $q_X, p_X \in \mathbb{R}^{n-k}$ are the subvectors of q, p corresponding to the coordinates of X.

Note that here $C_X = O(\varepsilon^d)$ because, in (66) applied to the full vector Z,

$$QU^T(UQU^T)^{-1} = (\mathbf{O}\ I_k)^T,$$

whose first $n-k$ rows, i.e. the rows corresponding to X, vanish.

Next, by (16),

$$(\Sigma_{XS})_{l,\langle i,j\rangle} = o(\varepsilon^d), \qquad 1 \le l \le n-k,\ 1 \le j \le i \le k. \tag{68}$$

Indeed, by (60),

$$\mathbb{E}[\widetilde{X}_l \widetilde{Y}_i \widetilde{Y}_j] = \delta_{i,j}\, \delta^{-1} \operatorname{Cov}(X_l, Y_i) + o(\varepsilon^d) = \delta_{i,j}\, \varepsilon^d \delta^{-1} (G_{XY})_{li} + o(\varepsilon^d)$$

and

$$\mathbb{E}[\widetilde{Y}_i \widetilde{Y}_j \widetilde{Y}_r] = \delta_{i,j,r} \frac{(q_Y)_i + \varepsilon^d (p_Y)_i}{\delta^2} + O(\varepsilon^d),$$

where $\delta_{i,j}$ (respectively, $\delta_{i,j,r}$) equals 1 if all the indicated indices coincide and 0 otherwise, and $q_Y, p_Y \in \mathbb{R}^k$ are the subvectors of q, p corresponding to the coordinates of Y. Since, by the above,

$$(C_X)_{lr} = \varepsilon^d \delta\, (G_{XY} Q_Y^{-1})_{lr} + o(\varepsilon^d) = \varepsilon^d \delta\, (G_{XY})_{lr} (q_Y)_r^{-1} + o(\varepsilon^d),$$

we obtain:

$$\sum_{r=1}^{k} \mathbb{E}[\widetilde{Y}_i \widetilde{Y}_j \widetilde{Y}_r](C_X)_{lr} = \delta_{i,j}\, \varepsilon^d \delta^{-1} (G_{XY})_{li} + o(\varepsilon^d),$$

that exactly cancels $\mathbb{E}[\widetilde{X}_l \widetilde{Y}_i \widetilde{Y}_j]$; this proves (68). As a result, provided that $\Sigma_{SS}^{(0)}$ is invertible, $r_2(Y) = o_{L^2}(\varepsilon^d)$.
