# Supplementary material for: R-package agentBayes: Likelihood-based statistical methods for agent-based models
Source: PLoS Comput Biol. 2026 Sep 15;22(9):e1014786. doi: 10.1371/journal.pcbi.1014786 (PMC13619099; doi:10.1371/journal.pcbi.1014786)
Supplement: Supplementary file 2 [file pcbi.1014786.s003.docx]

PCOMPBIOL-D-26-00922
R-package agentBayes: likelihood-based statistical methods for agent-based models
PLOS Computational Biology

Dear Editor,

Thank you for the feedback and for the opportunity to resubmit our manuscript PCOMPBIOL-D-26-00922. We have carefully addressed all comments from the reviewers, which we believe have helped improve the quality and clarity of our manuscript.

The main revisions concern the framing of the methodological contribution and the scope of the proposed modelling framework. In particular, we have now adjusted the framing of the manuscript in the introduction and discussion to clarify 1) the contribution of our work in relation to previous developments of the RCP framework and existing likelihood-based approaches for ABMs; 2) the generality and limitations of the proposed framework to better align the manuscript’s claims with its scope and contributions; 3) the applicability of the framework, including an expanded discussion of parameter identifiability and a simulation validation for the third case study.

Below, we address the reviewers’ comments point-by-point with the original comments shown in black and our responses in blue. We have uploaded both a marked-up version of the manuscript with tracked changes and a version without tracked changes. We have also incorporated the instructions listed under the journal requirements.

We hope that you find the revised manuscript acceptable for publication in *PLOS Computational Biology*.

Sincerely,

Niklas Moser

On behalf of all authors

----------------------------------------------------------

Reviewer #1: The paper presents agentBayes, an R package for likelihood-based inference in agent-based models written as continuous-space and continuous-time RCP models. The key construction is an asymptotic likelihood, built from perturbative expressions for densities, variances, and spatial cumulants, then used for Bayesian parameter inference on simulated systems and cancer cell data.

There is a lot of real mathematics here. I found the work technically ambitious, and the software angle is also valuable. Automating these derivations is a serious contribution: in past work, the likelihood is derived by hand, which prevents most ABMs users from using likelihood-based methods (still, the past literature on likelihood-based inference in ABMs should be mentioned more carefully).

We thank the reviewer very much for the positive feedback.

My recommendation is major revision.

The main issue is the way the contribution is framed. Some claims read as if the paper gives a general likelihood theory for ABMs, while I do not think the manuscript supports that. The theory applies to continuous-space, continuous-time RCP models. This is a meaningful class, and probably a large one in some fields. Still, many standard ABMs use discrete time, discrete space, and synchronous updating. These are central examples of ABMs for many readers. The manuscript should make this scope visible consistently.

We thank the reviewer for this important comment. We agree with the reviewer that our framework does not encompass all types of ABMs, particularly discrete time, discrete space and synchronous updating. We have revised the manuscript to make this scope more explicit. We would like to emphasize that continuous space and continuous time RCP models represent an important class of models in many real-world applications, particularly in biology, where underlying processes such as interactions, dispersal, movement and competition operate asynchronously in continuous space and continuous time.

For instance, sentences such as “The framework presented in this paper is to our knowledge the first mathematically rigorous likelihood-based unifying statistical theory of ABMs” and “derive an asymptotically exact expression for the likelihood of ABMs” are overselling a result that has important limitations. The paper can still be very important with this narrower framing.

We thank the reviewer for pointing out these examples. We have adjusted the framing of these sentences in the manuscript. Particularly, we have changed “The framework presented in this paper is to our knowledge the first mathematically rigorous likelihood-based unifying statistical theory of ABMs” to “The framework presented in this paper is to our knowledge the first mathematically rigorous likelihood-based unifying statistical theory that applies to a broad class of ABMs”, and “derive an asymptotically exact expression for the likelihood of ABMs” to “derive an asymptotically exact expression for the likelihood of RCP-models, which represent a broad class of ABMs”.

On the same note, the case studies are useful and Figure 2 is especially important. Still, they mainly concern spatial birth-death-competition systems, and I am not convinced that these examples show representativeness for ABMs in the broad sense, i.e., they are not classical examples used for ABMs in general.

We thank the reviewer for the positive feedback on the case studies and Figure 2. We agree that the case studies exemplify variations of spatial birth-death-competition systems and therefore do not illustrate the full variety of processes that can be formulated within the RCP framework. We have added a remark in the discussion that guides the reader to a more exhaustive process library for RCP models. Let us note here that RCP models can incorporate many types of processes, including movement of agents, evolutionary processes through changes of agent marks with functional consequences and interactions with the environmental variables or habitats. We would also like to clarify that the model used in case studies one and two were selected as established examples from the spatiotemporal point process and theoretical ecology literature, e.g. [1,2]. The aim of the case studies was to demonstrate the validity of the developed method using synthetic/simulated data from an established model (case studies one and two), and to demonstrate the applicability of the method for addressing biological research questions using real-world empirical data.

Minor concerns:

* I would like the authors to explain where the first-order approximation is expected to fail. The examples show that it works in the chosen settings. They do not give me much intuition about the edge of the method.

We agree with the reviewer that the discussion of the perturbation theory is condensed in the current manuscript. We would like to indicate that a more extensive discussion of the perturbation theory has been provided in earlier publications of the RCP framework [3,4]. To provide additional context, the perturbation theory becomes exact in the limit of long-ranged interactions, corresponding to the mean-field limit when the scaling parameter of the interactions $\varepsilon\to0$. Consequently, the accuracy of the approximation depends on the degree of locality of interactions. In the publications introducing this theory, it is typically assumed that $\varepsilon\in[0,1]$, and in our case study we have deliberately chosen $\varepsilon=1$ representing a regime far from the mean-field limit. The first-order approximation is expected to become less accurate when higher-order cumulants are not sufficiently small. This may occur in systems exhibiting strong hierarchical clustering (many different levels of patches which organize the agents), strong segregation or strong regular clusters. The precise performance of the approximation, however, depends on the specific model and data. We would also like to note that we tested the method under several challenging initial conditions, far from complete spatial randomness (mean-field limit), including strong geometric structures such as ring-shaped arrangements of agents. In these tests, we observed that the framework was still able to recover the true parameter values.

* The cancer cell example is interesting and gives the paper a real application. It remains an application, though. It cannot check parameter recovery, since the true parameters are unknown.

We thank the reviewer for this important clarification. We agree that the cancer example, as an application to empirical data, cannot assess parameter recovery because the true parameter values are unknown. As recommended by reviewer 2, we included a simulated validation of the method for the cancer model in the Supplementary Information.

* The paper should say more about practical identifiability. I would especially worry about tradeoffs between interaction strength and interaction range. Maybe this is already well understood in the RCP literature. If so, the manuscript should point the reader there.

We thank the reviewer for pointing out this important topic. It motivated us to reframe the parts of case study one and two that already provide insights parameter identifiability (the motivation for comparing raw and derived parameters). In response to a related comment of reviewer 2, we have added for case study one and two posterior pair plots to the Supplementary Information to illustrate parameter correlations and potential trade-offs.

* The discussion of previous likelihood-based work on ABMs should be tightened. There are already papers that build likelihoods for ABMs, often in a model-specific way. The distinctive part here is the automated construction for RCP models.

We thank the reviewer for this comment and agree that previous studies have derived likelihoods for case- and data-specific ABMs. We have revised the introduction and discussion to better acknowledge these important contributions and clarify the distinction from our approach. While we agree with the reviewer that the automated construction for RCP models is one of the distinctive parts of our contribution, we would like to briefly clarify the other parts. Our approach is distinctive in terms of generality in two ways: 1) it incorporates a variety of ABMs that can be formulated as RCP models; 2) we have developed likelihoods for a variety of data types, including spatial snapshot and time-series data on single and multi-type agents that have been individually tracked or not and with known or unknown initial conditions. Furthermore, a central contribution of our work is the derivation of properties (i.e. expectation and covariance) of local agent densities, both unconditional and conditional on a variety of observational data. These expressions have not been derived for RCP models before. We have clarified the explanation on the contribution of our work in the manuscript.

References:

[1] Bolker, B. & Pacala, S. W. (1997). Using Moment Equations to Understand Stochastically Driven Spatial Pattern Formation in Ecological Systems. *Theor. Popul. Biol.* **52**, 179–197.

[2] Law R & Dieckmann U (2000). Moment Approximations of Individual-based Models. In: The Geometry of Ecological Interactions: Simplifying Spatial Complexity, eds. Dieckmann U, Law R & Metz JAJ, pp. 252–270. Cambridge University Press

[3] Ovaskainen, O. *et al.* (2014). A general mathematical framework for the analysis of spatiotemporal point processes. *Theor. Ecol.* **7**, 101–113.

[4] Cornell, S. J., Suprunenko, Y. F., Finkelshtein, D., Somervuo, P. & Ovaskainen, O. (2019). A unified framework for analysis of individual-based models in ecology and beyond. *Nat. Commun.* **10**, 4716.

Reviewer #2: The paper proposes a novel likelihood-based framework for continues time space and time agent-based models that builds upon the framework of the Reactant-Catalyst-Product (RCP) (from ref 36 by the same group). The method uses a perturbational approximation to mean and covariance of the number of particles in different plots, to derive an approximate likelihood function for agent-based models (ABMs) using Poisson or Normal statistics, enabling efficient inference of the parameters governing agent interactions. The approach is applied to two synthetic datasets and one real cancer dataset. The methodology is incorporated in the R-package agentBayes. Efficient inference for agent-based models are an important problem and this paper contributes a well presented and useful toolkit contributes to this area. Overall, the paper is well written and clear and the application to real world situation is great.

We would like to thank the reviewer for the encouraging words.

Before publications, I would recommend the authors to consider these comments:

- In the introduction, please specify more clearly what is the novel contribution in this manuscript compared to prior work from the same group on RCP (ref 36), is this only the package and the new applications? It seems to me that the conditional expectation equation 4 is obtained in this paper.

We thank the reviewer for this comment. Together with the comments from reviewer 1, it made us realize that the manuscript needed a more precise description of its contribution and novelty. We have therefore clarified the corresponding paragraph in the introduction. With respect to earlier publications, we clarify now that the expressions for the predictions of local agent densities and covariances unconditional and conditional on observational data (i.e. Equations 1-4) have, to the best of our knowledge, not previously been derived for RCP models.

- Regarding the case studies, I have the following questions: How has the prior distributions been chosen. It seems that the mode of the prior distribution is not matched with the true value in all cases (for example A+ in case study 1) and this can contribute to the bias in the posterior estimate.

We chose the priors for the synthetic data examples deliberately such that their modes do not necessarily coincide with the true parameter values. This is to mimic a more realistic inference setting, e.g. with real-world data for which typically the underlying parameter values are unknown, and prior information can hence be biased. This choice allows us to assess whether the method can recover the true parameter values even when the prior is not centred on the true values. We agree that the choice of prior can influence the posterior distributions and have added an explanation of prior choice to the manuscript.

- The non-spatial model performs better for some of the parameters which is puzzling, can this be explained?

We thank the reviewer for this important observation. We think this might be related to parameter identifiability. Referring to Fig. 3d, the non-spatial model covers the true parameter values for the density-independent mortality $m$ and the mortality due to competition $A^{-}$ within the interquartile range of the posterior, whereas this is not the case for the spatial models. However, the spatial models seem to still recover the correct total mortality ($m+A^{-}$) as they overestimate $m$ and underestimate $A^{-}$. Intuitively, the two parameters are inherently correlated when looking at the demographic dynamics in the data. It is hence our conclusion that they are not fully identifiable. This was one of the reasons we included the derived parameters, as we saw that those are identifiable. For the derived parameter $q^{*}$, non-spatial models do not perform better than spatial models. For the derived parameter $r$, the nonspatial models perform better than spatial models, this might be due to the spatial models overestimating the density-independent mortality $m$.

- The likelihood approach used is approximative and only exact in the limit of long-range interactions. How much of the error in the estimates comes from this kind of approximation. One possibility is to compare this results with inference based on ABC using the exact simulations to quantify the effect of the approximations in the likelihood.
We thank the reviewer for the valuable suggestion. The magnitude of the approximation error depends on the specific model, data and the extent to which higher-order cumulants contribute to the dynamics. The perturbation becomes exact If interactions are global as $\varepsilon\to0$. The validity of this perturbation expansion has been tested in previous publications, where $\varepsilon\in\left[ 0,1 \right]$. We have deliberately chosen the case of $\varepsilon=1$ in our case studies, i.e. regimes far from mean-field, and nevertheless observed good recovery of the data-generating parameters. We agree with the reviewer that a comparison with simulation-based inference methods such as ABC would provide additional benchmarking of our framework. However, such a comparison would constitute further substantial studying that is beyond the scope of the current manuscript. Furthermore, since ABC itself introduces approximation errors, e.g. through the choice of summary statistics, it would not be straightforward to design a test that isolates the effect of the approximation made in the perturbation expansion alone. Instead, a more direct analysis of the accuracy of the likelihood approximation can be conducted using likelihood profiles. Here, all parameters are set to the true value except for the parameter of interest, which is varied while evaluating the likelihood. The difference between the maximum of the likelihood profile and the true parameter value indicates the approximation error. We provide the likelihood profiles for the spatial snapshot example in the supporting information and compare different distributional assumptions in the R-package manual. We added a reference to these in the manuscript.

- Some of the remaining uncertainty in the parameters could be due to non-identifiability in the parameters so some pair plots would be good.

We thank the reviewer for this suggestion. We have added pair plots for case study one and two to the supporting information.

- Alternatively for case study one another parameter set up where epsilon is smaller and the likelihood is expected to be more exact could be performed and compared.

We thank the reviewer for this suggestion. We refer the reviewer to the explanation on the error of approximation made in the perturbation expansion.

- As the first two case studies with simulated data are on one-species examples, authors could consider simulating the third example with some of the inferred parameters as ground truth an apply their inference framework for further validation of the their results.

We thank the reviewer for this suggestion. We agree that a simulation case study based on multi-agent systems would provide additional validation of the framework in a more complex setting. We have added a validation test to the Supplementary Information, where we infer the parameters from data generated by one of the calibrated models. While the results show that 10 of the 11 parameters could be recovered, one of the length scales was estimated with a stronger bias. This shows that it remains challenging to estimate several length scales of processes from such data, and could hint to errors introduced through the approximation of the likelihood.

- Authors discuss some of the other approaches for efficient inference of agent-based models they do not discuss use of machine-learning enhanced simulation-based inference (e.g. see <https://doi.org/10.1371/journal.pcbi.1009508>)

We thank the reviewer for bringing this important part of literature to our attention. We have complemented our references to surrogate approaches in the introduction and the discussion with an additional reference to this literature.

- I noticed a typo in the abstract “… in the here introduced R-package …”

We thank the reviewer for pointing out this typo. We have corrected it in the revised manuscript.
