# Supplementary material for: R-package agentBayes: Likelihood-based statistical methods for agent-based models
Source: PLoS Comput Biol. 2026 Sep 15;22(9):e1014786. doi: 10.1371/journal.pcbi.1014786 (PMC13619099; doi:10.1371/journal.pcbi.1014786)
Supplement: Peer review history

# Peer review: Decision Letter 0

**DOI:** 10.1371/journal.pcbi.1014786.r001

**Authors:** Ovidiu Radulescu, Tobias Bollenbach

20 Jul 2026

PCOMPBIOL-D-26-00922

R-package agentBayes: likelihood-based statistical methods for agent-based models

PLOS Computational Biology

Dear Dr. Moser,

Thank you for submitting your manuscript to PLOS Computational Biology. After careful consideration, we feel that it has merit but does not fully meet PLOS Computational Biology's publication criteria as it currently stands. Therefore, we invite you to submit a revised version of the manuscript that addresses the points raised during the review process.

Please submit your revised manuscript by Sep 19 2026 11:59PM. If you will need more time than this to complete your revisions, please reply to this message or contact the journal office at ploscompbiol@plos.org. When you're ready to submit your revision, log on to https://www.editorialmanager.com/pcompbiol/ and select the 'Submissions Needing Revision' folder to locate your manuscript file.

Please include the following items when submitting your revised manuscript:

* A letter that responds to each point raised by the editor and reviewer(s). You should upload this letter as a separate file labeled 'Response to Reviewers'. This file does not need to include responses to formatting updates and technical items listed in the 'Journal Requirements' section below.

* A marked-up copy of your manuscript that highlights changes made to the original version. You should upload this as a separate file labeled 'Revised Manuscript with Track Changes'.

* An unmarked version of your revised paper without tracked changes. You should upload this as a separate file labeled 'Manuscript'.

If you would like to make changes to your financial disclosure, competing interests statement, or data availability statement, please make these updates within the submission form at the time of resubmission. Guidelines for resubmitting your figure files are available below the reviewer comments at the end of this letter. As the corresponding author, your ORCID iD is verified in the submission system and will appear in the published article. PLOS supports the use of ORCID, and we encourage all coauthors to register for an ORCID iD and use it as well. Please encourage your coauthors to verify their ORCID iD within the submission system before final acceptance, as unverified ORCID iDs will not appear in the published article. Only the individual author can complete the verification step; PLOS staff cannot verify ORCID iDs on behalf of authors. We look forward to receiving your revised manuscript.

Kind regards,

Ovidiu Radulescu, PhD

Academic Editor

PLOS Computational Biology

Tobias Bollenbach

Section Editor

PLOS Computational Biology

**Additional Editor Comments (if provided):**

**Journal Requirements:**

If the reviewer comments include a recommendation to cite specific previously published works, please review and evaluate these publications to determine whether they are relevant and should be cited. There is no requirement to cite these works unless the editor has indicated otherwise.

1) Please upload all main figures as separate Figure files in .tif or .eps format. For more information about how to convert and format your figure files please see our guidelines:

https://journals.plos.org/ploscompbiol/s/figures

2) Please upload a copy of Figures FIG 1, 2A-D, 3A-D, 4A-C, and 5A-L which you refer to in your text on pages 8, 12, 15, 16, and 18. Or, if the figure is no longer to be included as part of the submission please remove all reference to it within the text.

3) We have noticed that you have uploaded Supporting Information files, but you have not included a list of legends. Please add a full list of legends for your Supporting Information files after the references list.

4)Your current Financial Disclosure states, “The author(s) received no specific funding for this work". However, your funding information is Academy of Finland

336212

Otso Ovaskainen

Academy of Finland345110Otso OvaskainenHORIZON EUROPE European Research Council856506Otso OvaskainenWenner-Gren StiftelsernaWGF2022-0044Sara HamisKjell och Märta Beijers StiftelseSara HamisNorges Forskningsråd325628/IARDagim S. Tadele in the submission form that you did not receive funding. Please indicate by return email the full and correct funding information for your study and confirm the order in which funding contributions should appear. Please be sure to indicate whether the funders played any role in the study design, data collection and analysis, decision to publish, or preparation of the manuscript.

5)We have amended your Competing Interest statement to comply with journal style. We kindly ask that you double check the statement and let us know if anything is incorrect.

**Reviewers' comments:**

Reviewer's Responses to Questions

**Comments to the Authors:**

**Please note here if the review is uploaded as an attachment.**

Reviewer #1: The paper presents agentBayes, an R package for likelihood-based inference in agent-based models written as continuous-space and continuous-time RCP models. The key construction is an asymptotic likelihood, built from perturbative expressions for densities, variances, and spatial cumulants, then used for Bayesian parameter inference on simulated systems and cancer cell data.

There is a lot of real mathematics here. I found the work technically ambitious, and the software angle is also valuable. Automating these derivations is a serious contribution: in past work, the likelihood is derived by hand, which prevents most ABMs users from using likelihood-based methods (still, the past literature on likelihood-based inference in ABMs should be mentioned more carefully).

My recommendation is major revision.

The main issue is the way the contribution is framed. Some claims read as if the paper gives a general likelihood theory for ABMs, while I do not think the manuscript supports that. The theory applies to continuous-space, continuous-time RCP models. This is a meaningful class, and probably a large one in some fields. Still, many standard ABMs use discrete time, discrete space, and synchronous updating. These are central examples of ABMs for many readers. The manuscript should make this scope visible consistently. For instance, sentences such as “The framework presented in this paper is to our knowledge the first mathematically rigorous likelihood-based unifying statistical theory of ABMs” and “derive an asymptotically exact expression for the likelihood of ABMs” are overselling a result that has important limitations. The paper can still be very important with this narrower framing. On the same note, the case studies are useful and Figure 2 is especially important. Still, they mainly concern spatial birth-death-competition systems, and I am not convinced that these examples show representativeness for ABMs in the broad sense, i.e., they are not classical examples used for ABMs in general.

Minor concerns:

* I would like the authors to explain where the first-order approximation is expected to fail. The examples show that it works in the chosen settings. They do not give me much intuition about the edge of the method.

* The cancer cell example is interesting and gives the paper a real application. It remains an application, though. It cannot check parameter recovery, since the true parameters are unknown.

* The paper should say more about practical identifiability. I would especially worry about tradeoffs between interaction strength and interaction range. Maybe this is already well understood in the RCP literature. If so, the manuscript should point the reader there.

* The discussion of previous likelihood-based work on ABMs should be tightened. There are already papers that build likelihoods for ABMs, often in a model-specific way. The distinctive part here is the automated construction for RCP models.

Reviewer #2: The paper proposes a novel likelihood-based framework for continues time space and time agent-based models that builds upon the framework of the Reactant-Catalyst-Product (RCP) (from ref 36 by the same group). The method uses a perturbational approximation to mean and covariance of the number of particles in different plots, to derive an approximate likelihood function for agent-based models (ABMs) using Poisson or Normal statistics, enabling efficient inference of the parameters governing agent interactions. The approach is applied to two synthetic datasets and one real cancer dataset. The methodology is incorporated in the R-package agentBayes. Efficient inference for agent-based models are an important problem and this paper contributes a well presented and useful toolkit contributes to this area. Overall, the paper is well written and clear and the application to real world situation is great. Before publications, I would recommend the authors to consider these comments:

- In the introduction, please specify more clearly what is the novel contribution in this manuscript compared to prior work from the same group on RCP (ref 36), is this only the package and the new applications? It seems to me that the conditional expectation equation 4 is obtained in this paper.

- Regarding the case studies, I have the following questions: How has the prior distributions been chosen. It seems that the mode of the prior distribution is not matched with the true value in all cases (for example A+ in case study 1) and this can contribute to the bias in the posterior estimate.

- The non-spatial model performs better for some of the parameters which is puzzling, can this be explained?

- The likelihood approach used is approximative and only exact in the limit of long-range interactions. How much of the error in the estimates comes from this kind of approximation. One possibility is to compare this results with inference based on ABC using the exact simulations to quantify the effect of the approximations in the likelihood.

- Some of the remaining uncertainty in the parameters could be due to non-identifiability in the parameters so some pair plots would be good.

- Alternatively for case study one another parameter set up where epsilon is smaller and the likelihood is expected to be more exact could be performed and compared.

- As the first two case studies with simulated data are on one-species examples, authors could consider simulating the third example with some of the inferred parameters as ground truth an apply their inference framework for further validation of the their results.

- Authors discuss some of the other approaches for efficient inference of agent-based models they do not discuss use of machine-learning enhanced simulation-based inference (e.g. see https://doi.org/10.1371/journal.pcbi.1009508)

- I noticed a typo in the abstract “… in the here introduced R-package …”

**********

**Have the authors made all data and (if applicable) computational code underlying the findings in their manuscript fully available?**

The PLOS Data policy requires authors to make all data and code underlying the findings described in their manuscript fully available without restriction, with rare exception (please refer to the Data Availability Statement in the manuscript PDF file). The data and code should be provided as part of the manuscript or its supporting information, or deposited to a public repository. For example, in addition to summary statistics, the data points behind means, medians and variance measures should be available. If there are restrictions on publicly sharing data or code —e.g. participant privacy or use of data from a third party—those must be specified.

Reviewer #1: Yes

Reviewer #2: Yes

**********

PLOS authors have the option to publish the peer review history of their article (what does this mean?). If published, this will include your full peer review and any attached files.

If you choose “no”, your identity will remain anonymous but your review may still be made public.

**Do you want your identity to be public for this peer review?**  For information about this choice, including consent withdrawal, please see our Privacy Policy.

Reviewer #1: No

Reviewer #2: No

[NOTE: If reviewer comments were submitted as an attachment file, they will be attached to this email and accessible via the submission site. Please log into your account, locate the manuscript record, and check for the action link "View Attachments". If this link does not appear, there are no attachment files.]

**Figure resubmission:**

While revising your submission, we strongly recommend that you use PLOS’s NAAS tool (https://ngplosjournals.pagemajik.ai/artanalysis) to test your figure files. NAAS can convert your figure files to the TIFF file type and meet basic requirements (such as print size, resolution), or provide you with a report on issues that do not meet our requirements and that NAAS cannot fix.

After uploading your figures to PLOS’s NAAS tool - https://ngplosjournals.pagemajik.ai/artanalysis, NAAS will process the files provided and display the results in the "Uploaded Files" section of the page as the processing is complete. If the uploaded figures meet our requirements (or NAAS is able to fix the files to meet our requirements), the figure will be marked as "fixed" above. If NAAS is unable to fix the files, a red "failed" label will appear above. When NAAS has confirmed that the figure files meet our requirements, please download the file via the download option, and include these NAAS processed figure files when submitting your revised manuscript.

**Reproducibility:**

To enhance the reproducibility of your results, we recommend that authors of applicable studies deposit laboratory protocols in protocols.io, where a protocol can be assigned its own identifier (DOI) such that it can be cited independently in the future. Additionally, PLOS ONE offers an option to publish peer-reviewed clinical study protocols. Read more information on sharing protocols at https://plos.org/protocols?utm_medium=editorial-email&utm_source=authorletters&utm_campaign=protocols

# Author comment: Author response to Decision Letter 1

**DOI:** 10.1371/journal.pcbi.1014786.r002

10 Aug 2026

Attachment Submitted filename: ResponseLetter.docx

# Peer review: Decision Letter 1

**DOI:** 10.1371/journal.pcbi.1014786.r003

**Authors:** Ovidiu Radulescu, Tobias Bollenbach

1 Sep 2026

Dear Mr. Moser,

We are pleased to inform you that your manuscript 'R-package agentBayes: likelihood-based statistical methods for agent-based models' has been provisionally accepted for publication in PLOS Computational Biology.

Before your manuscript can be formally accepted you will need to complete some formatting changes, which you will receive in a follow up email. A member of our team will be in touch with a set of requests.

Please note that your manuscript will not be scheduled for publication until you have made the required changes, so a swift response is appreciated.

IMPORTANT: The editorial review process is now complete. PLOS will only permit corrections to spelling, formatting or significant scientific errors from this point onwards. Requests for major changes, or any which affect the scientific understanding of your work, will cause delays to the publication date of your manuscript.

Should you, your institution's press office or the journal office choose to press release your paper, you will automatically be opted out of early publication. We ask that you notify us now if you or your institution is planning to press release the article. All press must be co-ordinated with PLOS.

Thank you again for supporting Open Access publishing; we are looking forward to publishing your work in PLOS Computational Biology.

Best regards,

Ovidiu Radulescu, PhD

Academic Editor

PLOS Computational Biology

Tobias Bollenbach

Section Editor

PLOS Computational Biology

***********************************************************

Reviewer's Responses to Questions

**Comments to the Authors:**

**Please note here if the review is uploaded as an attachment.**

Reviewer #1: The authors have addressed the main concerns raised in the previous review. I particularly appreciate the revised framing of the contribution, which now better reflects the scope of the framework and its connection to continuous-space, continuous-time RCP models. The additional validation analyses are also useful, especially the supplementary parameter recovery analysis for the cancer model, which provides further evidence that the framework can recover model parameters in a realistic application setting.

Overall, I believe the manuscript is ready for acceptance. The mathematical framework is technically strong, the methodological contribution is valuable, and the revised manuscript now presents the scope of the approach more clearly. I believe that the method proposed by the authors could be useful to the community and I would be glad to see it published.

I also appreciate the authors’ clarification regarding the applicability of the perturbation approximation. I found this point useful for understanding the limits of applicability of the framework. At the same time, in their response, the authors discuss that the framework was tested in regimes far from the mean-field limit, where the perturbation expansion is far from its asymptotic regime, and that the method was still able to recover the data-generating parameters. I find this result encouraging, although its interpretation is not immediately clear in terms of defining the practical applicability regime of the approximation. After considering both the authors’ response and the discussion now provided in the manuscript, particularly regarding the assumptions of the framework and the conditions under which it may become less accurate, I believe that this point has been adequately addressed. As such, I have no further major concerns and recommend acceptance.

Reviewer #2: Authors have revised their papers according to the comments in a satisfactory manner.

**********

**Have the authors made all data and (if applicable) computational code underlying the findings in their manuscript fully available?**

The PLOS Data policy requires authors to make all data and code underlying the findings described in their manuscript fully available without restriction, with rare exception (please refer to the Data Availability Statement in the manuscript PDF file). The data and code should be provided as part of the manuscript or its supporting information, or deposited to a public repository. For example, in addition to summary statistics, the data points behind means, medians and variance measures should be available. If there are restrictions on publicly sharing data or code —e.g. participant privacy or use of data from a third party—those must be specified.

Reviewer #1: Yes

Reviewer #2: Yes

**********

PLOS authors have the option to publish the peer review history of their article (what does this mean?). If published, this will include your full peer review and any attached files.

If you choose “no”, your identity will remain anonymous but your review may still be made public.

**Do you want your identity to be public for this peer review?**  For information about this choice, including consent withdrawal, please see our Privacy Policy.

Reviewer #1: No

Reviewer #2: No

# Editor report: Acceptance letter

**DOI:** 10.1371/journal.pcbi.1014786.r004

**Authors:** Ovidiu Radulescu, Tobias Bollenbach

PCOMPBIOL-D-26-00922R1

R-package agentBayes: likelihood-based statistical methods for agent-based models

Dear Dr Moser,

I am pleased to inform you that your manuscript has been formally accepted for publication in PLOS Computational Biology. Your manuscript is now with our production department and you will be notified of the publication date in due course.

The corresponding author will soon be receiving a typeset proof for review, to ensure errors have not been introduced during production. Please review the PDF proof of your manuscript carefully, as this is the last chance to correct any errors. Please note that major changes, or those which affect the scientific understanding of the work, will likely cause delays to the publication date of your manuscript.

Soon after your final files are uploaded, unless you have opted out, the early version of your manuscript will be published online. The date of the early version will be your article's publication date. The final article will be published to the same URL, and all versions of the paper will be accessible to readers.

For Research, Software, and Methods articles, you will receive an invoice from PLOS for your publication fee after your manuscript has reached the completed accept phase. If you receive an email requesting payment before acceptance or for any other service, this may be a phishing scheme. Learn how to identify phishing emails and protect your accounts at https://explore.plos.org/phishing.

Thank you again for supporting PLOS Computational Biology and open-access publishing. We are looking forward to publishing your work!

With kind regards,

Sharmila Kamatchi

PLOS Computational Biology | Carlyle House, Carlyle Road, Cambridge CB4 3DN | United Kingdom ploscompbiol@plos.org | Phone +44 (0) 1223-442824 | ploscompbiol.org | @PLOSCompBiol
